# What genes are differentially expressed in individuals with schizophrenia? A systematic review

**DOI:** 10.1038/s41380-021-01420-7

**Published:** 2022-01-28

**Authors:** Alison K. Merikangas, Matthew Shelly, Alexys Knighton, Nicholas Kotler, Nicole Tanenbaum, Laura Almasy

**Affiliations:** 1 Department of Biomedical and Health Informatics, Children’s Hospital of Philadelphia, Philadelphia, PA USA; 2 Department of Genetics, Perelman School of Medicine, University of Pennsylvania, Philadelphia, PA USA; 3 Lifespan Brain Institute, Children’s Hospital of Philadelphia and Perelman School of Medicine, University of Pennsylvania, Philadelphia, PA USA; 4 Department of Biology, College of Science and Engineering, Wilkes University, Wilkes-Barre, PA USA

**Keywords:** Schizophrenia, Diagnostic markers, Genetics

## Abstract

Schizophrenia is a severe, complex mental disorder characterized by a combination of positive symptoms, negative symptoms, and impaired cognitive function. Schizophrenia is highly heritable (~80%) with multifactorial etiology and complex polygenic genetic architecture. Despite the large number of genetic variants associated with schizophrenia, few causal variants have been established. Gaining insight into the mechanistic influences of these genetic variants may facilitate our ability to apply these findings to prevention and treatment. Though there have been more than 300 studies of gene expression in schizophrenia over the past 15 years, none of the studies have yielded consistent evidence for specific genes that contribute to schizophrenia risk. The aim of this work is to conduct a systematic review and synthesis of case–control studies of genome-wide gene expression in schizophrenia. Comprehensive literature searches were completed in PubMed, EmBase, and Web of Science, and after a systematic review of the studies, data were extracted from those that met the following inclusion criteria: human case–control studies comparing the genome-wide transcriptome of individuals diagnosed with schizophrenia to healthy controls published between January 1, 2000 and June 30, 2020 in the English language. Genes differentially expressed in cases were extracted from these studies, and overlapping genes were compared to previous research findings from the genome-wide association, structural variation, and tissue-expression studies. The transcriptome-wide analysis identified different genes than those previously reported in genome-wide association, exome sequencing, and structural variation studies of schizophrenia. Only one gene, GBP2, was replicated in five studies. Previous work has shown that this gene may play a role in immune function in the etiology of schizophrenia, which in turn could have implications for risk profiling, prevention, and treatment. This review highlights the methodological inconsistencies that impede valid meta-analyses and synthesis across studies. Standardization of the use of covariates, gene nomenclature, and methods for reporting results could enhance our understanding of the potential mechanisms through which genes exert their influence on the etiology of schizophrenia. Although these results are promising, collaborative efforts with harmonization of methodology will facilitate the identification of the role of genes underlying schizophrenia.

## Introduction

Schizophrenia is a severe, complex mental disorder characterized by a combination of positive symptoms (hallucinations and delusions, and psychotic symptoms in which there is a loss of contact with reality), negative symptoms (paucity of spontaneous speech, social withdrawal, and amotivation), and impaired cognitive function [1]. Overall, the sex ratio in prevalence is approximately equal, but with greater severity and earlier onset in males than in females [2]. Genetic epidemiologic studies have shown that schizophrenia is highly heritable (~80%) but with a multifactorial etiology and complex polygenic genetic architecture. There is evidence that both common and rare genetic variants, as well as diverse environmental factors contribute to its etiology [1]. Specific copy number variants (CNVs) have also been shown to be associated with schizophrenia [3].

Despite the large number of genetic variants that have been associated with schizophrenia, few causal variants have been established, and most genetic associations have not imparted useful clinical applications [1]. Moreover, many of the genome-wide associated variants are not found in genes, possibly indicating that they may have a regulatory role in modifying gene expression or are expression quantitative trait loci (eQTLs) [4]. Information on gene expression overall and the up- or downregulation of specific genes in cases as compared with unaffected controls, may provide novel information on the pathogenesis of schizophrenia. In addition, by examining gene expression in peripheral tissues, biomarkers for use in diagnosis or treatment may be found. Such peripheral biomarkers may be particularly valuable as the primary organ of interest, i.e., the brain, cannot be sampled in vivo [5].

Gene expression, the connection between the information encoded within a gene and the final functional protein, is the central concept of molecular biology. This complex, multistaged, highly regulated process involves the transcription of genes into mRNA followed by their translation into proteins [6, 7]. This process is influenced by both genetic and environmental factors. CNVs can modify gene expression through multiple potential mechanisms including the impact of gene duplication or deletion on dosage-sensitive genes, modification of regulatory elements, position effects, or regulation of normal flanking genes [8]. Differences in gene expression can be a biomarker or highlight a potential therapeutic target. While these changes may not always translate into consequential biological activity or be independently interpretable, this information can be combined with other information to highlight areas for additional research [9]. Altered gene expression has been associated with multiple disorders, such as Alzheimer’s disease [10], cancer [11], susceptibility to infection [12], and psychiatric disorders [13, 14], including schizophrenia [15]. There are also gene expression differences across tissues, sexes, ages, and genetic ancestry [16].

Techniques for assessing gene expression or transcript abundance have changed rapidly over the last 10–15 years. Advances in technologies characterizing transcript abundance levels, such as sequencing with RNAseq, and imputed expression based on tissue databases such as the Genotype-Tissue Expression (GTEx) project [17] have gradually supplanted the less expensive and less comprehensive microarray technology. Imputed gene expression has several advantages over microarray methods and RNAseq including larger sample sizes than directly measured expression, ability to target the tissue of interest for a particular disease or condition, and obviated need to control for environmental impacts, e.g., medication use or smoking.

There have been more than three hundred studies of gene expression in schizophrenia over the past 15 years, but to date there is no consistent evidence for clearly implicated genes from these findings. Moreover, though there have been several reviews of gene expression studies in schizophrenia (e.g., Bray [18], Harrison and Weinberger [19], Iwamoto and Kato [20], Kumarasinghe et al. [15], Mirnics et al. 2001 [21]), the majority were completed more than 10 years ago, and to date there has been no synthesis of the results across tissue sources and gene expression measurement technologies. Other limitations of many of the earlier studies include the use of candidate gene rather than genome-wide approaches; lack of unaffected controls; and failure to consider potential confounding factors, such as sex, age, and genetic ancestry [16]. Therefore, the overarching aim of this report is to complete a systematic review and synthesis of case–control studies of genome-wide gene expression in schizophrenia.

## Methods

### Scope of the review and PROSPERO registration

The scope of this systematic review is to identify genes that are differentially expressed in individuals with schizophrenia. The review was limited to noninterventional human studies that screen the genome in an unbiased manner. The review protocol was registered with PROSPERO [22], the International prospective register of systematic reviews (https://www.crd.york.ac.uk/PROSPERO/). The PRISMA checklist for this study is included in the supplementary materials (Supplementary Table 4).

### Stages of literature review

To maximize the number of potential studies included in the review, the initial searches were completed in three electronic bibliographic databases: (1) PubMed (https://pubmed.ncbi.nlm.nih.gov/); (2) EMBASE (https://www.embase.com/); and (3) Web of Science (https://login.webofknowledge.com/). The searches were limited to human case–control studies comparing the genome-wide transcriptome of individuals diagnosed with schizophrenia to healthy controls published between January 1, 2000 and June 30, 2020 in the English language. The schizophrenia case definition included patients with schizophrenia as recognized using any standard diagnostic criteria (Diagnostic and Statistical Manual of Mental Disorders (DSM), International Classification of Diseases (ICD), or clinician diagnosis). Study participants were considered healthy controls if they did not have a diagnosed mental disorder. The search syntax was customized for each database: PubMed: “Schizophrenia” [MeSH] and (“Transcriptome” [MeSH] or “Transcription” [MeSH] or “Sequence Analysis, RNA” [MeSH] or “Microarray Analysis” [MeSH]) and “humans” [MeSH Terms] and “case–control studies” [MeSH Terms]) and “humans” [MeSH Terms]; EmBase: (“schizophrenia”/exp OR schizophrenia) and (“transcriptome”/exp or transcriptome or “microarray analysis”/exp or “microarray analysis” or “RNA sequence”/exp or “RNA sequence” or “gene expression”/exp or “gene expression”) and (“case control study”/exp or “case control study”) and “human”/de and “article”/it; Web of Science: TS = (Schizophr* AND (Transcriptome or Transcription or “Microarray Analysis” or “RNA sequence”))) and LANGUAGE: (English) and DOCUMENT TYPES: (Article) Refined by: TOPIC: (case control) Timespan: 2000–2020.

After the literature searches were completed, the references were downloaded to the Rayyan systematic review web app [23] where duplicate references were identified and removed. Next, the titles and abstracts of the references were screened by two reviewers who assessed whether they met the inclusion and exclusion criteria. Any discrepancy in inclusion decisions was resolved by a third researcher.

Articles that met the inclusion criteria after the title and abstract review were then reviewed in full by three blind reviewers and assessed for inclusion in data extraction and analysis. Inclusion discrepancies were resolved through discussion and consensus of involved researchers.

Data were extracted from each study that met the a priori inclusion criteria. Specifically, sample size, including the number of cases and controls; diagnostic methodology; effect size, type, and statistical significance per gene; type of tissue in which gene expression was measured; technology used to quantify gene expression; demographic and other covariates, including sex, age, genetic ancestry, smoking status; and major findings from the study were extracted into a standardized data collection form.

Two of the reviewers assessed the risk of bias and quality in the included studies by considering the guidelines provided by the adapted Systematic Omics Analysis Review (SOAR) assessment [24]. Only a subset of the SOAR criteria applied to the studies presented here, notably, the “Preliminary Questions”, “Experimental Design”, “Human Subjects”, and “Microarray Data” sections. All studies provided human subjects information, but to our knowledge, none provided raw data files, and the data do not appear to be available in public repositories. However, data files may be available if requested from the authors.

### Data integration and analysis

To harmonize the data, gene nomenclature was standardized to HUGO Gene Nomenclature (https://www.genenames.org/) for each study. Each gene was classified as being up- or downregulated in cases as compared to controls, and statistically significant or not as defined by the authors of the study.

The data from each study were then combined into a single analytic file. The frequency of each statistically significant gene was calculated, and whether it was consistently up or downregulated in all studies was noted. Genes found to be differentially expressed in multiple studies were then compared to findings from the largest schizophrenia genome-wide association study (GWAS) to date (90 cohorts including 67,390 cases and 94,015 controls [25]) and to well-established schizophrenia-associated recurrent and non-recurrent CNVs [26]. We also queried genes found to be differentially expressed in multiple studies in the schizophrenia exome meta-analysis consortium database (SCHEMA, https://schema.broadinstitute.org/). SCHEMA aggregates high-throughput sequencing data of patients with schizophrenia and recorded whether these genes were found to be associated with specific classes of loss-of-function variants [27].

To examine differentially expressed genes in a biological context, we utilized the GENE2FUNC tool on the Functional Mapping and Annotation of Genome-Wide Association Studies (FUMA GWAS, https://fuma.ctglab.nl/) website [28]. This tool aggregates data from the GTEx project and allows the user to visualize the expression of a gene in various body tissues. Here, we obtained the average expression value per tissue type for each gene and the average of normalized expression per tissue type.

### Defining statistical significance

Each study tested a different number of transcripts, and several different statistical significance or false-discovery rate thresholds were applied. In many cases, the exact number of transcripts tested was not specified, making it difficult to rigorously evaluate the likelihood of differential transcription being reported for the same transcript by multiple studies. For purposes of estimation, we assumed testing of on the order of 20,000 transcripts in each study and we varied significance thresholds from a nominal 0.01 to a stricter 0.001. We then used a binomial model to assess the probability of a transcript being reported significant by three or more studies under the null model of no association with schizophrenia.

## Results

**Fig. 1:**
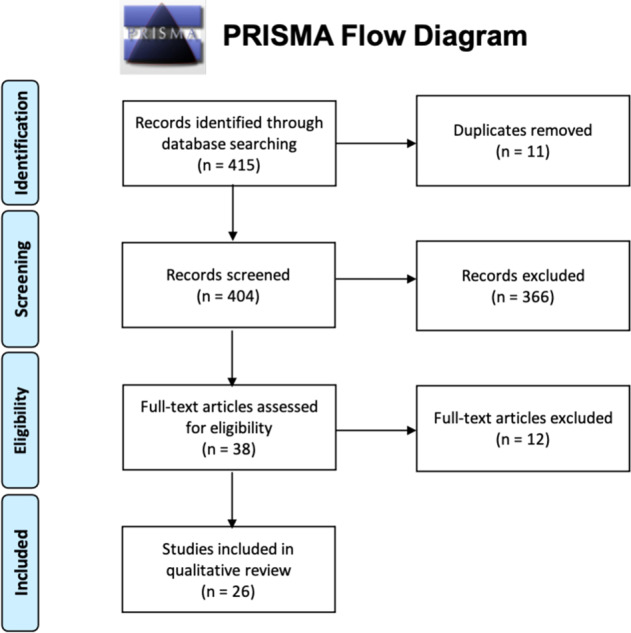
PRISMA flow diagram. The flow of information through the different phases of this systematic review, and it maps out the number of records identified, screened, included, and excluded.

As shown in the PRISMA flow diagram (Fig. 1), 415 records were identified through database searching. Of the 415, 11 were duplicate articles and were excluded before further consideration. Next, a total of 404 titles and abstracts were screened by two researchers to check if they met the study inclusion criteria (i.e., human case–control, noninterventional studies in English comparing the transcriptome of individuals diagnosed with schizophrenia to healthy controls). 366 abstracts did not meet the study inclusion criteria and were excluded. Articles were often excluded for more than one reason, and most excluded articles were candidate gene studies or not genome-wide (*n* = 334). Others were excluded because they did not meet the study criteria of examining a case–control comparison (*n* = 190); were completed in a non-schizophrenia sample (e.g., bipolar disorder patients) (*n* = 172); or did not actually examine gene expression (*n* = 26). Then, 12 of the 38 full-text articles that were assessed for data extraction eligibility did not meet study criteria and were excluded from further consideration. Specifically, five did not report a direct case–control comparison (Ellis et al. [29], Hagihara et al. [30], Kakiuchi et al. [31], Manchia et al. [32], Viana et al. [33]), one included previously reported data (Haroutunian et al. [34]), three were not genome-wide (Huang et al. [35], Katsel et al. [36], van Beveren et al. [37]), two did not report individual genes (Narla et al. [38], Yu et al. [39]) and one only reported results for bipolar disorder (Konradi et al. [40]). The remaining 26 articles were included in the data extraction stage, and were considered for synthesis, and qualitative review.

### Descriptive synthesis

Table 1 itemizes the studies included in our qualitative review. All studies reported in this review met the SOAR criteria for assessing quality and bias in omics studies reported above. A total of 18 studies assessed gene expression using microarrays, six utilized RNAseq and two used imputed gene expression based on genotype. In most of the studies (*n* = 12), gene expression was quantified from postmortem brain tissue (*n* = 2 amygdala; *n* = 4 dorsolateral prefrontal cortex (DLPFC); *n* = 3 hippocampus; *n* = 2 left hemisphere superior temporal cortex (STC); and *n* = 1 gyrus (STG)). Other studies measured transcription in blood samples (*n* = 10), induced pluripotent stem cell (IPSC)-derived neurons (*n* = 1), lymphoblastoid cell lines (LCLs) (*n* = 2), or skin fibroblasts (*n* = 1). One article (Collado-Torres et al. [41]) used data from the GTEx project to impute expression levels in two brain regions, so we have included these analyses as separate studies. The format for reporting effect size varied across studies, including beta, fold change or ratio, and fragments per kilobase per million mapped reads (FPKM). Correction for multiple comparisons also varied across studies, including a conservative Bonferroni, false-discovery rate (FDR), or no stated correction.

**Table 1:** Summary of the 26 manuscripts, describing 28 studies, included in this review.

Author	Year	*N*		Mean age (SD)		Sex (%F)		Method	Tissue	Notes
		HC	SZ	HC	SZ	HC	SZ			
Blood										
Bousman et al. [47]	2010	25	24	41 (9)	38 (8)	41.7	41.7	Microarray	Blood	Ingenuity*
Chen et al. [42]	2016	3	3	29.6 (12.3)	30.5 (12.9)	33.3	33.3	Microarray	Blood	lncRNA*
Gardiner et al. [48]	2012	76	112	37.8 (15.6)	40.7 (12.4)	53.4	39.1	Microarray	Blood	KEGG*
Kuzman et al. [103]	2009	32	32	28.2 (7.9)	28.2 (7.9)	75	75	Microarray	Blood	
Lee et al. [104]	2012	26	26	29.5	29.1	42.3	42.3	Microarray	Blood	
Leirer et al. [105]	2019	149	68	29.9 (10.5)	26.6 (7.7)	14	55	Microarray	Blood	
Wei et al. [49]	2015	400	564	27.4 (7.4)	26.2 (8.2)	54.4	52.1	Microarray	Blood	microRNA*
Wu et al. [44]	2016	49	47	28.8 (2)	39.6 (2.2)	51	42.5	Microarray	Blood	
Yu et al. [39]	2015	130	105	22.7 (6.8)	25.0 (8.3)	53.9	52.4	Microarray	Blood	microRNA*
Zhang et al. [50]	2015	2795	2570	36.2 (13.3)	33.5 (10.7)	63.9	48.3	Microarray	Blood	microRNA*
Brain										
Altar et al. [100]	2005	9	8	49.3 (6.7)	43.4 (15.5)	22.2	37.5	Microarray	HIPPO	Cohort 1*
	2005	15	14	47.2 (10.9)	43.3 (9.9)	26.7	28.6	Microarray	HIPPO	Cohort 2
Collado-Torres et al. [41]	2019	200	133	40.9	51.6	28.5	35.3	Imputed	HIPPO	
	2019	226	153	45	50.6	30.1	32	Imputed	DLPFC	
Harris et al. [89]	2008	13	14	42 (7.5)	43.8 (8.7)	23	35.7	Microarray	DLPFC	
Hauberg et al. [97]	2019	279	258	65.5	69	43	36	RNAseq	DLPFC	
Huckins et al. [43]	2019	65,264	40,299	NR	NR	NR	NR	Imputed	DLPFC	
Hwang et al. [92]	2013	15	14	48.1 (10.7)	43.6 (13)	40	35.7	RNAseq	HIPPO	
Liu et al. [106]	2018	27	22	34.2 (17.5)	43.6 (9.9)	22.2	13.6	RNAseq	Amygdala	
Mudge et al. [99]	2008	6	14	43.1 (9.2)	45.2 (11.8)	0	0	Microarray	Cerebellum	
Schmitt et al. [45]	2012	10	10	61.2 (14.6)	66.3 (12.0)	20	50	Microarray	STG	NS
Sellmann et al. [46]	2014	10	10	61.2 (14.6)	66.3 (12.0)	20	50	Microarray	STC	NS
Tian et al. [54]	2018	24	22	37.5 (15.4)	43.4 (10.1)	20	10	Microarray	Amygdala	NS
Wu et al. [107]	2012	9	9	44.2 (16.6)	44.3 (16.7)	0	0	RNAseq	STG	
Other										
Cattane et al. [108]	2015	20	20	48.4 (12.2)	44.6 (12.7)	55	50	Microarray	Skin fibroblast	
Lin et al. [109]	2016	7	8	34.4 (12.3)	31.5 (5.7)	28.5	50	RNAseq	IPSC	
Sanders et al. [110]	2017	660	529	43.6 (0.9)	46.5 (1)	54	50	RNAseq	LCL	
Sanders et al. [111]	2013	446	413	45.7 (1.2)	42.3 (1.5)	55	28.3	Microarray	LCL	

*DLPFC* dorsolateral prefrontal cortex, *HC* healthy control, *HIPPO* hippocampus, *F* female, *IPSC* induced pluripotent stem cell, *LCL* lymphoblastoid cell line, *lncRNA* long non-coding RNA, *NR* not reported, *NS* no statistically significant findings reported, *SZ* schizophrenia, *SD* standard deviation, *STC* superior temporal cortex, *STG* superior temporal gyrus.

*Excluded from data extraction.

The mean age of participants in each study ranged from 14 to 69 years in schizophrenia cases and 14 to 65 years in healthy controls. Included studies ranged from 0 to 75% female for both schizophrenia cases and healthy controls. The sample size varied substantially from as few as three cases and three controls [42] to over 40,000 cases and 65,000 controls [43]. In general, most studies had case/control samples that were well matched on age and sex, but there were a few studies where there was greater than the 10-year age difference between cases and controls (e.g., Collado-Torres et al. [41] and Wu et al. [44]). In a few instances, there was a substantial difference in the sex ratio between cases and controls, for example in the work presented by Schmitt et al. [45] and Sellmann et al. [46] the controls were 20% female, while the cases were 50% female.

### Replicated genes and direction of effect

Six articles that met full-text inclusion criteria were later excluded from data synthesis because the data could not be standardized or were unique to the individual study (Bousman et al. [47]; Chen et al. [42]; Gardiner et al. [48]; Wei et al. [49]; Yu et al. [39]; Zhang et al. [50]). Specifically, two studies completed pathway analyses, examining genes in aggregate instead of studying individual genes. Unfortunately, each of these studies used different pathway nomenclature; Bousman et al. [47] examined Ingenuity pathways, the only study to do so, while Gardiner [48] examined KEGG pathways. Thus, it was unclear whether findings overlapped between studies. Four other studies interrogated long non-coding RNAs (lncRNA, Chen et al. [42]) or microRNAs (miRNA, Wei et al. [49]; Yu et al. [39]; Zhang et al. [50]). Despite the growing evidence for their role in the regulation of gene expression [51, 52], and the potential use of lncRNAs as biomarkers for neuropsychiatric disorders [53], the studies that examined these variants were not included in the data synthesis presented here, because they used unique transcript identifiers that could not be directly mapped onto gene symbols or to one another.

Of the 20 remaining articles (21 studies), a total of 6771 unique genes were reported as statistically significantly differentially expressed in schizophrenia cases; 5566 reported in one study, 1045 in two, 138 reported in three, 21 in four, and one in five studies (Supplementary Table 1). Three studies reported that no genes reached statistical significance after correction for multiple comparisons (Schmitt et al. [45]; Sellmann et al. [46]; Tian et al. [54]). To narrow the focus of the results presented here, we chose to concentrate our analyses on the 160 genes that were reported three or more times across studies, as itemized in Table 2.

**Table 2:** 160 genes were reported three or more times across studies.

Gene	Count	Direction of Effect	Gene	Count	Direction of Effect	Gene	Count	Direction of Effect	Gene	Count	Direction of Effect	Gene	Count	Direction of Effect
GBP2	5	Inconsistent	CD24	3	Inconsistent	HIST1H2BD	3	Inconsistent	PPP1R2	3	Inconsistent	TM9SF2	3	Inconsistent
ATP1B1	4	Inconsistent	CD46	3	Consistent	HP	3	Consistent	PPP3CB	3	Inconsistent	TMEM204	3	Inconsistent
BIRC3	4	Inconsistent	CD47	3	Inconsistent	HSP90AB1	3	Inconsistent	PRR5	3	Inconsistent	TNNT1	3	Inconsistent
C2orf82	4	Inconsistent	CD82	3	Inconsistent	HSPA1A	3	Inconsistent	PTGS1	3	Consistent	TRIM22	3	Consistent
IFITM3	4	Consistent	CDKN1A	3	Inconsistent	HSPB1	3	Inconsistent	PUM1	3	Consistent	TRIM33	3	Inconsistent
KRAS	4	Consistent	CGGBP1	3	Inconsistent	IFITM1	3	Consistent	PVALB	3	Consistent	TTC14	3	Consistent
LRRC37A	4	Inconsistent	CHRNA2	3	Inconsistent	IFITM2	3	Consistent	RAB3IP	3	Consistent	UBE2G1	3	Consistent
MAP1LC3A	4	Inconsistent	CITED2	3	Consistent	INO80E	3	Consistent	RBCK1	3	Inconsistent	UBL7	3	Inconsistent
MARCH2	4	Inconsistent	CORO7	3	Consistent	ITGA5	3	Inconsistent	RBM12B	3	Inconsistent	UBQLN4	3	Inconsistent
MKNK2	4	Inconsistent	COX8C	3	Inconsistent	JAZF1	3	Inconsistent	REEP2	3	Inconsistent	UQCRH	3	Inconsistent
MOV10	4	Inconsistent	CPNE4	3	Inconsistent	JUND	3	Inconsistent	RERGL	3	Consistent	VAMP5	3	Consistent
MT2A	4	Inconsistent	CRYAB	3	Consistent	KANK3	3	Inconsistent	REXO1	3	Inconsistent	VPS4A	3	Inconsistent
MYCBP2	4	Inconsistent	CTSD	3	Inconsistent	LINC00634	3	Consistent	RNASE2	3	Consistent	XAF1	3	Consistent
RBM6	4	Inconsistent	DDX56	3	Consistent	LRRC37A2	3	Consistent	RPL35	3	Consistent	XBP1	3	Inconsistent
RPS5	4	Inconsistent	DHRS11	3	Consistent	MAP2K2	3	Inconsistent	RPL37	3	Inconsistent	ZC3HAV1	3	Inconsistent
SMEK2	4	Inconsistent	DNAJA4	3	Inconsistent	MARCH7	3	Inconsistent	RPS14	3	Inconsistent	ZNF358	3	Inconsistent
SNN	4	Inconsistent	DPYSL5	3	Inconsistent	MATR3	3	Inconsistent	RPS9	3	Consistent			
TCF4	4	Inconsistent	DYNLT1	3	Inconsistent	MCHR1	3	Consistent	RRBP1	3	Consistent			
THOC7	4	Inconsistent	EDEM2	3	Consistent	MED28	3	Inconsistent	RTN4R	3	Inconsistent			
TPM2	4	Inconsistent	EEF2K	3	Inconsistent	MEG3	3	Inconsistent	SEMA7A	3	Inconsistent			
TYW5	4	Inconsistent	EGFL7	3	Inconsistent	METRN	3	Inconsistent	SEPT5	3	Inconsistent			
YPEL3	4	Inconsistent	FAM8A1	3	Inconsistent	MTMR14	3	Inconsistent	SLC25A37	3	Inconsistent			
ALPL	3	Inconsistent	FBXO32	3	Inconsistent	NAGA	3	Consistent	SLC2A11	3	Consistent			
ANXA2P1	3	Consistent	FNDC4	3	Inconsistent	NDUFB2	3	Inconsistent	SNCA	3	Inconsistent			
ANXA4	3	Inconsistent	FSCN1	3	Inconsistent	NR4A1	3	Inconsistent	SNHG5	3	Consistent			
AP1B1	3	Inconsistent	GABRA5	3	Inconsistent	NRGN	3	Inconsistent	SNX19	3	Consistent			
APOPT1	3	Consistent	GATAD2A	3	Consistent	NUAK2	3	Inconsistent	SPCS1	3	Consistent			
ARL17B	3	Consistent	GJA4	3	Consistent	OAS2	3	Inconsistent	SPG7	3	Consistent			
BNIP3L	3	Inconsistent	GLG1	3	Inconsistent	PARP10	3	Inconsistent	SPHK2	3	Consistent			
C10orf54	3	Inconsistent	GLO1	3	Consistent	PARP12	3	Inconsistent	SPR	3	Consistent			
C6orf226	3	Consistent	GMFG	3	Inconsistent	PDGFRB	3	Inconsistent	SRGN	3	Consistent			
C9orf16	3	Inconsistent	GNG7	3	Consistent	PILRB	3	Inconsistent	STAU1	3	Inconsistent			
CABP1	3	Inconsistent	GPR56	3	Consistent	PINK1	3	Inconsistent	TBX2	3	Inconsistent			
CASP1	3	Consistent	H1FX-AS1	3	Inconsistent	PLD3	3	Inconsistent	TCN2	3	Consistent			
CCDC130	3	Inconsistent	HINT1	3	Inconsistent	PPP1CB	3	Consistent	TESC	3	Inconsistent			
CD151	3	Inconsistent	HIPK2	3	Inconsistent	PPP1R15A	3	Inconsistent	TGM2	3	Consistent			

**Fig. 2:**
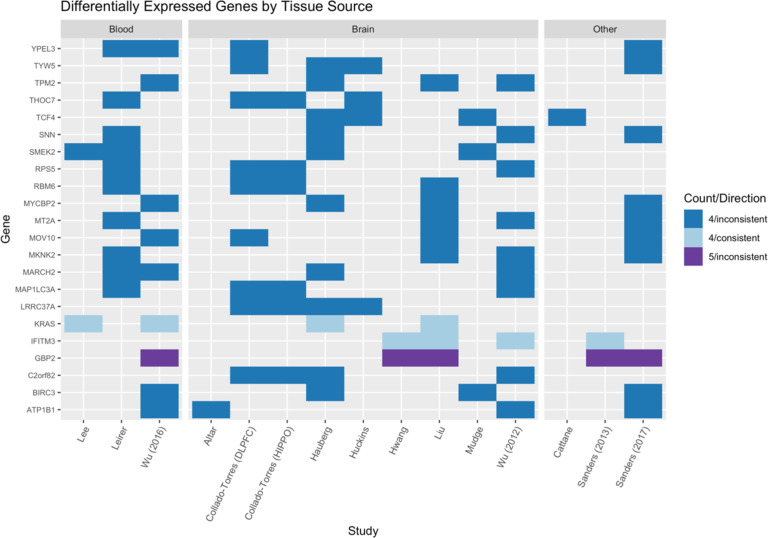
Differentially expressed genes by study and tissue source. Genes that were reported as having different expression levels in individuals with and without schizophrenia in four (blue) or five (purple) studies are shown, with the lighter color representing consistent direction of effect and the darker color representing inconsistent directions of effect across the indicated studies. The first panel shows the studies that measured expression in blood samples, the middle panel shows studies in which expression was measured in brain tissue, and the third panel shows studies completed in other tissue types (skin fibroblasts, lymphoblastoid cell lines).

Of the top 160 genes, the majority of replicated findings were inconsistent in their reported direction of effect (*n* = 108 genes). This finding did not appear to follow a pattern based on the origin tissue (as shown in Fig. 2 and Supplementary Fig. 1) or the expression measurement technology employed (Supplementary Fig. 2). The GBP2 gene, which appeared in five studies, was reported to be upregulated in individuals with schizophrenia in four studies and downregulated in one. Of the 21 genes reported as significant by four studies, 19 had inconsistent directions of effect. Of the 138 genes that appeared in three studies, 88 had the inconsistent direction of effect. Of the 50 that had a consistent direction of effect across studies, most genes (*n* = 28) were upregulated.

The finding of an association of gene expression of the GBP2 gene in five studies is greater than expected by chance. Assuming independence between the 21 sets of results reported in the 20 articles, in the absence of any true associations, the probability under a binomial model of three or more studies reporting the same transcript as significant would be 0.0012 for a permissive significance threshold of 0.01, and 1.3 × 10^-6^ at a stricter *P* value of 0.001. Given testing of ~20,000 genes, this results in an expectation of 24 or <1 such transcript significant by chance at the respective thresholds. The probability of four or more studies reporting the same transcript at a significance threshold of 0.001 is 5.9 × 10^−9^. Collectively, more genes are reported by multiple studies than would be expected by chance assuming no association.

### Overlap with previous research

Of the genes that were found in three or more studies, six have been previously reported in the largest schizophrenia case/control GWAS to date: [25] BNIP3L, CD47, CHRNA2, GATAD2A, TCF4, and TYW5. Eight genes reported as differentially expressed by three or more studies were in regions that overlap with schizophrenia-associated CNVs. PPP1R2 is in the 3q29 deletion region; HSPB1 is in the 7q11.23 duplication region; two genes (INO80E and YPEL3) are in the 16p11.2 duplication region; DHRS11 is in 17q12 deletion region; and three genes (SEPT5, RTN4R, SLC2A11) are in the 22q11 deletion region. Generally, the genes in duplication regions were reported as upregulated, and genes in deletion regions were downregulated. The exception is YPEL3, which was more often downregulated in the studies reviewed here despite being in the 16p11.2 duplication region.

Of the 160 genes reported as significantly differentially expressed in three or more studies, none showed associations with rare variants in the SCHEMA data after correction for multiple comparisons. However, six genes had rare variants nominally associated with schizophrenia with meta-analysis *P* values <0.05 (ATP1B1, CDKN1A, FAM8A1, FBXO32, PDGFRB, SPHK2), while only one, SPHK2, demonstrated a meta-analysis p-value less than 0.01 (*P* = 0.00801). There were five protein-truncating or putatively loss-of-function variants (PTVs): ATP1B1, CDKN1A, FAM8A1, FBXO32, and SPHK2; and three damaging missense variants: CDKN1A, PDGFRB, and SPHK2.

**Fig. 3:**
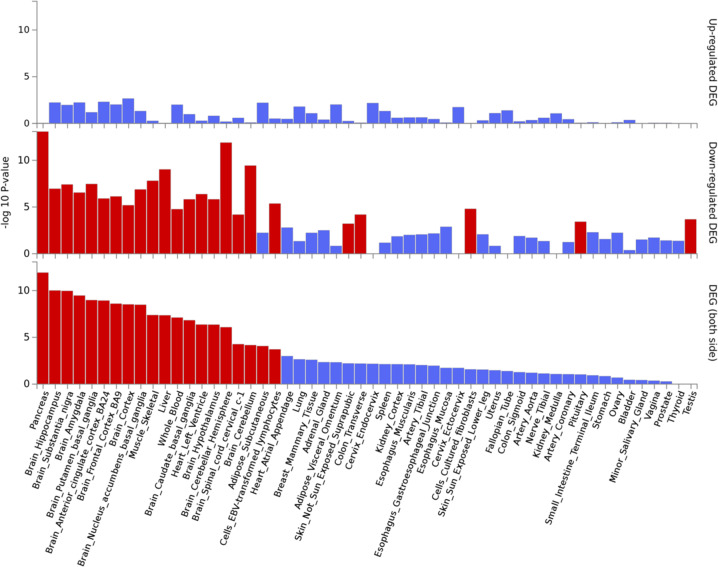
Schizophrenia gene expression tissue specificity. The -log 10 *P* value is provided for each of the 54 tissue types of the Genotype-Tissue Expression (GTEx) project v8 data, indicating whether the list of 160 genes reported as differentially expressed in individuals with schizophrenia is enriched for genes expressed in that tissue. Tissues with significant enrichment at a Bonferroni corrected *P* value < 0.05 are shown in red. The top panel shows upregulated differentially expressed genes (DEG), the middle panel shows downregulated DEG, and the bottom panel shows DEG in both directions.

Figure 3 shows the collective expression patterns of all 160 genes in 54 different tissue types using GTEx v8 data. Tissues with significant enrichment at Bonferroni corrected *P* value ≤0.05 are shown in red. This correction is completed separately for up-, down-, and both-sided sets. Here, we can see that when considering both up- and downregulated genes, our list of genes reported in schizophrenia gene expression studies is shown to be enriched for genes differentially expressed in the brain as compared to other tissues. The list is also enriched for genes differentially expressed in the pancreas, subcutaneous adipose tissue, whole blood, liver, and lymphocytes.

## Discussion

This review summarizes the literature on gene expression in schizophrenia and demonstrates the surprisingly small overlap in the genes reported across studies. Only 26 studies met our a priori inclusion criteria and were described here. The results of this review were unexpected, in that few genes were found in more than three studies, and the reported direction of effect was so variable. It was hoped that gene expression would help to explain the large number of genome-wide associated variants that are not found in genes and are theorized to be regulatory. With some exceptions described below, gene expression does not implicate the same genes that have been found by GWAS, CNV studies, or exome studies via SCHEMA. Yet, we did find that some of these top genes recapitulated findings from other types of studies in schizophrenia, as described below. Given the limited overlap with previous work, it appears that gene expression is providing us with new and different information about schizophrenia rather than bolstering previous work. This highlights the importance of the standardization of additional studies to clarify the associations between gene expression and schizophrenia.

The gene that was reported most often as differentially expressed in case–control studies of schizophrenia, GBP2, was only reported in five studies and was shown to be upregulated in four studies and downregulated in one. GBP2, guanylate-binding protein 2, is in the guanine-binding protein family, and is involved in interferon-gamma signaling and the innate immune system [55]. GBP2 has been associated with migraine [56], breast [57], and other cancers [58, 59], and HIV-1 infection [60], but has not been previously associated with psychiatric or brain/behavior phenotypes. The recent finding implicating GBP2 as a potential prognostic biomarker for pancreatic adenocarcinoma [58], could provide a model for future inquiry into its role in schizophrenia. Guanylate-binding proteins (GBPs) more broadly have been implicated in the body’s defense against infections, specifically viruses [58], and the major histocompatibility complex (MHC) has been implicated in GWAS of schizophrenia [61]. Based on maternal infection as a well-established risk factor for schizophrenia [62], a potential mechanism for the role of GBP2 in schizophrenia could be its role in reactivity to viral exposures, perhaps through epigenetic effects. This suggests that investigation of immune-related phenotypes may be useful.

Two genes that were found in four studies to have a differential expression with a consistent direction of effect, Interferon-induced transmembrane protein 3 (IFITM3) and KRAS Proto-Oncogene, GTPase (KRAS), involve immune-related function. IFITM3 has been implicated in immune function, specifically in viral restriction [63], and has been associated with cortical immune activation and inflammation, as well as neurodevelopment [64–66]. KRAS codes for K-Ras which is involved in cell proliferation, maturation, and differentiation [67]. KRAS is also involved in signal transduction and has been associated with a host of cancers [68–70] and Noonan Syndrome [71].

Several of the genes that were found to be differentially expressed in our review have also been found in the largest GWAS of schizophrenia: BNIP3L, CD47, CHRNA2, GATAD2A, TCF4, and TYW5, and have also been associated with other conditions. For example, all of these genes except TYW5 have been associated with various cancers [72–77], BNIP3L and CD47 have been associated with immune function [78] or infection [79], and CHRNA2 and TCF4 have been associated with other neuropsychiatric conditions and substance use [80, 81]. GATAD2A has been associated with cardiometabolic disease and type 2 diabetes [82, 83], which is pertinent because of the increased risk of metabolic syndrome secondary to the use of atypical antipsychotic medications in patients with schizophrenia [84].

The CNV findings in this study also may have important etiologic relevance for both schizophrenia as well as its core features and associations with other conditions. Five schizophrenia-associated CNV regions contained genes that were found to be differentially expressed in this study: PPP1R2 in 3q29, HSPB1 in 7q11.23, INO80E and YPEL3 in 16p11.2, DHRS11 in 17q12, and SEPT5, RTN4R, and SLC2A11 in 22q11.2. Though these CNVs have wide-ranging phenotypic presentations, CNVs in these regions are all associated with developmental delays and intellectual disability. They are also associated with neuropsychiatric phenotypes in addition to schizophrenia, notably anxiety (3q29, 7q11.23, 17q12), autism spectrum disorder (ASD; 3q29, 7q11.23, 16p11.2, 17q12, 22q), attention-deficit/hyperactivity disorder (ADHD; 7q11.23, 22q), and bipolar disorder (3q29, 7q11.23, 17q12). The physical manifestations of these CNVs also share some commonalities: 3q29, 7q11.23, 16p11.2 and 22q are all associated with cardiac problems and varying craniofacial dysmorphology, while 17q12 and 22q are both associated with immune system dysfunction. Studies have examined some of these comorbid conditions explicitly such as the demonstration of pleiotropic effects of the INO80E gene in 16p11.2 on both schizophrenia and cardiometabolic disease [82]. The most well-known CNV associated with an increased risk of schizophrenia is the 22q deletion syndrome, where ~25% of cases go on to develop schizophrenia [85]. The 22q deletion syndrome is one of the strongest risk factors for schizophrenia because of its high prevalence, thereby warranting emphasis in future studies [86].

After querying the 160 differentially expressed genes in SCHEMA, only one gene, SPHK2, showed suggestive evidence of association in the SCHEMA database at *P* < 0.01. The associated variant is classified in the database as a protein-truncating variant (PTV; stop-gained, frameshift, and essential splice donor or acceptor variants), which suggests that the effect on schizophrenia most likely tracks with decreasing expression of the gene [87]. SPHK2 mediates cellular migration, proliferation, and apoptosis, and may be responsible for promoting angiogenesis and tumorigenesis in some cancers. Multiple isoforms of SPHK2 have been observed from alternatively spliced transcript variants [88].

As we would expect, the genes found in this study appear to be differentially expressed in the brain when compared to other body tissues. However, there are some unexpected results; notably, differential expression in the pancreas, subcutaneous adipose tissue, whole blood, liver, and lymphocytes. It is possible that the differential expression in blood, brain, and lymphocytes is due in some part to these being the tissues assayed in the transcriptome abundance studies summarized here. However, the finding of differential expression in the pancreas, adipose tissue, and liver is interesting given that metabolic syndrome is often associated with schizophrenia [84]. It would be valuable to investigate whether this effect was secondary to medication, but only one of the included studies covaried for medication [89]. Although several studies have used FUMA [28] to annotate genetic variants with functional information from GWAS summary statistics (e.g., Liu et al. [82] and Wendt et al. [90]) we did not find that others had used the GENE2FUNC module to examine tissue expression in schizophrenia.

### Recommendations for future research

#### Greater consensus on standard formats for reporting data and results

One of the greatest challenges in completing the work presented in this review was the inconsistency in the way the results of gene expression studies were reported. A great deal of effort was devoted to convert the results to a standard nomenclature before the data could be aggregated for analysis. Different statistical significance thresholds and corrections for multiple comparisons were applied, and at times the number of transcripts evaluated was not reported such that it could not be determined if the correction for multiple comparisons was appropriate. These manuscripts were published over several years, and there has not yet been a coalescence of standards of reporting in the field. Efforts to standardize methods and reporting, similar to those for GWAS [91], would help to enhance the interpretation and discernment of common findings in future studies. It would also be beneficial for the field to adopt standard naming conventions and a well-defined statistical significance threshold for the transcriptome.

#### Better definition of covariates

There are several factors that may impact gene expression [16] that should be included as covariate in gene expression studies, notably, age, sex, race, or genetic ancestry principal components, medication usage, smoking status, and batch. In addition, for the postmortem brain studies, the cause of death and postmortem interval may be relevant. Few of the studies reviewed considered all of these factors (as shown in Supplementary Table 2). Only one study [92] considered medication use and smoking status as covariates. Smoking status is an important covariate in the present context given that patients with schizophrenia are more likely to smoke than are healthy controls (>60% versus ~17%) [93], and gene expression is known to differ between those who smoke and those who do not [94]. When the top 160 genes from this study were compared to the 1270 genes differentially expressed between current and never smokers [95], 24 were common to both studies, including GBP2 (Supplementary Table 3). This may mean that had these studies controlled for smoking status, the results presented, and conclusions drawn from the work may have been different.

#### Case/control definition

The definition of cases ranged from a statement that the participant population included “schizophrenia cases” to more stringent definitions based on multiple clinician assessments of ICD or DSM criteria. The selection of the control samples was also highly variable and often not specified. Many of the studies did not state the source of controls, nor whether they were screened or interviewed to rule out schizophrenia or another major mental disorder. In future meta-analytic studies, it would also be interesting to examine longitudinal changes in gene expression between the first episode and long-standing schizophrenia (as in Ota et al. [96]), as well as the specificity of the findings with respect to schizophrenia versus other mental disorders such as bipolar disorder.

An additional consideration in examining this body of literature is the challenge of differentiating whether the participants were included in more than one study, especially in the research utilizing postmortem brain tissue. For example, at least two studies [43, 97] used postmortem brain tissue from the CommonMind consortium [98]. There are several other brain banks that are commonly used, for example, the Maryland Brain Collection [99], the Stanley Neuropathology Consortium (SNC [92]), and the Stanley Medical Research Institute (SMRI [100]). Though there does not appear to be sample overlap in the studies reviewed here, it is likely that other studies do use these data.

#### Expression technology

Some of the variability in the results reported here may be the result of the use of different gene expression measurement technologies. Of course, some of these will change over time, but standardization of RNA-seq data processing pipelines (for the studies that use this technology) may lead to a greater coalescence of the reported results [101]. In addition, it has been reported that there is bias in the publication of gene expression studies; notably, that genes are more likely to appear in the literature if they are over-expressed, found to be associated in multiple diseases, and are more “popular” in the biomedical literature [9]. Moreover, one might expect greater overlap in the studies that used imputed data, as this approach implicitly controls for environmental sources of variation. However, there was a lack of synergy across the studies that used imputed gene expression.

#### Standardized reporting of pathways

The pathway analytic results reported in the studies reviewed here were not synthesized due to the number of different approaches employed. Although articles such as Bousman et al. [47] and Wei et al. [49] identified pathways that were differentially expressed in patients with schizophrenia, their use of different pathway nomenclature made comparisons across these studies difficult. Unfortunately, Gene Ontology (http://geneontology.org/), Ingenuity (www.ingenuity.com), and KEGG (https://www.genome.jp/kegg/) pathway analyses were completed and the results from each of these are not directly comparable to one another [102]. In addition, the lack of open-source programs that can convert between gene pathway nomenclature made it nearly impossible to standardize data from multiple authors. Of course, the differences between KEGG, Ingenuity, Gene Ontology, and Reactome are not purely ones of nomenclature but also due to the different data underlying pathway definitions, e.g., whether they are based on biological pathways, ontological gene-set pathways, or statistically associated entities. Therefore, the pathway definitions will subtly differ across these databases, so reporting the results from multiple pathway approaches in the same manuscript may be a better approach to facilitating comparisons across studies. Researchers could also include information about which genes in the pathway have the strongest signal, potentially allowing for gene-level comparisons in ancillary work derived from pathway analysis. Nomenclature standardization and reporting results across techniques would allow larger meta-analyses to occur and offer a clearer picture of the differentially expressed gene pathways in patients with schizophrenia.

#### Public repositories

One area for future consideration for schizophrenia gene expression reporting is the lack of publicly available results repositories. Given the wide variety of GWAS repositories (e.g., the database of Genotypes and Phenotypes (dbGaP), https://www.ncbi.nlm.nih.gov/gap/; the NHGRI-EBI GWAS Catalog, https://www.ebi.ac.uk/gwas/), it was interesting to note that transcriptome-wide association study (TWAS) repositories such as the Gene Expression database of Normal and Tumor tissues (GENT2, http://gent2.appex.kr/gent2/) and the Gene Expression Omnibus (GEO, https://www.ncbi.nlm.nih.gov/geo/) contained solely experimental data but not results. The creation of publicly shared gene expression result repositories would facilitate widespread sharing of information between lab groups and allow for much easier meta-analyses. Similarly, such a repository would help lead to a standardized format of reporting results.

### Strengths and limitations

The main strengths of this work are our exhaustive search of multiple databases, and interpretation of the replicated results across different types of genetic associations (i.e., CNVs, GWAS, exome sequencing). Additional strengths include the examination of concordance of the results across tissues and assay methods. The primary limitation is that we were unable to complete a meta-analysis of gene expression in schizophrenia due to differences in the study methodology (i.e., selection of cases, convenience versus selected controls), analytic methods (i.e., quantification of case–control differences, control for multiple testing, inclusion of confounders, statistical significance thresholds), and gene expression methodology (i.e., gene nomenclature, measures of gene expression, different tissue types, etc.).

## Conclusion

To our knowledge, this is the first systematic review and synthesis of controlled studies of gene expression in schizophrenia. This work adds to the body of knowledge that demonstrates differential gene expression between schizophrenia cases and controls. Although case–control differences were reported in numerous studies, only one gene, GBP2, was replicated in five studies. This suggests that further inquiry into the potential role of GBP2 could have promise in advancing our understanding of the genetic architecture of schizophrenia. These findings also highlight the methodological inconsistencies that impede valid meta-analyses and synthesis across studies. Standardization of the use of covariates, gene nomenclature, and methods for reporting results could enhance our understanding of the potential mechanisms through which genes exert their influence on the etiology of schizophrenia.

## Supplementary information

What Genes are Differentially Expressed in Individuals with Schizophrenia? A systematic review. Supplementary information.

Supplementary table 1: 6771 unique genes reported as statistically significantly differentially expressed in schizophrenia cases

Supplementary table 4: PRSIMA 2020 Checklist
