# Supplementary material for: What genes are differentially expressed in individuals with schizophrenia? A systematic review
Source: Mol Psychiatry. 2022 Jan 28;27(3):1373–83. doi: 10.1038/s41380-021-01420-7 (PMC9095490; doi:10.1038/s41380-021-01420-7)
Supplement: Supplementary file 1 — What Genes are Differentially Expressed in Individuals with Schizophrenia? A systematic review. Supplementary information. [file 41380_2021_1420_MOESM1_ESM.docx]

Title: What Genes are Differentially Expressed in Individuals with Schizophrenia? A systematic review.

# Supplementary information

## Literature search information

In our effort to find all of the studies that had investigated differential expression of genes in schizophrenia, we searched PubMed, EMBASE, and Web of Science electronic bibliographic databases with the following search terms:

PubMed:

"Schizophrenia"[MeSH] AND ("Transcriptome"[MeSH] OR "Transcription"[MeSH] OR "Sequence Analysis, RNA"[MeSH] OR "Microarray Analysis"[MeSH]) AND "humans"[MeSH Terms] AND "case-control studies"[MeSH Terms]) AND "humans"[MeSH Terms]

EmBase:

('schizophrenia'/exp OR schizophrenia) AND ('transcriptome'/exp OR transcriptome OR 'microarray analysis'/exp OR 'microarray analysis' OR 'rna sequence'/exp OR 'rna sequence' OR 'gene expression'/exp OR 'gene expression') AND ('case control study'/exp OR 'case control study') AND 'human'/de AND 'article'/it

Web of Science:

TS=(Schizophr* AND (Transcriptome OR Transcription OR "Microarray Analysis" OR "RNA sequence") )) AND LANGUAGE: (English) AND DOCUMENT TYPES: (Article)

Refined by: TOPIC: (case control)

Timespan: 2000-2020

We included case-control studies comparing the transcriptome of individuals diagnosed with schizophrenia (using any recognized standard diagnostic criteria) to healthy controls. Intervention studies were excluded. Only human studies were included, and animal studies were excluded. Studies were limited to English language publications. We considered all measures of effect, which were typically regression beta coefficients or ratio measures (e.g., fold change in expression between cases and controls). No other outcomes were considered.

## Data extraction and coding

Data was extracted using a standard coding form for the studies that met inclusion criteria. Extracted information included:

Sample size, including the number of cases and controls

Diagnosis

Type of tissue used

Methodology (how gene expression was measured)

Covariates

Age range

% male and female

Major findings

It was required that gene nomenclature was standardized in order to compare results across studies. The majority of studies reported their results using HUGO gene symbols, so studies that used alternative nomenclature (i.e., gene name, Ensembl ID) were converted to HUGO symbols via Bioconductor R Packages, the DAVID Gene ID Conversion Tool (https://david.abcc.ncifcrf.gov/conversion.jsp), or BioMart (<http://www.biomart.org/>).

Data collection forms and analytic code are available upon request from the corresponding author.

**Supplementary table 1:** **6771 unique genes reported as statistically significantly differentially expressed in schizophrenia cases**

The list of genes found to be statistically significantly differentially expressed in any of the studies reviewed here is shown in supplementary table 1.

**Supplementary table 2: Study covariates**

As noted in the main manuscript, there are several factors that may impact gene expression that should be included in gene expression studies.^1^ We recorded whether the studies reviewed considered age, sex, race, or genetic ancestry principal components, medication usage, smoking status, and batch as covariates. In addition, for the post-mortem brain studies, we recorded if the authors reported the cause of death and post-mortem interval. This information is shown in supplementary table 2.

**Supplementary table 3: Genes with differential expression reported in three or more schizophrenia gene expression studies and also in current versus never smokers**

The Huan et al.^2^ meta-analysis of transcriptome-wide gene expression using whole blood-derived RNA in six cohorts (N=10,233 European ancestry participants) identified associations between smoking and altered gene expression levels. They identified 1270 differentially expressed genes in current versus never smokers at a false discovery rate (FDR)<0.1. The overlap between those genes and the genes found in three or more studies in this review are shown in Supplementary Table 3. The meta-analytic results from Huan et al. are presented.

**Supplementary table 4:** PRSIMA 2020 Checklist

**Supplementary figure 1** is an expanded version of the expression summary by tissue plot that appears in the main manuscript. This plot includes genes that are differentially expressed in three or more studies. Genes that appear three times are shown in green, four times are shown in blue, and five times are shown in purple. A consistent direction of expression is indicated by the darker shade.

**Supplementary figure 2** shows genes that were differentially expressed in three or more studies by the technology used to quantify gene expression. As in supplementary figure 1, genes that appear three times are shown in green, four times are shown in blue, and five times are shown in purple. A consistent direction of expression is indicated by the darker shade.

**Supplementary Table 2: Study Covariates**

|  | **Author** | **Year** | **Sex/**  **gender** | **Race/**  **ethnicity** | **Ancestry**  **PCs** | **Smoking status** | **Medication**  **usage** | **Sample quality** | **Batch Effects** | **Cause of death** | **Post-mortem interval** |
| --- | --- | --- | --- | --- | --- | --- | --- | --- | --- | --- | --- |
| **Blood** | Bousman^3^ | 2010 | Yes | No | No | No | Yes | No | No | N/A | N/A |
|  | Chen^4^ | 2016 | Yes | Yes | No | No | No | No | No | N/A | N/A |
|  | Gardiner^5^ | 2012 | Yes | No | No | No | No | Yes | Yes | N/A | N/A |
|  | Kuzman^6^ | 2009 | Yes | No | No | No | No | No | No | N/A | N/A |
|  | Lee^7^ | 2012 | Yes | Yes | No | No | No | No | No | N/A | N/A |
|  | Leirer^8^ | 2019 | Yes | Yes | No | No | No | No | No | N/A | N/A |
|  | Wei^9^ | 2015 | Yes | No | No | Yes | No | No | No | N/A | N/A |
|  | Wu^10^ | 2016 | No | No | No | No | No | Yes | No | N/A | N/A |
|  | Yu^11^ | 2015 | Yes | No | No | No | No | No | No | N/A | N/A |
|  | Zhang^12^ | 2015 | No | No | No | No | No | No | No | N/A | N/A |
| **Brain** | Altar^13^ | 2005 | Yes | Yes | No | No | No | Yes | No | Yes | Yes |
|  | Collado-Torres^14^ | 2019 | Yes | Yes | Yes | No | No | Yes | No | No | Yes |
|  | Harris^15^ | 2008 | Yes | No | No | No | Yes | Yes | No | No | Yes |
|  | Hauberg^16^ | 2019 | Yes | No | Yes | No | No | Yes | Yes | No | Yes |
|  | Huckins^17^ | 2019 | No | No | Yes | No | No | Yes | Yes | No | No |
|  | Hwang^18^ | 2013 | Yes | No | No | Yes | Yes | Yes | No | No | Yes |
|  | Liu^19^ | 2018 | Yes | No | No | No | No | No | No | Yes | No |
|  | Mudge^20^ | 2008 | N/A | No | No | No | No | Yes | Yes | Yes | Yes |
|  | Schmitt^21^ | 2012 | Yes | No | Yes | No | Yes | Yes | No | No | Yes |
|  | Sellmann^22^ | 2014 | No | No | Yes | No | No | No | No | No | No |
|  | Tian^23^ | 2018 | Yes | No | No | No | No | No | No | No | Yes |
|  | Wu^24^ | 2012 | Yes | No | No | No | No | No | No | No | No |
| **Other** | Cattane^25^ | 2015 | No | No | No | No | No | No | Yes | N/A | N/A |
|  | Lin^26^ | 2016 | No | No | No | No | No | No | Yes | N/A | N/A |
|  | Sanders^27^ | 2017 | Yes | No | No | No | No | Yes | Yes | N/A | N/A |
|  | Sanders^28^ | 2013 | Yes | No | Yes | No | No | Yes | Yes | N/A | N/A |

**Supplementary Table 3: Genes with differential expression reported in three or more schizophrenia gene expression studies and also in current versus never smokers**

| **Gene Symbol** | **Entrez Gene ID** | **CHR** | **Meta Beta** | **Meta SE** | **Meta z-value** | **Meta p-value** | **Meta FDR** |
| --- | --- | --- | --- | --- | --- | --- | --- |
| ATP1B1 | 481 | 1 | -0.0219675 | 0.00610949 | -3.5956276 | 0.00032361 | 0.01365255 |
| BIRC3 | 330 | 11 | 0.03999906 | 0.00806266 | 4.96102409 | 7.01E-07 | 8.15E-05 |
| CD47 | 961 | 3 | -0.0320851 | 0.00987904 | -3.2477933 | 0.00116304 | 0.03488001 |
| FBXO32 | 114907 | 8 | 0.01405881 | 0.00489938 | 2.86950834 | 0.00411111 | 0.08686612 |
| GBP2 | 2634 | 1 | -0.0511487 | 0.01698621 | -3.011191 | 0.00260225 | 0.06320206 |
| HINT1 | 3094 | 5 | 0.09083224 | 0.02038169 | 4.45656059 | 8.33E-06 | 0.00068526 |
| HSP90AB1 | 3326 | 6 | 0.04997625 | 0.0131471 | 3.80131265 | 0.00014393 | 0.00721152 |
| JAZF1 | 221895 | 7 | -0.0671311 | 0.01829879 | -3.6686094 | 0.00024387 | 0.01087337 |
| MED28 | 80306 | 4 | 0.05044621 | 0.01103808 | 4.57019775 | 4.87E-06 | 0.00042919 |
| MT2A | 4502 | 16 | -0.0755701 | 0.02649979 | -2.8517247 | 0.00434827 | 0.09013243 |
| NRGN | 4900 | 11 | -0.1176167 | 0.03078901 | -3.8200854 | 0.00013341 | 0.00683417 |
| PTGS1 | 5742 | 9 | -0.0266525 | 0.00883439 | -3.0169071 | 0.00255368 | 0.06223544 |
| RAB3IP | 117177 | 12 | 0.02688748 | 0.00850465 | 3.16150489 | 0.00156956 | 0.04387432 |
| RPL35 | 11224 | 9 | 0.05829652 | 0.01408656 | 4.1384505 | 3.50E-05 | 0.00227978 |
| RPS14 | 6208 | 5 | 0.06889929 | 0.01056334 | 6.52249054 | 6.91E-11 | 1.97E-08 |
| RPS5 | 6193 | 19 | 0.06458133 | 0.01575902 | 4.09805487 | 4.17E-05 | 0.00266117 |
| RPS9 | 6203 | 19 | 0.05075256 | 0.01230118 | 4.12582913 | 3.69E-05 | 0.00238373 |
| SLC25A37 | 51312 | 8 | -0.0907374 | 0.02134356 | -4.2512764 | 2.13E-05 | 0.001478 |
| SNCA | 6622 | 4 | -0.1110488 | 0.02429895 | -4.5701077 | 4.87E-06 | 0.00042919 |
| SNHG5 | 387066 | 6 | 0.07924249 | 0.02359984 | 3.35775501 | 0.00078578 | 0.02672724 |
| TMEM204 | 79652 | 16 | 0.08906825 | 0.01687173 | 5.27914029 | 1.30E-07 | 1.88E-05 |
| TPM2 | 7169 | 9 | 0.01713145 | 0.00581416 | 2.94650769 | 0.00321384 | 0.07320682 |
| UQCRH | 7388 | 1 | 0.03837091 | 0.01219087 | 3.14751286 | 0.00164666 | 0.04537259 |
| ZNF358 | 140467 | 19 | 0.04261642 | 0.01365527 | 3.12087748 | 0.00180313 | 0.04830542 |

CHR=Chromosome; FDR=False discovery rate; SE=Standard error


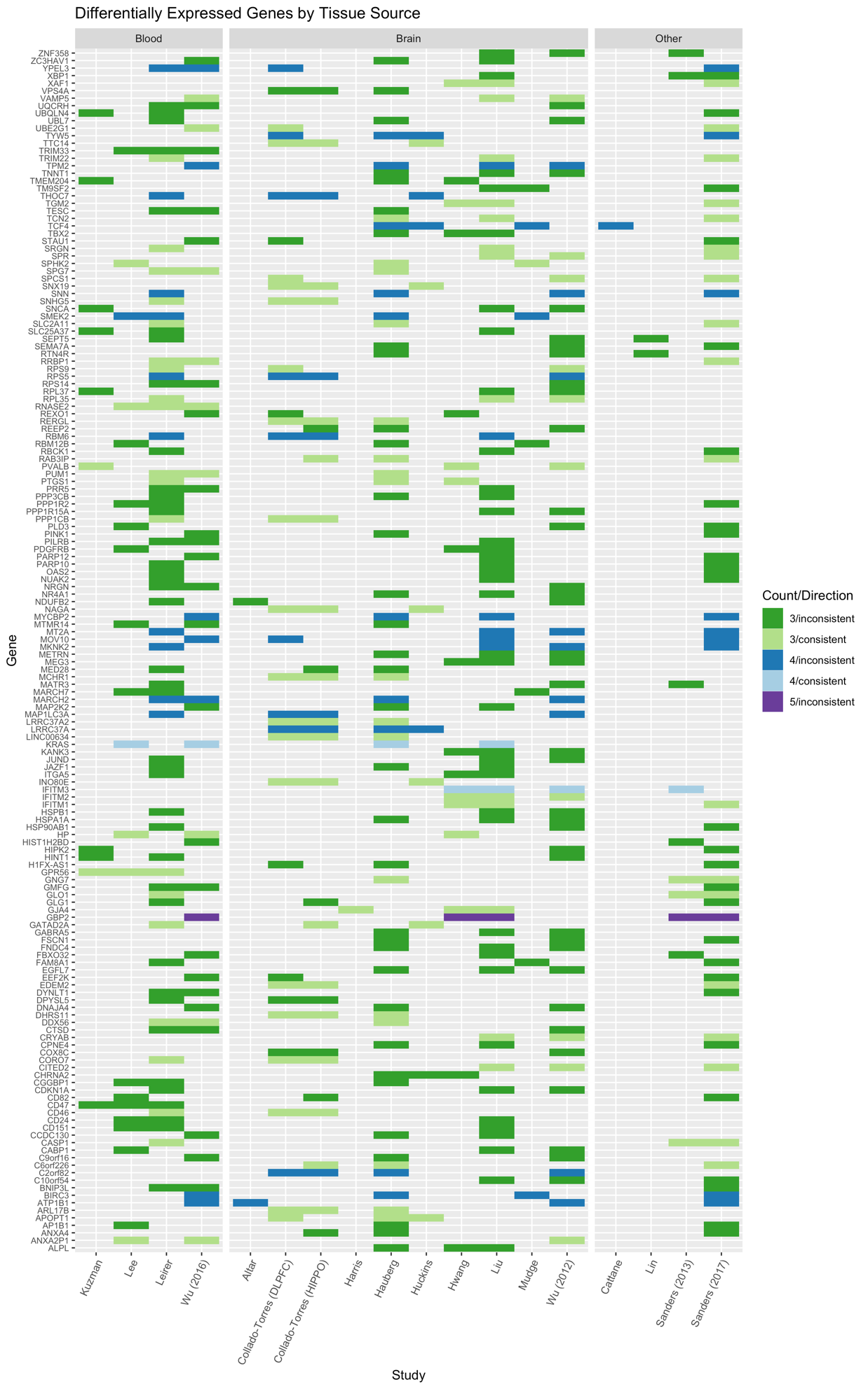


Supplementary Figure 1: Differentially expressed genes by tissue source


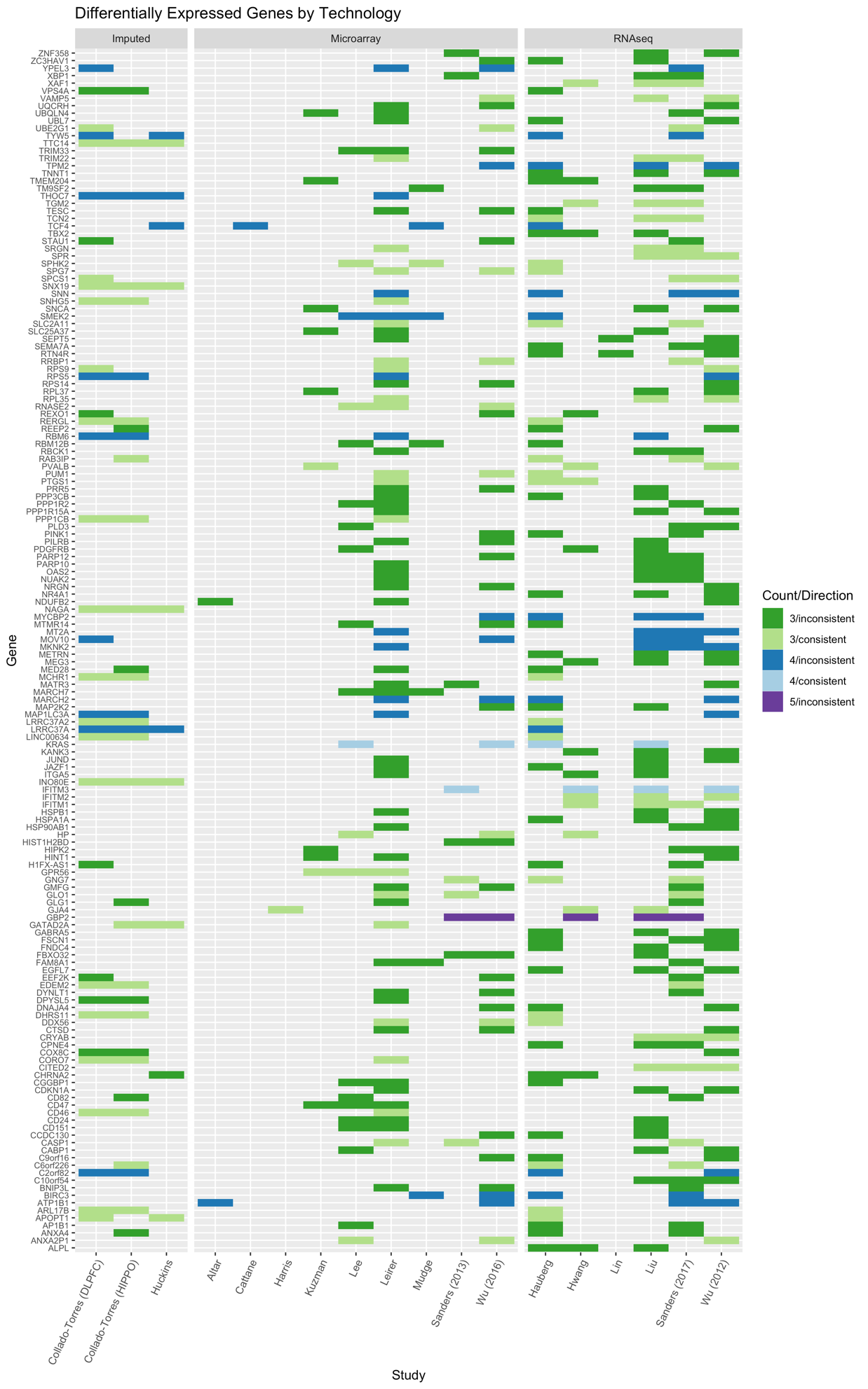


Supplementary Figure 2: Differentially expressed genes by the technology used to quantify gene expression

References

1. Mele M, Ferreira PG, Reverter F, DeLuca DS, Monlong J, Sammeth M *et al.* Human genomics. The human transcriptome across tissues and individuals. *Science* 2015; **348**(6235)**:** 660-665.

2. Huan T, Joehanes R, Schurmann C, Schramm K, Pilling LC, Peters MJ *et al.* A whole-blood transcriptome meta-analysis identifies gene expression signatures of cigarette smoking. *Hum Mol Genet* 2016; **25**(21)**:** 4611-4623.

3. Bousman CA, Chana G, Glatt SJ, Chandler SD, Lucero GR, Tatro E *et al.* Preliminary evidence of ubiquitin proteasome system dysregulation in schizophrenia and bipolar disorder: convergent pathway analysis findings from two independent samples. *Am J Med Genet B Neuropsychiatr Genet* 2010; **153B**(2)**:** 494-502.

4. Chen S, Sun X, Niu W, Kong L, He M, Li W *et al.* Aberrant Expression of Long Non-Coding RNAs in Schizophrenia Patients. *Med Sci Monit* 2016; **22:** 3340-3351.

5. Gardiner E, Beveridge NJ, Wu JQ, Carr V, Scott RJ, Tooney PA *et al.* Imprinted DLK1-DIO3 region of 14q32 defines a schizophrenia-associated miRNA signature in peripheral blood mononuclear cells. *Mol Psychiatry* 2012; **17**(8)**:** 827-840.

6. Kuzman MR, Medved V, Terzic J, Krainc D. Genome-wide expression analysis of peripheral blood identifies candidate biomarkers for schizophrenia. *J Psychiatr Res* 2009; **43**(13)**:** 1073-1077.

7. Lee J, Goh LK, Chen G, Verma S, Tan CH, Lee TS. Analysis of blood-based gene expression signature in first-episode psychosis. *Psychiatry Res* 2012; **200**(1)**:** 52-54.

8. Leirer DJ, Iyegbe CO, Di Forti M, Patel H, Carra E, Fraietta S *et al.* Differential gene expression analysis in blood of first episode psychosis patients. *Schizophr Res* 2019; **209:** 88-97.

9. Wei H, Yuan Y, Liu S, Wang C, Yang F, Lu Z *et al.* Detection of circulating miRNA levels in schizophrenia. *Am J Psychiatry* 2015; **172**(11)**:** 1141-1147.

10. Wu JQ, Green MJ, Gardiner EJ, Tooney PA, Scott RJ, Carr VJ *et al.* Altered neural signaling and immune pathways in peripheral blood mononuclear cells of schizophrenia patients with cognitive impairment: A transcriptome analysis. *Brain Behav Immun* 2016; **53:** 194-206.

11. Yu HC, Wu J, Zhang HX, Zhang GL, Sui J, Tong WW *et al.* Alterations of miR-132 are novel diagnostic biomarkers in peripheral blood of schizophrenia patients. *Prog Neuropsychopharmacol Biol Psychiatry* 2015; **63:** 23-29.

12. Zhang F, Xu Y, Shugart YY, Yue W, Qi G, Yuan G *et al.* Converging evidence implicates the abnormal microRNA system in schizophrenia. *Schizophr Bull* 2015; **41**(3)**:** 728-735.

13. Altar CA, Jurata LW, Charles V, Lemire A, Liu P, Bukhman Y *et al.* Deficient hippocampal neuron expression of proteasome, ubiquitin, and mitochondrial genes in multiple schizophrenia cohorts. *Biol Psychiatry* 2005; **58**(2)**:** 85-96.

14. Collado-Torres L, Burke EE, Peterson A, Shin J, Straub RE, Rajpurohit A *et al.* Regional Heterogeneity in Gene Expression, Regulation, and Coherence in the Frontal Cortex and Hippocampus across Development and Schizophrenia. *Neuron* 2019; **103**(2)**:** 203-216 e208.

15. Harris LW, Wayland M, Lan M, Ryan M, Giger T, Lockstone H *et al.* The cerebral microvasculature in schizophrenia: a laser capture microdissection study. *PLoS One* 2008; **3**(12)**:** e3964.

16. Hauberg ME, Fullard JF, Zhu L, Cohain AT, Giambartolomei C, Misir R *et al.* Differential activity of transcribed enhancers in the prefrontal cortex of 537 cases with schizophrenia and controls. *Mol Psychiatry* 2019; **24**(11)**:** 1685-1695.

17. Huckins LM, Dobbyn A, Ruderfer DM, Hoffman G, Wang W, Pardinas AF *et al.* Gene expression imputation across multiple brain regions provides insights into schizophrenia risk. *Nat Genet* 2019; **51**(4)**:** 659-674.

18. Hwang Y, Kim J, Shin JY, Kim JI, Seo JS, Webster MJ *et al.* Gene expression profiling by mRNA sequencing reveals increased expression of immune/inflammation-related genes in the hippocampus of individuals with schizophrenia. *Transl Psychiatry* 2013; **3:** e321.

19. Liu Y, Chang X, Hahn CG, Gur RE, Sleiman PAM, Hakonarson H. Non-coding RNA dysregulation in the amygdala region of schizophrenia patients contributes to the pathogenesis of the disease. *Transl Psychiatry* 2018; **8**(1)**:** 44.

20. Mudge J, Miller NA, Khrebtukova I, Lindquist IE, May GD, Huntley JJ *et al.* Genomic convergence analysis of schizophrenia: mRNA sequencing reveals altered synaptic vesicular transport in post-mortem cerebellum. *PLoS One* 2008; **3**(11)**:** e3625.

21. Schmitt A, Leonardi-Essmann F, Durrenberger PF, Wichert SP, Spanagel R, Arzberger T *et al.* Structural synaptic elements are differentially regulated in superior temporal cortex of schizophrenia patients. *Eur Arch Psychiatry Clin Neurosci* 2012; **262**(7)**:** 565-577.

22. Sellmann C, Villarin Pildain L, Schmitt A, Leonardi-Essmann F, Durrenberger PF, Spanagel R *et al.* Gene expression in superior temporal cortex of schizophrenia patients. *Eur Arch Psychiatry Clin Neurosci* 2014; **264**(4)**:** 297-309.

23. Tian T, Wei Z, Chang X, Liu Y, Gur RE, Sleiman PMA *et al.* The Long Noncoding RNA Landscape in Amygdala Tissues from Schizophrenia Patients. *EBioMedicine* 2018; **34:** 171-181.

24. Wu JQ, Wang X, Beveridge NJ, Tooney PA, Scott RJ, Carr VJ *et al.* Transcriptome sequencing revealed significant alteration of cortical promoter usage and splicing in schizophrenia. *PLoS One* 2012; **7**(4)**:** e36351.

25. Cattane N, Minelli A, Milanesi E, Maj C, Bignotti S, Bortolomasi M *et al.* Altered gene expression in schizophrenia: findings from transcriptional signatures in fibroblasts and blood. *PLoS One* 2015; **10**(2)**:** e0116686.

26. Lin M, Pedrosa E, Hrabovsky A, Chen J, Puliafito BR, Gilbert SR *et al.* Integrative transcriptome network analysis of iPSC-derived neurons from schizophrenia and schizoaffective disorder patients with 22q11.2 deletion. *BMC Syst Biol* 2016; **10**(1)**:** 105.

27. Sanders AR, Drigalenko EI, Duan J, Moy W, Freda J, Goring HHH *et al.* Transcriptome sequencing study implicates immune-related genes differentially expressed in schizophrenia: new data and a meta-analysis. *Transl Psychiatry* 2017; **7**(4)**:** e1093.

28. Sanders AR, Goring HH, Duan J, Drigalenko EI, Moy W, Freda J *et al.* Transcriptome study of differential expression in schizophrenia. *Hum Mol Genet* 2013; **22**(24)**:** 5001-5014.
