# Supplementary material for: What genes are differentially expressed in individuals with schizophrenia? A systematic review
Source: Mol Psychiatry. 2022 Jan 28;27(3):1373–83. doi: 10.1038/s41380-021-01420-7 (PMC9095490; doi:10.1038/s41380-021-01420-7)
Supplement: Supplementary file 2 — Supplementary table 1: 6771 unique genes reported as statistically significantly differentially expressed in schizophrenia cases [file 41380_2021_1420_MOESM2_ESM.docx]

| **Gene** | **Count** |
| --- | --- |
| GBP2 | 5 |
| ATP1B1 | 4 |
| BIRC3 | 4 |
| C2orf82 | 4 |
| IFITM3 | 4 |
| KRAS | 4 |
| LRRC37A | 4 |
| MAP1LC3A | 4 |
| MARCH2 | 4 |
| MKNK2 | 4 |
| MOV10 | 4 |
| MT2A | 4 |
| MYCBP2 | 4 |
| RBM6 | 4 |
| RPS5 | 4 |
| SMEK2 | 4 |
| SNN | 4 |
| TCF4 | 4 |
| THOC7 | 4 |
| TPM2 | 4 |
| TYW5 | 4 |
| YPEL3 | 4 |
| ALPL | 3 |
| ANXA2P1 | 3 |
| ANXA4 | 3 |
| AP1B1 | 3 |
| APOPT1 | 3 |
| ARL17B | 3 |
| BNIP3L | 3 |
| C10orf54 | 3 |
| C6orf226 | 3 |
| C9orf16 | 3 |
| CABP1 | 3 |
| CASP1 | 3 |
| CCDC130 | 3 |
| CD151 | 3 |
| CD24 | 3 |
| CD46 | 3 |
| CD47 | 3 |
| CD82 | 3 |
| CDKN1A | 3 |
| CGGBP1 | 3 |
| CHRNA2 | 3 |
| CITED2 | 3 |
| CORO7 | 3 |
| COX8C | 3 |
| CPNE4 | 3 |
| CRYAB | 3 |
| CTSD | 3 |
| DDX56 | 3 |
| DHRS11 | 3 |
| DNAJA4 | 3 |
| DPYSL5 | 3 |
| DYNLT1 | 3 |
| EDEM2 | 3 |
| EEF2K | 3 |
| EGFL7 | 3 |
| FAM8A1 | 3 |
| FBXO32 | 3 |
| FNDC4 | 3 |
| FSCN1 | 3 |
| GABRA5 | 3 |
| GATAD2A | 3 |
| GJA4 | 3 |
| GLG1 | 3 |
| GLO1 | 3 |
| GMFG | 3 |
| GNG7 | 3 |
| GPR56 | 3 |
| H1FX-AS1 | 3 |
| HINT1 | 3 |
| HIPK2 | 3 |
| HIST1H2BD | 3 |
| HP | 3 |
| HSP90AB1 | 3 |
| HSPA1A | 3 |
| HSPB1 | 3 |
| IFITM1 | 3 |
| IFITM2 | 3 |
| INO80E | 3 |
| ITGA5 | 3 |
| JAZF1 | 3 |
| JUND | 3 |
| KANK3 | 3 |
| LINC00634 | 3 |
| LRRC37A2 | 3 |
| MAP2K2 | 3 |
| MARCH7 | 3 |
| MATR3 | 3 |
| MCHR1 | 3 |
| MED28 | 3 |
| MEG3 | 3 |
| METRN | 3 |
| MTMR14 | 3 |
| NAGA | 3 |
| NDUFB2 | 3 |
| NR4A1 | 3 |
| NRGN | 3 |
| NUAK2 | 3 |
| OAS2 | 3 |
| PARP10 | 3 |
| PARP12 | 3 |
| PDGFRB | 3 |
| PILRB | 3 |
| PINK1 | 3 |
| PLD3 | 3 |
| PPP1CB | 3 |
| PPP1R15A | 3 |
| PPP1R2 | 3 |
| PPP3CB | 3 |
| PRR5 | 3 |
| PTGS1 | 3 |
| PUM1 | 3 |
| PVALB | 3 |
| RAB3IP | 3 |
| RBCK1 | 3 |
| RBM12B | 3 |
| REEP2 | 3 |
| RERGL | 3 |
| REXO1 | 3 |
| RNASE2 | 3 |
| RPL35 | 3 |
| RPL37 | 3 |
| RPS14 | 3 |
| RPS9 | 3 |
| RRBP1 | 3 |
| RTN4R | 3 |
| SEMA7A | 3 |
| SEPT5 | 3 |
| SLC25A37 | 3 |
| SLC2A11 | 3 |
| SNCA | 3 |
| SNHG5 | 3 |
| SNX19 | 3 |
| SPCS1 | 3 |
| SPG7 | 3 |
| SPHK2 | 3 |
| SPR | 3 |
| SRGN | 3 |
| STAU1 | 3 |
| TBX2 | 3 |
| TCN2 | 3 |
| TESC | 3 |
| TGM2 | 3 |
| TM9SF2 | 3 |
| TMEM204 | 3 |
| TNNT1 | 3 |
| TRIM22 | 3 |
| TRIM33 | 3 |
| TTC14 | 3 |
| UBE2G1 | 3 |
| UBL7 | 3 |
| UBQLN4 | 3 |
| UQCRH | 3 |
| VAMP5 | 3 |
| VPS4A | 3 |
| XAF1 | 3 |
| XBP1 | 3 |
| ZC3HAV1 | 3 |
| ZNF358 | 3 |
| A4GALT | 2 |
| AATF | 2 |
| ABCB1 | 2 |
| ABCC8 | 2 |
| ABCF1 | 2 |
| ACADVL | 2 |
| ACSBG1 | 2 |
| ACSL4 | 2 |
| ACSL5 | 2 |
| ACSS1 | 2 |
| ACTA2 | 2 |
| ACTR10 | 2 |
| ACTR5 | 2 |
| ACVRL1 | 2 |
| ADAM15 | 2 |
| ADAM33 | 2 |
| ADAMTS9 | 2 |
| ADNP | 2 |
| ADRA2C | 2 |
| AFF4 | 2 |
| AGER | 2 |
| AGTRAP | 2 |
| AHSA1 | 2 |
| AIF1 | 2 |
| AIMP2 | 2 |
| AKAP11 | 2 |
| AKAP13 | 2 |
| ALDH1L1 | 2 |
| ALDH1L2 | 2 |
| ALDOC | 2 |
| ALMS1 | 2 |
| ALMS1P1 | 2 |
| ALOX15B | 2 |
| AMD1 | 2 |
| ANKH | 2 |
| ANKRD12 | 2 |
| ANKRD13D | 2 |
| ANKRD50 | 2 |
| ANKRD9 | 2 |
| ANP32A | 2 |
| ANP32B | 2 |
| ANPEP | 2 |
| ANXA1 | 2 |
| AP1G2 | 2 |
| AP1S1 | 2 |
| AP1S2 | 2 |
| AP3M2 | 2 |
| AP5S1 | 2 |
| APBA2 | 2 |
| APBB1IP | 2 |
| APOL1 | 2 |
| APOL6 | 2 |
| ARAP3 | 2 |
| ARC | 2 |
| ARCN1 | 2 |
| ARF3 | 2 |
| ARF5 | 2 |
| ARFGEF2 | 2 |
| ARGLU1 | 2 |
| ARHGAP9 | 2 |
| ARHGEF7 | 2 |
| ARID3A | 2 |
| ARID5B | 2 |
| ASB13 | 2 |
| ASGR1 | 2 |
| ASPDH | 2 |
| ASPHD1 | 2 |
| ASPSCR1 | 2 |
| ATAD1 | 2 |
| ATF3 | 2 |
| ATHL1 | 2 |
| ATP11B | 2 |
| ATP5A1 | 2 |
| ATP5C1 | 2 |
| ATP5D | 2 |
| ATP5EP2 | 2 |
| ATP5F1P5 | 2 |
| ATP5I | 2 |
| ATP6V1G1 | 2 |
| BAALC | 2 |
| BAI2 | 2 |
| BAT2D1 | 2 |
| BATF2 | 2 |
| BCL2L1 | 2 |
| BCL6 | 2 |
| BCL7C | 2 |
| BEND4 | 2 |
| BEX1 | 2 |
| BEX4 | 2 |
| BLOC1S1 | 2 |
| BMPR2 | 2 |
| BORCS7 | 2 |
| BRD3 | 2 |
| BST2 | 2 |
| BTAF1 | 2 |
| C10orf116 | 2 |
| C11orf96 | 2 |
| C12orf65 | 2 |
| C1orf173 | 2 |
| C1orf61 | 2 |
| C1orf77 | 2 |
| C1QL2 | 2 |
| C1QL3 | 2 |
| C1QTNF1 | 2 |
| C1QTNF4 | 2 |
| C1QTNF6 | 2 |
| C20orf54 | 2 |
| C2CD2 | 2 |
| C5orf63 | 2 |
| C6orf223 | 2 |
| C9orf142 | 2 |
| C9orf50 | 2 |
| C9orf84 | 2 |
| CACNA2D3 | 2 |
| CALB1 | 2 |
| CALM2 | 2 |
| CALM3 | 2 |
| CAPZB | 2 |
| CARD11 | 2 |
| CARM1 | 2 |
| CARTPT | 2 |
| CASP7 | 2 |
| CBFB | 2 |
| CBLB | 2 |
| CBWD1 | 2 |
| CCDC107 | 2 |
| CCDC122 | 2 |
| CCDC14 | 2 |
| CCL5 | 2 |
| CCND3 | 2 |
| CCNDBP1 | 2 |
| CCNG2 | 2 |
| CCNI | 2 |
| CCNL1 | 2 |
| CCNT2 | 2 |
| CCNT2-AS1 | 2 |
| CCT2 | 2 |
| CD160 | 2 |
| CD163 | 2 |
| CD1D | 2 |
| CD52 | 2 |
| CD68 | 2 |
| CD99L2 | 2 |
| CDA | 2 |
| CDC20 | 2 |
| CDC2L5 | 2 |
| CDC42EP4 | 2 |
| CDH13 | 2 |
| CDH22 | 2 |
| CDH5 | 2 |
| CDK5RAP3 | 2 |
| CDKN1B | 2 |
| CEBPD | 2 |
| CERS5 | 2 |
| CFP | 2 |
| CGREF1 | 2 |
| CHD2 | 2 |
| CHD8 | 2 |
| CHI3L1 | 2 |
| CHI3L2 | 2 |
| CHKB | 2 |
| CHL1-AS1 | 2 |
| CHMP2A | 2 |
| CHMP5 | 2 |
| CHORDC1 | 2 |
| CKLF | 2 |
| CLC | 2 |
| CLDN14 | 2 |
| CLDND1 | 2 |
| CLEC11A | 2 |
| CLEC18B | 2 |
| CLEC3B | 2 |
| CLIC3 | 2 |
| CLINT1 | 2 |
| CLK1 | 2 |
| CLPTM1 | 2 |
| CMTM7 | 2 |
| CNGB1 | 2 |
| CNN2 | 2 |
| CNNM2 | 2 |
| CNOT4 | 2 |
| CNPPD1 | 2 |
| CNR1 | 2 |
| COL11A2 | 2 |
| COL4A1 | 2 |
| COL4A2 | 2 |
| COPA | 2 |
| COPE | 2 |
| COX10 | 2 |
| COX5A | 2 |
| COX6C | 2 |
| COX7A1 | 2 |
| COX7A2 | 2 |
| COX7C | 2 |
| COX8A | 2 |
| CPD | 2 |
| CPLX1 | 2 |
| CPLX2 | 2 |
| CPT1A | 2 |
| CREBBP | 2 |
| CRHR1-IT1 | 2 |
| CRISPLD2 | 2 |
| CSE1L | 2 |
| CSRNP3 | 2 |
| CTBP2 | 2 |
| CTSF | 2 |
| CTSS | 2 |
| CTSZ | 2 |
| CUL9 | 2 |
| CXorf57 | 2 |
| CXXC1 | 2 |
| CXXC5 | 2 |
| CYB5A | 2 |
| CYB5D2 | 2 |
| CYB5R1 | 2 |
| CYBB | 2 |
| CYBRD1 | 2 |
| CYLD | 2 |
| CYP2D6 | 2 |
| CYP7B1 | 2 |
| CYR61 | 2 |
| DARS2 | 2 |
| DAZAP2 | 2 |
| DBI | 2 |
| DBNDD1 | 2 |
| DCLRE1C | 2 |
| DCP2 | 2 |
| DDIT4 | 2 |
| DEFA1 | 2 |
| DEFA3 | 2 |
| DGCR5 | 2 |
| DGKD | 2 |
| DHRS7B | 2 |
| DHRS9 | 2 |
| DHX35 | 2 |
| DIAPH2 | 2 |
| DLG2 | 2 |
| DNAH10OS | 2 |
| DNAH6 | 2 |
| DNAH9 | 2 |
| DNAJA1 | 2 |
| DNAJB1 | 2 |
| DNAJB12 | 2 |
| DNAJB2 | 2 |
| DNASE1L1 | 2 |
| DOCK6 | 2 |
| DOK7 | 2 |
| DOPEY1 | 2 |
| DOPEY2 | 2 |
| DPH5 | 2 |
| DPM3 | 2 |
| DPP4 | 2 |
| DRAM2 | 2 |
| DRAP1 | 2 |
| DTX2 | 2 |
| DTX3L | 2 |
| DUSP1 | 2 |
| DYNC1LI2 | 2 |
| DYNLL1 | 2 |
| DYNLRB1 | 2 |
| EBF4 | 2 |
| EDC4 | 2 |
| EDEM3 | 2 |
| EDN1 | 2 |
| EEF1D | 2 |
| EFHD2 | 2 |
| EFR3A | 2 |
| EFTUD1P1 | 2 |
| EGR1 | 2 |
| EHD2 | 2 |
| EIF2AK2 | 2 |
| EIF2C2 | 2 |
| EIF3EP1 | 2 |
| ELOVL1 | 2 |
| ELOVL6 | 2 |
| ELP2 | 2 |
| EMB | 2 |
| EMX1 | 2 |
| ENDOG | 2 |
| ENSA | 2 |
| EP300 | 2 |
| EPB42 | 2 |
| EPHB1 | 2 |
| EPHB6 | 2 |
| ERCC1 | 2 |
| ERCC5 | 2 |
| ERH | 2 |
| ESRRA | 2 |
| EVI2A | 2 |
| EVI5 | 2 |
| EXOSC10 | 2 |
| EXTL1 | 2 |
| FABP7 | 2 |
| FAM195B | 2 |
| FAM221A | 2 |
| FAM46A | 2 |
| FAM47E | 2 |
| FAM69A | 2 |
| FAM86B3P | 2 |
| FAM92B | 2 |
| FAM98B | 2 |
| FARP1 | 2 |
| FASTK | 2 |
| FAU | 2 |
| FBXO18 | 2 |
| FBXO33 | 2 |
| FBXO9 | 2 |
| FCGBP | 2 |
| FEM1C | 2 |
| FGD2 | 2 |
| FGF17 | 2 |
| FGR | 2 |
| FIBP | 2 |
| FJX1 | 2 |
| FKBP8 | 2 |
| FLJ38717 | 2 |
| FLNA | 2 |
| FMN2 | 2 |
| FOS | 2 |
| FOSB | 2 |
| FOXH1 | 2 |
| FOXN2 | 2 |
| FOXN3 | 2 |
| FOXO4 | 2 |
| FSTL3 | 2 |
| FTCD | 2 |
| FTCDNL1 | 2 |
| FUS | 2 |
| FUZ | 2 |
| FXR1 | 2 |
| FXYD7 | 2 |
| FYN | 2 |
| G0S2 | 2 |
| GABRB3 | 2 |
| GABRD | 2 |
| GADD45A | 2 |
| GADD45G | 2 |
| GAMT | 2 |
| GANAB | 2 |
| GBP1 | 2 |
| GBP4 | 2 |
| GDF1 | 2 |
| GFOD1 | 2 |
| GGT5 | 2 |
| GIGYF1 | 2 |
| GIMAP2 | 2 |
| GK | 2 |
| GLI4 | 2 |
| GLT8D1 | 2 |
| GMEB2 | 2 |
| GMPPA | 2 |
| GMPPB | 2 |
| GNAI3 | 2 |
| GNB3 | 2 |
| GNG11 | 2 |
| GNG2 | 2 |
| GNL3L | 2 |
| GNLY | 2 |
| GOLGA2P10 | 2 |
| GOPC | 2 |
| GOT1 | 2 |
| GPR171 | 2 |
| GPR177 | 2 |
| GPT | 2 |
| GPX3 | 2 |
| GPX4 | 2 |
| GRASP | 2 |
| GRB14 | 2 |
| GRIN3A | 2 |
| GRINA | 2 |
| GSTZ1 | 2 |
| GUK1 | 2 |
| GYG1 | 2 |
| H19 | 2 |
| H2AFZ | 2 |
| HAPLN3 | 2 |
| HBA1 | 2 |
| HBA2 | 2 |
| HBB | 2 |
| HBEGF | 2 |
| HBG1 | 2 |
| HBM | 2 |
| HBQ1 | 2 |
| HEBP2 | 2 |
| HERC1 | 2 |
| HHEX | 2 |
| HIF1A | 2 |
| HIGD2A | 2 |
| HINT2 | 2 |
| HIRA | 2 |
| HIST1H1C | 2 |
| HIST1H2BC | 2 |
| HIST1H2BG | 2 |
| HIST1H3H | 2 |
| HIST2H2AA3 | 2 |
| HIST2H2BE | 2 |
| HK3 | 2 |
| HLA-A | 2 |
| HLA-F | 2 |
| HM13 | 2 |
| HNRNPA2B1 | 2 |
| HNRNPH3 | 2 |
| HNRNPM | 2 |
| HOOK1 | 2 |
| HR | 2 |
| HRC | 2 |
| HS3ST1 | 2 |
| HSP90AA1 | 2 |
| HSPA4L | 2 |
| HSPA6 | 2 |
| HSPA8 | 2 |
| HSPBP1 | 2 |
| HSPD1 | 2 |
| HSPE1 | 2 |
| HSPH1 | 2 |
| HYAL2 | 2 |
| HYI | 2 |
| IARS | 2 |
| ICAM1 | 2 |
| ICAM2 | 2 |
| ID3 | 2 |
| IER5 | 2 |
| IFI27 | 2 |
| IFI35 | 2 |
| IFI44 | 2 |
| IFI44L | 2 |
| IFI6 | 2 |
| IFIT3 | 2 |
| IFNAR1 | 2 |
| IFNGR1 | 2 |
| IGFBP4 | 2 |
| IGFBP7 | 2 |
| IK | 2 |
| IL1B | 2 |
| IL32 | 2 |
| IL4R | 2 |
| IL7 | 2 |
| ILK | 2 |
| INA | 2 |
| INPP5A | 2 |
| INSIG1 | 2 |
| INTS3 | 2 |
| IRAK3 | 2 |
| IRF1 | 2 |
| IRF7 | 2 |
| IRF9 | 2 |
| ISG15 | 2 |
| ITGAL | 2 |
| ITM2A | 2 |
| ITM2C | 2 |
| ITPR3 | 2 |
| JMJD6 | 2 |
| JMY | 2 |
| JRK | 2 |
| JUNB | 2 |
| KANK2 | 2 |
| KANSL1 | 2 |
| KANSL1-AS1 | 2 |
| KCNE4 | 2 |
| KCNG1 | 2 |
| KCNJ13 | 2 |
| KCNJ6 | 2 |
| KCTD21 | 2 |
| KIAA0020 | 2 |
| KIAA0922 | 2 |
| KIAA1147 | 2 |
| KIAA1958 | 2 |
| KIAA2010 | 2 |
| KIF17 | 2 |
| KIF1BP | 2 |
| KIF1C | 2 |
| KIF5B | 2 |
| KIFAP3 | 2 |
| KIFC2 | 2 |
| KLF6 | 2 |
| KLF9 | 2 |
| KLHDC4 | 2 |
| KLHL3 | 2 |
| KLHL9 | 2 |
| KPNA3 | 2 |
| KPNA4 | 2 |
| KTN1 | 2 |
| L1CAM | 2 |
| LACTB | 2 |
| LATS2 | 2 |
| LCN2 | 2 |
| LEMD3 | 2 |
| LINC00202 | 2 |
| LINC00320 | 2 |
| LINC00339 | 2 |
| LINC00443 | 2 |
| LINC00664 | 2 |
| LINC00667 | 2 |
| LMAN1 | 2 |
| LMNA | 2 |
| LMO2 | 2 |
| LMTK2 | 2 |
| LNPEP | 2 |
| LOC285636 | 2 |
| LOC338829 | 2 |
| LRGUK | 2 |
| LRP10 | 2 |
| LRP2BP | 2 |
| LRP3 | 2 |
| LRRC37A4P | 2 |
| LRRFIP2 | 2 |
| LRRN3 | 2 |
| LSM7 | 2 |
| LY6E | 2 |
| LY6H | 2 |
| LYSMD4 | 2 |
| MAFF | 2 |
| MAFK | 2 |
| MAGED2 | 2 |
| MAMSTR | 2 |
| MAN2A1 | 2 |
| MAP2K3 | 2 |
| MAP4K4 | 2 |
| MAPK11 | 2 |
| MAPK12 | 2 |
| MAPKAP1 | 2 |
| MARCKSL1 | 2 |
| MATK | 2 |
| MAU2 | 2 |
| MBD3 | 2 |
| MBNL1 | 2 |
| MCM6 | 2 |
| MCOLN1 | 2 |
| MCOLN2 | 2 |
| MDH1 | 2 |
| MED23 | 2 |
| MED29 | 2 |
| MED30 | 2 |
| MEF2C | 2 |
| MEF2D | 2 |
| MESTP1 | 2 |
| METTL7A | 2 |
| MGA | 2 |
| MGC13005 | 2 |
| MIAT | 2 |
| MIDN | 2 |
| MIR143HG | 2 |
| MIRLET7BHG | 2 |
| MKLN1 | 2 |
| MMD | 2 |
| MREG | 2 |
| MRFAP1L1 | 2 |
| MRM2 | 2 |
| MRPL11 | 2 |
| MRPL28 | 2 |
| MRPL3 | 2 |
| MRPL32 | 2 |
| MRPL40 | 2 |
| MRPS30 | 2 |
| MSN | 2 |
| MST1P2 | 2 |
| MSX1 | 2 |
| MT1F | 2 |
| MTDH | 2 |
| MTMR11 | 2 |
| MTRNR2L2 | 2 |
| MTRNR2L3 | 2 |
| MUC6 | 2 |
| MX1 | 2 |
| MXD1 | 2 |
| MYL6 | 2 |
| N4BP2 | 2 |
| N4BP3 | 2 |
| NAALAD2 | 2 |
| NADK | 2 |
| NAE1 | 2 |
| NAGPA | 2 |
| NATD1 | 2 |
| NBPF20 | 2 |
| NDE1 | 2 |
| NDFIP2 | 2 |
| NDRG1 | 2 |
| NDRG2 | 2 |
| NDRG3 | 2 |
| NDUFA1 | 2 |
| NDUFA3 | 2 |
| NDUFA4 | 2 |
| NDUFA4L2 | 2 |
| NDUFAF3 | 2 |
| NDUFAF4 | 2 |
| NDUFB5 | 2 |
| NDUFB7 | 2 |
| NDUFS4 | 2 |
| NDUFS5 | 2 |
| NECAB3 | 2 |
| NECAP1 | 2 |
| NEK4 | 2 |
| NEU1 | 2 |
| NFE2L2 | 2 |
| NFKB2 | 2 |
| NFKBIA | 2 |
| NGEF | 2 |
| NGRN | 2 |
| NID1 | 2 |
| NIN | 2 |
| NIP7 | 2 |
| NIPSNAP1 | 2 |
| NIT2 | 2 |
| NKTR | 2 |
| NME1 | 2 |
| NME1-NME2 | 2 |
| NMRAL1 | 2 |
| NNAT | 2 |
| NOTCH2NL | 2 |
| NPDC1 | 2 |
| NPIP | 2 |
| NPIPB4 | 2 |
| NPPA | 2 |
| NPTX2 | 2 |
| NR1D2 | 2 |
| NSF | 2 |
| NT5DC3 | 2 |
| NUB1 | 2 |
| NUDC | 2 |
| NUDT16 | 2 |
| NUDT22 | 2 |
| NUDT3 | 2 |
| NUFIP2 | 2 |
| OAS3 | 2 |
| OASL | 2 |
| OLFML2B | 2 |
| OLR1 | 2 |
| OMG | 2 |
| OPRK1 | 2 |
| ORMDL1 | 2 |
| OTUD4 | 2 |
| OXTR | 2 |
| P4HA1 | 2 |
| PAPD5 | 2 |
| PARD6G | 2 |
| PARP14 | 2 |
| PARP9 | 2 |
| PBXIP1 | 2 |
| PCCB | 2 |
| PCDHB5 | 2 |
| PCMTD1 | 2 |
| PCNA | 2 |
| PDLIM2 | 2 |
| PEA15 | 2 |
| PECAM1 | 2 |
| PELI1 | 2 |
| PFDN2 | 2 |
| PGF | 2 |
| PGP | 2 |
| PHACTR2 | 2 |
| PHF11 | 2 |
| PI3 | 2 |
| PIAS4 | 2 |
| PIBF1 | 2 |
| PIK3CD | 2 |
| PITPNB | 2 |
| PKNOX1 | 2 |
| PKP4 | 2 |
| PLA1A | 2 |
| PLEKHB1 | 2 |
| PLEKHB2 | 2 |
| PLEKHF1 | 2 |
| PLEKHG1 | 2 |
| PLEKHG2 | 2 |
| PLEKHO2 | 2 |
| PLSCR1 | 2 |
| PLXNC1 | 2 |
| PNMT | 2 |
| PNOC | 2 |
| PNPLA7 | 2 |
| PODXL | 2 |
| POLE4 | 2 |
| POU3F1 | 2 |
| PPA1 | 2 |
| PPAT | 2 |
| PPIL1 | 2 |
| PPIP5K1 | 2 |
| PPM1B | 2 |
| PPP1CA | 2 |
| PPP1R17 | 2 |
| PPP1R3B | 2 |
| PRDX2 | 2 |
| PRDX5 | 2 |
| PRIC285 | 2 |
| PRKCD | 2 |
| PRKCE | 2 |
| PRKCSH | 2 |
| PRMT7 | 2 |
| PRODH | 2 |
| PRPF8 | 2 |
| PRRT3 | 2 |
| PRSS23 | 2 |
| PSENEN | 2 |
| PSIP1 | 2 |
| PSMA4 | 2 |
| PSMB8 | 2 |
| PSMB9 | 2 |
| PSTPIP2 | 2 |
| PTBP3 | 2 |
| PTER | 2 |
| PTGES2 | 2 |
| PTGS2 | 2 |
| PTH1R | 2 |
| PTMS | 2 |
| PTP4A3 | 2 |
| PTPLAD2 | 2 |
| PTPRN | 2 |
| PTRF | 2 |
| PUF60 | 2 |
| PXN | 2 |
| PYDC1 | 2 |
| QDPR | 2 |
| RAB11FIP1 | 2 |
| RAB13 | 2 |
| RAB27A | 2 |
| RAB37 | 2 |
| RAB3C | 2 |
| RAB40C | 2 |
| RAB8B | 2 |
| RABEP1 | 2 |
| RAD51C | 2 |
| RALY-AS1 | 2 |
| RANBP10 | 2 |
| RAPGEF1 | 2 |
| RARA | 2 |
| RARRES3 | 2 |
| RASA1 | 2 |
| RASGRP1 | 2 |
| RASGRP2 | 2 |
| RASIP1 | 2 |
| RBBP5 | 2 |
| RBM14 | 2 |
| RBM3 | 2 |
| RBM41 | 2 |
| RBPMS | 2 |
| RBX1 | 2 |
| RCBTB1 | 2 |
| RCN2 | 2 |
| RCOR3 | 2 |
| RDH14 | 2 |
| REEP5 | 2 |
| RELB | 2 |
| REPS2 | 2 |
| RERE | 2 |
| RGS1 | 2 |
| RGS2 | 2 |
| RHOC | 2 |
| RHOQ | 2 |
| RIMS3 | 2 |
| RIN3 | 2 |
| RIT2 | 2 |
| RNASE1 | 2 |
| RNASE3 | 2 |
| RNF10 | 2 |
| RNF111 | 2 |
| RNF19A | 2 |
| RNF24 | 2 |
| RNF38 | 2 |
| ROBO2 | 2 |
| RORA | 2 |
| RP9P | 2 |
| RPL11 | 2 |
| RPL15 | 2 |
| RPL18 | 2 |
| RPL26 | 2 |
| RPL27 | 2 |
| RPL29 | 2 |
| RPL3 | 2 |
| RPL39L | 2 |
| RPL41 | 2 |
| RPLP0 | 2 |
| RPLP1 | 2 |
| RPRM | 2 |
| RPS15 | 2 |
| RPS17 | 2 |
| RPS21 | 2 |
| RPS3 | 2 |
| RPS6 | 2 |
| RPS6KA6 | 2 |
| RPS6KB2 | 2 |
| RPSA | 2 |
| RRAS | 2 |
| RRN3 | 2 |
| RUNX3 | 2 |
| RWDD2B | 2 |
| RYBP | 2 |
| S100A1 | 2 |
| S100A10 | 2 |
| S100A12 | 2 |
| S100A8 | 2 |
| S100A9 | 2 |
| S1PR5 | 2 |
| SCAND1 | 2 |
| SCN1B | 2 |
| SCN3B | 2 |
| SCRT1 | 2 |
| SDCCAG8 | 2 |
| SDHALP1 | 2 |
| SDHD | 2 |
| SEC11A | 2 |
| SEC14L1 | 2 |
| SEC16A | 2 |
| SEC24C | 2 |
| SEC61A1 | 2 |
| SEC63 | 2 |
| SELENBP1 | 2 |
| SELPLG | 2 |
| SEMA3C | 2 |
| SEMA4B | 2 |
| SEPW1 | 2 |
| SERF2 | 2 |
| SERP1 | 2 |
| SERPINH1 | 2 |
| SERTAD1 | 2 |
| SERTM1 | 2 |
| SETD6 | 2 |
| SF3B1 | 2 |
| SFRP2 | 2 |
| SGK1 | 2 |
| SGSM2 | 2 |
| SH2D3C | 2 |
| SH3BP1 | 2 |
| SH3GL2 | 2 |
| SHC2 | 2 |
| SHISA4 | 2 |
| SIK1 | 2 |
| SKAP1 | 2 |
| SKAP2 | 2 |
| SLC17A6 | 2 |
| SLC25A1 | 2 |
| SLC25A27 | 2 |
| SLC25A41 | 2 |
| SLC25A45 | 2 |
| SLC25A5 | 2 |
| SLC35F3 | 2 |
| SLC35F6 | 2 |
| SLC39A7 | 2 |
| SLC4A7 | 2 |
| SLC9A3R2 | 2 |
| SLC9C2 | 2 |
| SLIT2 | 2 |
| SLPI | 2 |
| SMAD6 | 2 |
| SMG1 | 2 |
| SMG1P5 | 2 |
| SNAI3 | 2 |
| SNAP25 | 2 |
| SNAPC2 | 2 |
| SNAPC3 | 2 |
| SNTB2 | 2 |
| SNX10 | 2 |
| SNX17 | 2 |
| SNX6 | 2 |
| SORBS3 | 2 |
| SORD | 2 |
| SPATA18 | 2 |
| SPATA21 | 2 |
| SPEN | 2 |
| SPHKAP | 2 |
| SPINK2 | 2 |
| SPINT1 | 2 |
| SPOCK2 | 2 |
| SPPL3 | 2 |
| SQLE | 2 |
| SRA1 | 2 |
| SRGAP2 | 2 |
| SRP9 | 2 |
| SRRM2 | 2 |
| SSR4 | 2 |
| SST | 2 |
| ST3GAL1 | 2 |
| ST6GAL1 | 2 |
| ST7-OT4 | 2 |
| STAG2 | 2 |
| STARD3 | 2 |
| STAT1 | 2 |
| STAT5A | 2 |
| STIP1 | 2 |
| STK17B | 2 |
| STK38 | 2 |
| STK4 | 2 |
| STK40 | 2 |
| STOX1 | 2 |
| STT3B | 2 |
| SUMF2 | 2 |
| SUMO2P17 | 2 |
| SUPV3L1 | 2 |
| SURF1 | 2 |
| SYNJ2BP | 2 |
| SYP | 2 |
| SYT1 | 2 |
| SYT11 | 2 |
| SYT5 | 2 |
| TACC3 | 2 |
| TAF11 | 2 |
| TAF15 | 2 |
| TAGLN | 2 |
| TAGLN2 | 2 |
| TANK | 2 |
| TAP1 | 2 |
| TAPBP | 2 |
| TARDBP | 2 |
| TARS2 | 2 |
| TAX1BP1 | 2 |
| TBC1D15 | 2 |
| TCF20 | 2 |
| TCF23 | 2 |
| TCN1 | 2 |
| TCTA | 2 |
| TCTE1 | 2 |
| TGFBR2 | 2 |
| TGFBR3 | 2 |
| THAP7 | 2 |
| THOP1 | 2 |
| THRA | 2 |
| TIMP1 | 2 |
| TINAGL1 | 2 |
| TJP2 | 2 |
| TKT | 2 |
| TM2D1 | 2 |
| TMED10 | 2 |
| TMED2 | 2 |
| TMED4 | 2 |
| TMED5 | 2 |
| TMED7 | 2 |
| TMEM123 | 2 |
| TMEM14A | 2 |
| TMEM14B | 2 |
| TMEM14C | 2 |
| TMEM155 | 2 |
| TMEM170B | 2 |
| TMEM181 | 2 |
| TMEM196 | 2 |
| TMEM200B | 2 |
| TMEM38A | 2 |
| TMEM59 | 2 |
| TMEM69 | 2 |
| TMEM79 | 2 |
| TMEM86B | 2 |
| TMSB4X | 2 |
| TNFAIP2 | 2 |
| TNFAIP6 | 2 |
| TNFAIP8L3 | 2 |
| TNFRSF10B | 2 |
| TNFRSF10D | 2 |
| TNFRSF11B | 2 |
| TNFSF10 | 2 |
| TNFSF13B | 2 |
| TNFSF14 | 2 |
| TNK2 | 2 |
| TNRC6B | 2 |
| TP53I13 | 2 |
| TP53INP2 | 2 |
| TPT1 | 2 |
| TRA2A | 2 |
| TRABD | 2 |
| TRAF4 | 2 |
| TRAM1 | 2 |
| TRHDE-AS1 | 2 |
| TRIM26 | 2 |
| TRIM35 | 2 |
| TRIM37 | 2 |
| TRIM41 | 2 |
| TRIP10 | 2 |
| TRIP6 | 2 |
| TRIT1 | 2 |
| TRMT61A | 2 |
| TRMT61B | 2 |
| TRPV2 | 2 |
| TSC22D1 | 2 |
| TSPAN4 | 2 |
| TSPYL2 | 2 |
| TSR2 | 2 |
| TSSC4 | 2 |
| TSTA3 | 2 |
| TTYH1 | 2 |
| TUBA1C | 2 |
| TUBB2C | 2 |
| TVP23B | 2 |
| TXN | 2 |
| TXNDC11 | 2 |
| TXNDC17 | 2 |
| UBA52 | 2 |
| UBE2J1 | 2 |
| UBE2Q2L | 2 |
| UBE2Q2P11 | 2 |
| UBE2Q2P12 | 2 |
| UBE2Z | 2 |
| UBN2 | 2 |
| UBP1 | 2 |
| UBXN2B | 2 |
| UBXN6 | 2 |
| UCP2 | 2 |
| UPP1 | 2 |
| UQCRFS1 | 2 |
| UQCRQ | 2 |
| USP16 | 2 |
| USP24 | 2 |
| USP7 | 2 |
| VASH2 | 2 |
| VASP | 2 |
| VEGFB | 2 |
| VIT | 2 |
| VPS35 | 2 |
| VPS37B | 2 |
| VWCE | 2 |
| WAC-AS1 | 2 |
| WASF2 | 2 |
| WBP1 | 2 |
| WBP2NL | 2 |
| WDR13 | 2 |
| WDR45 | 2 |
| WDR73 | 2 |
| WDR75 | 2 |
| YJEFN3 | 2 |
| YPEL5 | 2 |
| YWHAB | 2 |
| YY1AP1 | 2 |
| ZBED5 | 2 |
| ZBTB38 | 2 |
| ZC3H5 | 2 |
| ZC3H6 | 2 |
| ZC3H7B | 2 |
| ZEB2 | 2 |
| ZGLP1 | 2 |
| ZMPSTE24 | 2 |
| ZNF112 | 2 |
| ZNF275 | 2 |
| ZNF385A | 2 |
| ZNF407 | 2 |
| ZNF429 | 2 |
| ZNF484 | 2 |
| ZNF493 | 2 |
| ZNF512 | 2 |
| ZNF534 | 2 |
| ZNF615 | 2 |
| ZNF738 | 2 |
| ZNF804A | 2 |
| ZNF821 | 2 |
| ZNF827 | 2 |
| ZNFX1 | 2 |
| ZRANB2 | 2 |
| ZRSR2 | 2 |
| ZSCAN29 | 2 |
| ZYX | 2 |
| A2LD1 | 1 |
| AAKI | 1 |
| AATK | 1 |
| ABCA13 | 1 |
| ABCA17P | 1 |
| ABCA7 | 1 |
| ABCB9 | 1 |
| ABCC12 | 1 |
| ABCC13 | 1 |
| ABCD4 | 1 |
| ABCE1 | 1 |
| ABCG2 | 1 |
| ABHD12 | 1 |
| ABHD14A | 1 |
| ABHD16A | 1 |
| ABHD5 | 1 |
| ABI3 | 1 |
| ABL2 | 1 |
| ABLIM1 | 1 |
| ABTB1 | 1 |
| ABTB2 | 1 |
| AC004540.4 | 1 |
| AC005841.1 | 1 |
| AC006160.5 | 1 |
| AC006547.13 | 1 |
| AC007899.3 | 1 |
| AC009133.17 | 1 |
| AC010761.10 | 1 |
| AC010761.9 | 1 |
| AC015688.3 | 1 |
| AC074289.1 | 1 |
| AC110781.3 | 1 |
| ACADM | 1 |
| ACAP2 | 1 |
| ACAP3 | 1 |
| ACAT1 | 1 |
| ACAT2 | 1 |
| ACBD3 | 1 |
| ACBD6 | 1 |
| ACCN4 | 1 |
| ACER2 | 1 |
| ACIN1 | 1 |
| ACKR3 | 1 |
| ACN9 | 1 |
| ACOT1 | 1 |
| ACOT4 | 1 |
| ACOT7 | 1 |
| ACOT9 | 1 |
| ACOX1 | 1 |
| ACOXL | 1 |
| ACP1 | 1 |
| ACP2 | 1 |
| ACRBP | 1 |
| ACRC | 1 |
| ACSM2B | 1 |
| ACSS2 | 1 |
| ACTG1 | 1 |
| ACTL6B | 1 |
| ACTN1 | 1 |
| ACTN4 | 1 |
| ACTR1B | 1 |
| ACTR2 | 1 |
| ACTR3B | 1 |
| ACTRT1 | 1 |
| ACVR1C | 1 |
| ACVR2A | 1 |
| ACY3 | 1 |
| ACYP1 | 1 |
| ACYP2 | 1 |
| ADAM10 | 1 |
| ADAM11 | 1 |
| ADAM23 | 1 |
| ADAM8 | 1 |
| ADAMTS18 | 1 |
| ADAMTS2 | 1 |
| ADAMTS7 | 1 |
| ADAMTS9-AS1 | 1 |
| ADAMTS9-AS2 | 1 |
| ADAMTSL1 | 1 |
| ADAMTSL4 | 1 |
| ADAR | 1 |
| ADCK5 | 1 |
| ADCY10P1 | 1 |
| ADCY2 | 1 |
| ADCY4 | 1 |
| ADCYAP1 | 1 |
| ADD3 | 1 |
| ADHFE1 | 1 |
| ADIPOR1 | 1 |
| ADM | 1 |
| ADORA1 | 1 |
| ADORA2A | 1 |
| ADRA1B | 1 |
| ADRA2A | 1 |
| ADRBK1 | 1 |
| ADRM1 | 1 |
| AE000661.37 | 1 |
| AEBP1 | 1 |
| AEBP2 | 1 |
| AES | 1 |
| AFF2 | 1 |
| AGFG1 | 1 |
| AGL | 1 |
| AGPAT1 | 1 |
| AGPAT2 | 1 |
| AGPAT3 | 1 |
| AGRN | 1 |
| AGTPBP1 | 1 |
| AHCTF1 | 1 |
| AHR | 1 |
| AICDA | 1 |
| AIDA | 1 |
| AIF1L | 1 |
| AIFM2 | 1 |
| AIP | 1 |
| AK2 | 1 |
| AK4 | 1 |
| AK8 | 1 |
| AKAP12 | 1 |
| AKAP7 | 1 |
| AKAP8 | 1 |
| AKAP8L | 1 |
| AKIRIN1 | 1 |
| AKNA | 1 |
| AKR1B1 | 1 |
| AKR7A2 | 1 |
| AKT1 | 1 |
| ALCAM | 1 |
| ALDH1A1 | 1 |
| ALDOA | 1 |
| ALDOAP2 | 1 |
| ALG1 | 1 |
| ALG10 | 1 |
| ALG8 | 1 |
| ALG9 | 1 |
| ALGIO | 1 |
| ALKBH3 | 1 |
| ALPK1 | 1 |
| ALPP | 1 |
| ALS2CR12 | 1 |
| AMACR | 1 |
| AMDHD2 | 1 |
| AMER1 | 1 |
| AMH | 1 |
| AMMECRI | 1 |
| AMPH | 1 |
| ANAPC10 | 1 |
| ANAPC10P1 | 1 |
| ANAPC15 | 1 |
| ANAPC2 | 1 |
| ANG | 1 |
| ANGEL2 | 1 |
| ANGPTL2 | 1 |
| ANGPTL3 | 1 |
| ANGPTL4 | 1 |
| ANGPTL6 | 1 |
| ANK1 | 1 |
| ANKIB1 | 1 |
| ANKLE2 | 1 |
| ANKMY1 | 1 |
| ANKRD10 | 1 |
| ANKRD17 | 1 |
| ANKRD19P | 1 |
| ANKRD22 | 1 |
| ANKRD28 | 1 |
| ANKRD30B | 1 |
| ANKRD32 | 1 |
| ANKRD33 | 1 |
| ANKRD34C | 1 |
| ANKRD46 | 1 |
| ANKRD56 | 1 |
| ANKRD6 | 1 |
| ANKRD65 | 1 |
| ANO5 | 1 |
| ANO8 | 1 |
| ANTXR2 | 1 |
| ANXA2 | 1 |
| ANXA2P2 | 1 |
| ANXA3 | 1 |
| ANXA6 | 1 |
| ANXA7 | 1 |
| ANXA8L1 | 1 |
| AOC2 | 1 |
| AOC3 | 1 |
| AP001610.5 | 1 |
| AP2S1 | 1 |
| AP3M1 | 1 |
| AP4B1 | 1 |
| APAF1 | 1 |
| APBA3 | 1 |
| APBB2 | 1 |
| APEX1 | 1 |
| API5 | 1 |
| APIGI | 1 |
| APLF | 1 |
| APOA1BP | 1 |
| APOBEC4 | 1 |
| APOD | 1 |
| APOE | 1 |
| APOLD1 | 1 |
| APOM | 1 |
| APP | 1 |
| APPL2 | 1 |
| APRT | 1 |
| AQP6 | 1 |
| AQP9 | 1 |
| AQR | 1 |
| ARAF | 1 |
| ARF6 | 1 |
| ARFGAP1 | 1 |
| ARFGAP2 | 1 |
| ARFRP1 | 1 |
| ARHGAP1 | 1 |
| ARHGAP19 | 1 |
| ARHGAP20 | 1 |
| ARHGAP24 | 1 |
| ARHGAP25 | 1 |
| ARHGAP27 | 1 |
| ARHGAP29 | 1 |
| ARHGAP30 | 1 |
| ARHGAP31 | 1 |
| ARHGAP36 | 1 |
| ARHGAP5 | 1 |
| ARHGDIG | 1 |
| ARHGEF10L | 1 |
| ARHGEF28 | 1 |
| ARHGEF3 | 1 |
| ARHGEF4 | 1 |
| ARHGEF40 | 1 |
| ARID4A | 1 |
| ARID4B | 1 |
| ARID5A | 1 |
| ARIH1 | 1 |
| ARL14EP | 1 |
| ARL15 | 1 |
| ARL16 | 1 |
| ARL17A | 1 |
| ARL17P1 | 1 |
| ARL3 | 1 |
| ARL4C | 1 |
| ARL4D | 1 |
| ARL5A | 1 |
| ARL5B | 1 |
| ARL6IP4 | 1 |
| ARL6IP5 | 1 |
| ARL6IP6 | 1 |
| ARL8B | 1 |
| ARMC6 | 1 |
| ARMC8 | 1 |
| ARMCX3 | 1 |
| ARMCX6 | 1 |
| ARNT | 1 |
| ARNTL2 | 1 |
| ARPC1A | 1 |
| ARPC4 | 1 |
| ARPM1 | 1 |
| ARRDC2 | 1 |
| ARRDC3 | 1 |
| ARSD | 1 |
| ARSJ | 1 |
| ARVCF | 1 |
| AS3MT | 1 |
| ASAH1 | 1 |
| ASAP1 | 1 |
| ASAP3 | 1 |
| ASB9 | 1 |
| ASCC2 | 1 |
| ASGR2 | 1 |
| ASL | 1 |
| ASMTL | 1 |
| ASNS | 1 |
| ASNSD1 | 1 |
| ASPHD2 | 1 |
| ASTE1 | 1 |
| ASXL1 | 1 |
| ASXL3 | 1 |
| ATAD2 | 1 |
| ATAD3B | 1 |
| ATAD3C | 1 |
| ATE1-AS1 | 1 |
| ATF6B | 1 |
| ATG101 | 1 |
| ATG13 | 1 |
| ATG16L1 | 1 |
| ATG2A | 1 |
| ATG3 | 1 |
| ATG4D | 1 |
| ATGIO | 1 |
| ATM | 1 |
| ATOH7 | 1 |
| ATOH8 | 1 |
| ATP10A | 1 |
| ATP13A2 | 1 |
| ATP1A1 | 1 |
| ATP1A1-AS1 | 1 |
| ATP1A1OS | 1 |
| ATP1A3 | 1 |
| ATP1B2 | 1 |
| ATP1B3 | 1 |
| ATP1B3-AS1 | 1 |
| ATP2A2 | 1 |
| ATP2B2 | 1 |
| ATP2B4 | 1 |
| ATP5B | 1 |
| ATP5E | 1 |
| ATP5F1 | 1 |
| ATP5G1 | 1 |
| ATP5G3 | 1 |
| ATP5J | 1 |
| ATP5L | 1 |
| ATP5O | 1 |
| ATP5S | 1 |
| ATP5SL | 1 |
| ATP6V0A1 | 1 |
| ATP6V0C | 1 |
| ATP6V0D2 | 1 |
| ATP6V1A | 1 |
| ATP6V1B2 | 1 |
| ATP6V1E1 | 1 |
| ATP7A | 1 |
| ATP8A2 | 1 |
| ATP8B2 | 1 |
| ATPBD4 | 1 |
| ATPBD4-AS1 | 1 |
| ATPIF1 | 1 |
| ATRN | 1 |
| ATRX | 1 |
| ATXN10 | 1 |
| ATXN3 | 1 |
| ATXN7L3 | 1 |
| AUNIP | 1 |
| AURKAPS1 | 1 |
| AVP | 1 |
| AYP1p1 | 1 |
| AZGP1 | 1 |
| AZIN1 | 1 |
| AZU1 | 1 |
| B3GALT1 | 1 |
| B3GALT2 | 1 |
| B3GALT4 | 1 |
| B3GALT6 | 1 |
| B3GAT2 | 1 |
| B3GNT1 | 1 |
| B3GNT2 | 1 |
| B3GNT8 | 1 |
| B3GNT9 | 1 |
| B4GALT1 | 1 |
| B4GALT2 | 1 |
| B4GALT3 | 1 |
| B4GALT5 | 1 |
| B4GALT6 | 1 |
| B4GALT7 | 1 |
| B9D1 | 1 |
| BAAT | 1 |
| BABAM1 | 1 |
| BACE2 | 1 |
| BAD | 1 |
| BAG2 | 1 |
| BAG3 | 1 |
| BAI1 | 1 |
| BAI3 | 1 |
| BAIAP3 | 1 |
| BAK1 | 1 |
| BANF1 | 1 |
| BAP1 | 1 |
| BARHL2 | 1 |
| BAT3 | 1 |
| BATF3 | 1 |
| BAX | 1 |
| BAZ2B | 1 |
| BBX | 1 |
| BCAM | 1 |
| BCAN | 1 |
| BCAS1 | 1 |
| BCAT2 | 1 |
| BCCIP | 1 |
| BCL11B | 1 |
| BCL2 | 1 |
| BCL2A1 | 1 |
| BCL2L2 | 1 |
| BCL3 | 1 |
| BCL6B | 1 |
| BCOR | 1 |
| BCR | 1 |
| BCRP3 | 1 |
| BCYRN1 | 1 |
| BEST4 | 1 |
| BET1L | 1 |
| BFSP2-AS1 | 1 |
| BGN | 1 |
| BHLHA15 | 1 |
| BHLHE22 | 1 |
| BHLHE40 | 1 |
| BHLHE41 | 1 |
| BHMT | 1 |
| BICD1 | 1 |
| BIK | 1 |
| BIN2 | 1 |
| BIRC2 | 1 |
| BLOC1S6 | 1 |
| BLVRA | 1 |
| BLVRB | 1 |
| BMI1 | 1 |
| BMP1 | 1 |
| BMP2 | 1 |
| BMP4 | 1 |
| BMP8B | 1 |
| BMS1 | 1 |
| BNIP2 | 1 |
| BOLA1 | 1 |
| BOLA2 | 1 |
| BPGM | 1 |
| BRCA1 | 1 |
| BRD1 | 1 |
| BRI3BP | 1 |
| BRINP2 | 1 |
| BRIX1 | 1 |
| BRP44L | 1 |
| BRPF3 | 1 |
| BRSK1 | 1 |
| BRSK2 | 1 |
| BRWD1 | 1 |
| BRWD3 | 1 |
| BSG | 1 |
| BSN-AS2 | 1 |
| BTBD1 | 1 |
| BTBD10 | 1 |
| BTBD11 | 1 |
| BTBD6 | 1 |
| BTG2 | 1 |
| BTG3 | 1 |
| BTGI | 1 |
| BTN1A1 | 1 |
| BTN3A2 | 1 |
| BTN3A3 | 1 |
| BTNL8 | 1 |
| BTNL9 | 1 |
| BUB3 | 1 |
| BUD13 | 1 |
| BXDC1 | 1 |
| BZRAP1 | 1 |
| BZW2 | 1 |
| C10orf10 | 1 |
| C10orf105 | 1 |
| C10orf108 | 1 |
| C10orf11 | 1 |
| C10ORF11 | 1 |
| C10orf111 | 1 |
| C10orf125 | 1 |
| C10orf129 | 1 |
| C10orf31 | 1 |
| C10orf55 | 1 |
| C10orf91 | 1 |
| C11orf17 | 1 |
| C11orf30 | 1 |
| C11orf49 | 1 |
| C11orf63 | 1 |
| C11orf67 | 1 |
| C11orf88 | 1 |
| C12orf33 | 1 |
| C12ORF47 | 1 |
| C12orf57 | 1 |
| C12ORF57 | 1 |
| C12orf76 | 1 |
| C12orf77 | 1 |
| C13orf16 | 1 |
| C140rf1 n | 1 |
| C140rf168 | 1 |
| C14ORF100 | 1 |
| C14orf106 | 1 |
| C14ORF106 | 1 |
| C14orf11 | 1 |
| C14orf138 | 1 |
| C14ORF138 | 1 |
| C14orf139 | 1 |
| C14ORF156 | 1 |
| C14orf166 | 1 |
| C14ORF166 | 1 |
| C14ORF173 | 1 |
| C14orf178 | 1 |
| C14orf180 | 1 |
| C14orf79 | 1 |
| C15orf21 | 1 |
| C15orf28 | 1 |
| C15orf37 | 1 |
| C15ORF39 | 1 |
| C15orf41 | 1 |
| C15orf52 | 1 |
| C15orf59 | 1 |
| C15orf61 | 1 |
| C15orf62 | 1 |
| C16orf3 | 1 |
| C16ORF35 | 1 |
| C16orf42 | 1 |
| C16orf48 | 1 |
| C16orf58 | 1 |
| C16ORF61 | 1 |
| C16ORF63 | 1 |
| C16ORF73 | 1 |
| C16orf74 | 1 |
| C16orf79 | 1 |
| C16orf86 | 1 |
| C16orf89 | 1 |
| C16orf92 | 1 |
| C17orf100 | 1 |
| C17orf107 | 1 |
| C17ORF45 | 1 |
| C17orf49 | 1 |
| C17orf59 | 1 |
| C17ORF61 | 1 |
| C17orf62 | 1 |
| C17orf67 | 1 |
| C17orf78 | 1 |
| C17orf8 | 1 |
| C17orf84 | 1 |
| C17orf89 | 1 |
| C17orf95 | 1 |
| C17orf96 | 1 |
| C18ORF25 | 1 |
| C18orf32 | 1 |
| C18orf54 | 1 |
| C18orf55 | 1 |
| C19orf10 | 1 |
| C19orf12 | 1 |
| C19ORF22 | 1 |
| C19orf24 | 1 |
| C19orf31 | 1 |
| C19orf48 | 1 |
| C19orf53 | 1 |
| C19ORF59 | 1 |
| C19orf6 | 1 |
| C19orf62 | 1 |
| C19orf66 | 1 |
| C19orf68 | 1 |
| C19orf70 | 1 |
| C19orf71 | 1 |
| C19orf81 | 1 |
| C1D | 1 |
| C1orf108 | 1 |
| C1orf122 | 1 |
| C1orf123 | 1 |
| C1orf134 | 1 |
| C1orf147 | 1 |
| C1orf151 | 1 |
| C1orf152 | 1 |
| C1orf158 | 1 |
| C1orf167 | 1 |
| C1orf174 | 1 |
| C1orf182 | 1 |
| C1orf187 | 1 |
| C1orf192 | 1 |
| C1orf200 | 1 |
| C1orf222 | 1 |
| C1orf226 | 1 |
| C1orf228 | 1 |
| C1orf51 | 1 |
| C1orf53 | 1 |
| C1ORF55 | 1 |
| C1orf57 | 1 |
| C1ORF59 | 1 |
| C1orf64 | 1 |
| C1orf69 | 1 |
| C1orf74 | 1 |
| C1orf85 | 1 |
| C1QL1 | 1 |
| C1QTNF3 | 1 |
| C1QTNF5 | 1 |
| C1R | 1 |
| C1S | 1 |
| C20ORF108 | 1 |
| C20orf144 | 1 |
| C20orf166-AS1 | 1 |
| C20ORF199 | 1 |
| C20orf201 | 1 |
| C20orf203 | 1 |
| C20orf27 | 1 |
| C20ORF52 | 1 |
| C20ORF55 | 1 |
| C20orf85 | 1 |
| C21orf119 | 1 |
| C21orf2 | 1 |
| C21orf33 | 1 |
| C21ORF55 | 1 |
| C21orf58 | 1 |
| C21ORF58 | 1 |
| C21orf62 | 1 |
| C21orf63 | 1 |
| C21orf67 | 1 |
| C21ORF77 | 1 |
| C22orf1 | 1 |
| C22ORF13 | 1 |
| C22orf15 | 1 |
| C22orf24 | 1 |
| C22ORF25 | 1 |
| C22orf29 | 1 |
| C22orf36 | 1 |
| C22orf39 | 1 |
| C22orf45 | 1 |
| C22orf46 | 1 |
| C2CD2L | 1 |
| C2CD4B | 1 |
| C2ORF24 | 1 |
| C2orf30 | 1 |
| C2ORF64 | 1 |
| C2orf88 | 1 |
| C3 | 1 |
| C3ORF10 | 1 |
| C3orf19 | 1 |
| C3ORF21 | 1 |
| C3orf25 | 1 |
| C3orf45 | 1 |
| C3orf49 | 1 |
| C3orf52 | 1 |
| C3orf64 | 1 |
| C4orf22 | 1 |
| C4ORF34 | 1 |
| C5orf13 | 1 |
| C5orf15 | 1 |
| C5orf27 | 1 |
| C5ORF28 | 1 |
| C5ORF32 | 1 |
| C5orf39 | 1 |
| C5ORF4 | 1 |
| C5orf45 | 1 |
| C5orf51 | 1 |
| C5orf55 | 1 |
| C5orf62 | 1 |
| C60rf204 | 1 |
| C6orf103 | 1 |
| C6orf105 | 1 |
| C6orf106 | 1 |
| C6orf114 | 1 |
| C6orf145 | 1 |
| C6orf15 | 1 |
| C6ORF151 | 1 |
| C6ORF160 | 1 |
| C6ORF48 | 1 |
| C6ORF60 | 1 |
| C6ORF81 | 1 |
| C6orf89 | 1 |
| C7ORF23 | 1 |
| C7orf41 | 1 |
| C7ORF41 | 1 |
| C7orf49 | 1 |
| C7orf54 | 1 |
| C7orf61 | 1 |
| C80rf53 | 1 |
| C80RFK32 | 1 |
| C8G | 1 |
| C8orf33 | 1 |
| C8orf40 | 1 |
| C8ORF59 | 1 |
| C8orf73 | 1 |
| C8orf88 | 1 |
| C90rf25 | 1 |
| C9ORF106 | 1 |
| C9orf116 | 1 |
| C9orf128 | 1 |
| C9ORF130 | 1 |
| C9orf156 | 1 |
| C9ORF164 | 1 |
| C9orf171 | 1 |
| C9orf24 | 1 |
| C9orf38 | 1 |
| C9orf4 | 1 |
| C9orf69 | 1 |
| C9orf7 | 1 |
| C9ORF72 | 1 |
| CA1 | 1 |
| CA2 | 1 |
| CA4 | 1 |
| CAAP1 | 1 |
| CAB39 | 1 |
| CAB39L | 1 |
| CABIN1 | 1 |
| CABLES1 | 1 |
| CABP5 | 1 |
| CABP7 | 1 |
| CACNA1E | 1 |
| CACNA1H | 1 |
| CACNA1I | 1 |
| CACNA2D1 | 1 |
| CACNA2D3-AS1 | 1 |
| CACNB1 | 1 |
| CACNB2 | 1 |
| CACNG1 | 1 |
| CACNG2 | 1 |
| CACNG7 | 1 |
| CACYBP | 1 |
| CALB2 | 1 |
| CALCOCO1 | 1 |
| CALHM2 | 1 |
| CALM1 | 1 |
| CALML4 | 1 |
| CALN1 | 1 |
| CALR | 1 |
| CALR3 | 1 |
| CALU | 1 |
| CAMK2G | 1 |
| CAMK4 | 1 |
| CAMKK2 | 1 |
| CAMP | 1 |
| CANX | 1 |
| CAP1 | 1 |
| CAP2 | 1 |
| CAPG | 1 |
| CAPN1 | 1 |
| CAPN10-AS1 | 1 |
| CAPN12 | 1 |
| CAPNS1 | 1 |
| CAPRIN2 | 1 |
| CAPZA2 | 1 |
| CASC3 | 1 |
| CASC4 | 1 |
| CASK | 1 |
| CASKIN1 | 1 |
| CASP2 | 1 |
| CASP3 | 1 |
| CASQ1 | 1 |
| CASQ2 | 1 |
| CAST | 1 |
| CAV1 | 1 |
| CBLN2 | 1 |
| CBLN3 | 1 |
| CBR3 | 1 |
| CBR4 | 1 |
| CBWD3 | 1 |
| CBX3P2 | 1 |
| CBX4 | 1 |
| CBX5 | 1 |
| CBX6 | 1 |
| CC2D2B | 1 |
| CCDC11 | 1 |
| CCDC117 | 1 |
| CCDC121 | 1 |
| CCDC134 | 1 |
| CCDC148 | 1 |
| CCDC149 | 1 |
| CCDC151 | 1 |
| CCDC152 | 1 |
| CCDC157 | 1 |
| CCDC163P | 1 |
| CCDC18 | 1 |
| CCDC22 | 1 |
| CCDC28A | 1 |
| CCDC28B | 1 |
| CCDC33 | 1 |
| CCDC36 | 1 |
| CCDC40 | 1 |
| CCDC53 | 1 |
| CCDC57 | 1 |
| CCDC58 | 1 |
| CCDC59 | 1 |
| CCDC6 | 1 |
| CCDC60 | 1 |
| CCDC64 | 1 |
| CCDC69 | 1 |
| CCDC71 | 1 |
| CCDC72 | 1 |
| CCDC74A | 1 |
| CCDC84 | 1 |
| CCDC87 | 1 |
| CCDC92 | 1 |
| CCDC94 | 1 |
| CCDC95 | 1 |
| CCK | 1 |
| CCL17 | 1 |
| CCL2 | 1 |
| CCL22 | 1 |
| CCL25 | 1 |
| CCL26 | 1 |
| CCL4 | 1 |
| CCM2L | 1 |
| CCNA1 | 1 |
| CCNJP2 | 1 |
| CCNK | 1 |
| CCNO | 1 |
| CCNT1 | 1 |
| CCP110 | 1 |
| CCPG1 | 1 |
| CCR2 | 1 |
| CCR3 | 1 |
| CCR5 | 1 |
| CCR6 | 1 |
| CCR7 | 1 |
| CCR8 | 1 |
| CCRL1 | 1 |
| CCS | 1 |
| CCSER1 | 1 |
| CCT7 | 1 |
| CD164L2 | 1 |
| CD19 | 1 |
| CD1A | 1 |
| CD200 | 1 |
| CD22 | 1 |
| CD244 | 1 |
| CD248 | 1 |
| CD24P4 | 1 |
| CD27 | 1 |
| CD274 | 1 |
| CD276 | 1 |
| CD2AP | 1 |
| CD302 | 1 |
| CD320 | 1 |
| CD34 | 1 |
| CD36 | 1 |
| CD37 | 1 |
| CD3D | 1 |
| CD3E | 1 |
| CD40 | 1 |
| CD44 | 1 |
| CD48 | 1 |
| CD53 | 1 |
| CD55 | 1 |
| CD6 | 1 |
| CD63 | 1 |
| CD69 | 1 |
| CD7 | 1 |
| CD72 | 1 |
| CD74 | 1 |
| CD79A | 1 |
| CD79B | 1 |
| CD80 | 1 |
| CD9 | 1 |
| CD93 | 1 |
| CD97 | 1 |
| CDC14A | 1 |
| CDC25A | 1 |
| CDC34 | 1 |
| CDC37 | 1 |
| CDC40 | 1 |
| CDC42-IT1 | 1 |
| CDC42BPA | 1 |
| CDC42EP2 | 1 |
| CDC42EP5 | 1 |
| CDC42SE1 | 1 |
| CDC45 | 1 |
| CDC6 | 1 |
| CDCA7L | 1 |
| CDH10 | 1 |
| CDH12 | 1 |
| CDH6 | 1 |
| CDIP1 | 1 |
| CDIPT-AS1 | 1 |
| CDK12 | 1 |
| CDK16 | 1 |
| CDK2AP1 | 1 |
| CDK2AP2 | 1 |
| CDK4 | 1 |
| CDK5 | 1 |
| CDK5R1 | 1 |
| CDK6 | 1 |
| CDK8 | 1 |
| CDKL1 | 1 |
| CDKN2C | 1 |
| CDNF | 1 |
| CDPF1 | 1 |
| CDT1 | 1 |
| CEACAM19 | 1 |
| CEBPB | 1 |
| CEBPG | 1 |
| CEBPZ | 1 |
| CECR1 | 1 |
| CECR4 | 1 |
| CEL | 1 |
| CELSR3 | 1 |
| CENPN | 1 |
| CENTB1 | 1 |
| CENTB2 | 1 |
| CENTD1 | 1 |
| CENTD2 | 1 |
| CEP135 | 1 |
| CEP152 | 1 |
| CEP170 | 1 |
| CEP27 | 1 |
| CEP41 | 1 |
| CERK | 1 |
| CERS4 | 1 |
| CERS6 | 1 |
| CES2 | 1 |
| CFAP74 | 1 |
| CFB | 1 |
| CFD | 1 |
| CFDP1 | 1 |
| CFH | 1 |
| CFL1 | 1 |
| CGN | 1 |
| CHAC2 | 1 |
| CHCHD10 | 1 |
| CHCHD3 | 1 |
| CHCHD4P5 | 1 |
| CHD1 | 1 |
| CHD4 | 1 |
| CHESI | 1 |
| CHGA | 1 |
| CHGB | 1 |
| CHKA | 1 |
| CHL1 | 1 |
| CHMP1B | 1 |
| CHMP4A | 1 |
| CHMP4B | 1 |
| CHMP6 | 1 |
| CHMP7 | 1 |
| CHN1 | 1 |
| chr1:111211165-111211475 | 1 |
| chr1:173242521-173243135 | 1 |
| chr1:200369287-200369964 | 1 |
| chr1:219433064-219433395 | 1 |
| chr1:222772643-222773934 | 1 |
| chr1:233811305-233811449 | 1 |
| chr1:75031522-75032256 | 1 |
| chr1:88756751-88758035 | 1 |
| chr1:9486434-9486788 | 1 |
| chr10:10698973-10699923 | 1 |
| chr10:4895704-4895738 | 1 |
| chr10:64458248-64459606 | 1 |
| chr11:110338897-110339247 | 1 |
| chr11:23108398-23109094 | 1 |
| chr11:23110444-23110919 | 1 |
| chr11:30814414-30815284 | 1 |
| chr11:36723766-36725077 | 1 |
| chr11:36909383-36910198 | 1 |
| chr11:40131372-40132279 | 1 |
| chr11:6213786-6215186 | 1 |
| chr11:94867352-94868281 | 1 |
| chr12:24202643-24202921 | 1 |
| chr12:67254722-67255835 | 1 |
| chr12:83531509-83532107 | 1 |
| chr12:97355714-97355975 | 1 |
| chr13:31630185-31631247 | 1 |
| chr14:29667076-29668263 | 1 |
| chr14:29716629-29717806 | 1 |
| chr14:47305224-47306296 | 1 |
| chr15:88997885-88998833 | 1 |
| chr17:12052424-12052673 | 1 |
| chr17:68181339-68182321 | 1 |
| chr18:35230475-35230627 | 1 |
| chr19:2059605-2060167 | 1 |
| chr19:37171128-37171323 | 1 |
| chr2:152643269-152644424 | 1 |
| chr2:155717139-155717166 | 1 |
| chr2:195334490-195335546 | 1 |
| chr2:200876990-200877640 | 1 |
| chr2:206960282-206960679 | 1 |
| chr2:226533070-226533806 | 1 |
| chr2:239473151-239473253 | 1 |
| chr2:40917226-40918073 | 1 |
| chr2:8659514-8659740 | 1 |
| chr2:8661100-8661349 | 1 |
| chr20:57722243-57722385 | 1 |
| chr21:16170716-16170906 | 1 |
| chr21:27728206-27729210 | 1 |
| chr21:30239400-30240399 | 1 |
| chr21:30241132-30242065 | 1 |
| chr21:33629001-33629807 | 1 |
| chr3:106390440-106391957 | 1 |
| chr3:106393263-106393659 | 1 |
| chr3:106543418-106544158 | 1 |
| chr3:107675691-107676878 | 1 |
| chr3:174008862-174009321 | 1 |
| chr3:178732434-178733838 | 1 |
| chr3:189907537-189908299 | 1 |
| chr3:34642533-34644446 | 1 |
| chr3:34670836-34672575 | 1 |
| chr3:37240480-37240740 | 1 |
| chr3:37254497-37254836 | 1 |
| chr3:50625949-50626261 | 1 |
| chr4:106248797-106249911 | 1 |
| chr4:110256866-110258688 | 1 |
| chr4:156123134-156124734 | 1 |
| chr4:16357169-16357203 | 1 |
| chr4:164170032-164170924 | 1 |
| chr4:17795006-17796679 | 1 |
| chr4:181859504-181860703 | 1 |
| chr4:182253785-182254815 | 1 |
| chr4:74871335-74873097 | 1 |
| chr4:77864252-77864744 | 1 |
| chr5:119458100-119459253 | 1 |
| chr5:147605275-147606051 | 1 |
| chr5:150873344-150873758 | 1 |
| chr5:34153894-34154866 | 1 |
| chr5:44684631-44685233 | 1 |
| chr5:56964532-56965155 | 1 |
| chr5:56988302-56989410 | 1 |
| chr5:68026547-68026771 | 1 |
| chr6:106629855-106630366 | 1 |
| chr6:120066176-120066660 | 1 |
| chr6:120155765-120156622 | 1 |
| chr6:120211055-120212346 | 1 |
| chr6:142142002-142145084 | 1 |
| chr6:142368912-142369796 | 1 |
| chr6:143668047-143669815 | 1 |
| chr6:58776280-58777091 | 1 |
| chr6:58777244-58778438 | 1 |
| chr6:58778631-58779245 | 1 |
| chr6:62291574-62293793 | 1 |
| chr6:62308410-62308765 | 1 |
| chr6:80254587-80255486 | 1 |
| chr6:81134610-81134899 | 1 |
| chr6:86361642-86362252 | 1 |
| chr6:87540239-87541149 | 1 |
| chr7:11211603-11211773 | 1 |
| chr7:24484131-24485105 | 1 |
| chr8:142735764-142735929 | 1 |
| chr9:107505665-107506265 | 1 |
| chr9:114790542-114790702 | 1 |
| chr9:114790932-114791388 | 1 |
| chr9:114792518-114792641 | 1 |
| chr9:140022067-140022152 | 1 |
| chr9:140023271-140023604 | 1 |
| chr9:140023869-140024391 | 1 |
| chr9:20339476-20340831 | 1 |
| chr9:28976664-28977134 | 1 |
| chr9:29077166-29078710 | 1 |
| chr9:5596043-5596612 | 1 |
| chr9:69015263-69015601 | 1 |
| chr9:74473566-74474381 | 1 |
| CHRD | 1 |
| CHRM3 | 1 |
| CHRM4 | 1 |
| CHRNA1 | 1 |
| CHRNA10 | 1 |
| CHRNA6 | 1 |
| CHRNA7 | 1 |
| CHRNE | 1 |
| chrX:140907324-140908048 | 1 |
| chrX:57927530-57927661 | 1 |
| chrX:74906843-74908910 | 1 |
| chrX:75116830-75118105 | 1 |
| chrY:14281244-14281880 | 1 |
| CHST10 | 1 |
| CHST2 | 1 |
| CHST3 | 1 |
| CHST8 | 1 |
| CHSY1 | 1 |
| CHURC1 | 1 |
| CIART | 1 |
| CICE | 1 |
| CIDEA | 1 |
| CIDEB | 1 |
| CIDECP | 1 |
| CII orfi | 1 |
| CINP | 1 |
| CIOorf136 | 1 |
| CIP29 | 1 |
| CISD1 | 1 |
| CIZ1 | 1 |
| CKAP4 | 1 |
| CKAP5 | 1 |
| CKB | 1 |
| CKMT2 | 1 |
| CKS2 | 1 |
| Cl I orf30 | 1 |
| Cl lorflO | 1 |
| CLASP1 | 1 |
| CLCF1 | 1 |
| CLCN3 | 1 |
| CLCN6 | 1 |
| CLCN7 | 1 |
| CLDN11 | 1 |
| CLDN12 | 1 |
| CLDN18 | 1 |
| CLDN20 | 1 |
| CLDN22 | 1 |
| CLDN5 | 1 |
| CLDN7 | 1 |
| CLDN9 | 1 |
| CLEC12A | 1 |
| CLEC14A | 1 |
| CLEC17A | 1 |
| CLEC1A | 1 |
| CLEC2B | 1 |
| CLEC2L | 1 |
| CLEC4A | 1 |
| CLEC4E | 1 |
| CLIC1 | 1 |
| CLIC2 | 1 |
| CLIC4 | 1 |
| CLIC6 | 1 |
| CLIP3 | 1 |
| CLK2 | 1 |
| CLN6 | 1 |
| CLNK | 1 |
| CLNS1A | 1 |
| CLPTM1L | 1 |
| CLSTN1 | 1 |
| CLSTN3 | 1 |
| CLTC | 1 |
| CLTCL1 | 1 |
| CLU | 1 |
| CLUAP1 | 1 |
| CMIP | 1 |
| CMPK1 | 1 |
| CMPK2 | 1 |
| CMSS1 | 1 |
| CMTM2 | 1 |
| CMTM3 | 1 |
| CMTM6 | 1 |
| CMTR1 | 1 |
| CNDP1 | 1 |
| CNGB3 | 1 |
| CNGBI | 1 |
| CNIH | 1 |
| CNIH4 | 1 |
| CNKSR2 | 1 |
| CNKSRI | 1 |
| CNN1 | 1 |
| CNN3 | 1 |
| CNOT10 | 1 |
| CNOT6 | 1 |
| CNOT6L | 1 |
| CNOT8 | 1 |
| CNP | 1 |
| CNPY3 | 1 |
| CNTD2 | 1 |
| CNTFR | 1 |
| CNTN1 | 1 |
| CNTN3 | 1 |
| COASY | 1 |
| COBL | 1 |
| COBRA1 | 1 |
| COBRAI | 1 |
| coc6 | 1 |
| COCH | 1 |
| COG1 | 1 |
| COG2 | 1 |
| COG3 | 1 |
| COG8 | 1 |
| COL16A1 | 1 |
| COL18A1 | 1 |
| COL1A1 | 1 |
| COL21A1 | 1 |
| COL24A1 | 1 |
| COL5A1 | 1 |
| COL5A3 | 1 |
| COL6A1 | 1 |
| COL6A2 | 1 |
| COL6A6 | 1 |
| COL7A1 | 1 |
| COL8A2 | 1 |
| COL9A1 | 1 |
| COLPH2 | 1 |
| COLQ | 1 |
| COMMD3 | 1 |
| COMMD4 | 1 |
| COMMD9 | 1 |
| COMT | 1 |
| COPB1 | 1 |
| COPB2 | 1 |
| COPG1 | 1 |
| COPS4 | 1 |
| COPS5 | 1 |
| COPS8 | 1 |
| COQ10A | 1 |
| COQ10B | 1 |
| COQ4 | 1 |
| COQ5 | 1 |
| COQ6 | 1 |
| CORO1C | 1 |
| CORO6 | 1 |
| CORT | 1 |
| COX10-AS1 | 1 |
| COX17 | 1 |
| COX19 | 1 |
| COX4I1 | 1 |
| COX5B | 1 |
| COX6A1 | 1 |
| COX6B1 | 1 |
| COX7A2L | 1 |
| CPB2 | 1 |
| CPEB1-AS1 | 1 |
| CPEB3 | 1 |
| CPLX3 | 1 |
| CPNE3 | 1 |
| CPNE7 | 1 |
| CPNE8 | 1 |
| CPNE9 | 1 |
| CPSF1 | 1 |
| CPSF6 | 1 |
| CPSN2 | 1 |
| CPT1C | 1 |
| CPT2 | 1 |
| CPZ | 1 |
| CR1 | 1 |
| CR2 | 1 |
| CRABP2 | 1 |
| CRBN | 1 |
| CREB1 | 1 |
| CREB3 | 1 |
| CREB3L2 | 1 |
| CREB5 | 1 |
| CREBRF | 1 |
| CREBZF | 1 |
| CREG1 | 1 |
| CREG2 | 1 |
| CRELD2 | 1 |
| CRHBP | 1 |
| CRIP1 | 1 |
| CRIP2 | 1 |
| CRIP3 | 1 |
| CRISP2 | 1 |
| CRK | 1 |
| CRKL | 1 |
| CRKRS | 1 |
| CRLF1 | 1 |
| CRLS1 | 1 |
| CROP | 1 |
| CROT | 1 |
| CRTAP | 1 |
| CRTC2 | 1 |
| CRYBA4 | 1 |
| CRYGS | 1 |
| CRYM | 1 |
| CRYZ | 1 |
| cs | 1 |
| CSDA | 1 |
| CSDE1 | 1 |
| CSF1 | 1 |
| CSF1R | 1 |
| CSF2RA | 1 |
| CSK | 1 |
| CSNK1G1 | 1 |
| CSNK1G2 | 1 |
| CSNK2A1 | 1 |
| CSRNP1 | 1 |
| CSRP1 | 1 |
| CSRP2BP | 1 |
| CSTB | 1 |
| CSTF2T | 1 |
| CTA-384D8.34 | 1 |
| CTA-384D8.35 | 1 |
| CTB-109A12.1 | 1 |
| CTBP1 | 1 |
| CTC-471F3.5 | 1 |
| CTC-498J12.3 | 1 |
| CTCF | 1 |
| CTD-2026C7.1 | 1 |
| CTD-2175A23.1 | 1 |
| CTD-2240E14.4 | 1 |
| CTD-2256P15.4 | 1 |
| CTD-2369P2.8 | 1 |
| CTD-2531D15.5 | 1 |
| CTD-3065J16.6 | 1 |
| CTD-3128G10.6 | 1 |
| CTDSP2 | 1 |
| CTNNA3 | 1 |
| CTNNB1 | 1 |
| CTNNBIP1 | 1 |
| CTNS | 1 |
| CTPS1 | 1 |
| CTR9 | 1 |
| CTRL | 1 |
| CTSA | 1 |
| CTSB | 1 |
| CTSC | 1 |
| CTSH | 1 |
| CTTNBP2 | 1 |
| CTU2 | 1 |
| CUGBP2 | 1 |
| CUL2 | 1 |
| CUL4A | 1 |
| CUL4B | 1 |
| CUL7 | 1 |
| CUX2 | 1 |
| CWF19L1 | 1 |
| CXCL1 | 1 |
| CXCL14 | 1 |
| CXCL16 | 1 |
| CXCL9 | 1 |
| CXCR1 | 1 |
| CXCR4 | 1 |
| CXCR5 | 1 |
| CXorf38 | 1 |
| CXXC4 | 1 |
| CYB561 | 1 |
| CYB561D2 | 1 |
| CYB5B | 1 |
| CYB5R2 | 1 |
| CYB5R3 | 1 |
| CYB5R4 | 1 |
| CYBASC3 | 1 |
| cycs | 1 |
| CYP17A1-AS1 | 1 |
| CYP1B1 | 1 |
| CYP26B1 | 1 |
| CYP27A1 | 1 |
| CYP2D7P1 | 1 |
| CYP2J2 | 1 |
| CYP2U1 | 1 |
| CYP3A4 | 1 |
| CYP4F24P | 1 |
| CYS1 | 1 |
| CYSLTR1 | 1 |
| CYSTM1 | 1 |
| DAAM1 | 1 |
| DAAM2 | 1 |
| DAB1 | 1 |
| DAB21P | 1 |
| DAGLA | 1 |
| DAK | 1 |
| DALRD3 | 1 |
| DAPK1-IT1 | 1 |
| DARC | 1 |
| DAZAP1 | 1 |
| DBN1 | 1 |
| DBNDD2 | 1 |
| DBNL | 1 |
| DBP | 1 |
| DCC | 1 |
| DCI | 1 |
| DCK | 1 |
| DCLK1 | 1 |
| DCLK2 | 1 |
| DCTN1 | 1 |
| DCTN2 | 1 |
| DCTN3 | 1 |
| DCUN1D2 | 1 |
| DCX | 1 |
| DCXR | 1 |
| DDN | 1 |
| DDOST | 1 |
| DDR1 | 1 |
| DDX11 | 1 |
| DDX17 | 1 |
| DDX21 | 1 |
| DDX24 | 1 |
| DDX31 | 1 |
| DDX47 | 1 |
| DDX54 | 1 |
| DDX55 | 1 |
| DDX58 | 1 |
| DDX59 | 1 |
| DDX6 | 1 |
| DDX60 | 1 |
| DDX60L | 1 |
| DEAF1 | 1 |
| DECR2 | 1 |
| DEDD2 | 1 |
| DEF8 | 1 |
| DEFA1B | 1 |
| DEFA4 | 1 |
| DEFB1 | 1 |
| DEFB131 | 1 |
| DEK | 1 |
| DENND1B | 1 |
| DENND2C | 1 |
| DENND2D | 1 |
| DENND4A | 1 |
| DENND4B | 1 |
| DENND5B | 1 |
| DENND6B | 1 |
| DENR | 1 |
| DEPDC4 | 1 |
| DEPDC5 | 1 |
| DERL1 | 1 |
| DERL3 | 1 |
| DFNB31 | 1 |
| DGCR14 | 1 |
| DGCR2 | 1 |
| DGCR6 | 1 |
| DGCR6L | 1 |
| DGCR8 | 1 |
| DGKE | 1 |
| DGKI | 1 |
| DGKZ | 1 |
| DGUOK | 1 |
| DHDDS | 1 |
| DHPS | 1 |
| DHRS13 | 1 |
| DHRS2 | 1 |
| DHRS4 | 1 |
| DHRS4L2 | 1 |
| DHRS7C | 1 |
| DHX30 | 1 |
| DHX34 | 1 |
| DHX36 | 1 |
| DHX40 | 1 |
| DHX9 | 1 |
| DIAPH1 | 1 |
| DICER1 | 1 |
| DICER1-AS | 1 |
| DIMT1 | 1 |
| DIP2B | 1 |
| DIRAS2 | 1 |
| DKFZP434A0131 | 1 |
| DKFZp686I15217 | 1 |
| DLEC1 | 1 |
| DLG1 | 1 |
| DLGAP1-AS4 | 1 |
| DLGAP1-AS5 | 1 |
| DLGAP4 | 1 |
| DLL1 | 1 |
| DLL4 | 1 |
| DMC1 | 1 |
| DMD | 1 |
| DMPK | 1 |
| DNA2 | 1 |
| DNAH12 | 1 |
| DNAI1 | 1 |
| DNAI2 | 1 |
| DNAJA2 | 1 |
| DNAJB11 | 1 |
| DNAJB14 | 1 |
| DNAJB4 | 1 |
| DNAJB6 | 1 |
| DNAJB9 | 1 |
| DNAJC11 | 1 |
| DNAJC3 | 1 |
| DNAJC3-AS1 | 1 |
| DNAJC4 | 1 |
| DNAJC5G | 1 |
| DNAJC6 | 1 |
| DNAL4 | 1 |
| DNALI1 | 1 |
| DNCL1 | 1 |
| DNHL1 | 1 |
| DNM1 | 1 |
| DNM2 | 1 |
| DNM3 | 1 |
| DNPEP | 1 |
| DNTTIP2 | 1 |
| DOCK8 | 1 |
| DOCK9 | 1 |
| DOGO | 1 |
| DOK3 | 1 |
| DOLK | 1 |
| DOT1L | 1 |
| DPAGT1 | 1 |
| DPF2 | 1 |
| DPM1 | 1 |
| DPM2 | 1 |
| DPP10 | 1 |
| DPP7 | 1 |
| DPY30 | 1 |
| DPYD | 1 |
| DPYSL3 | 1 |
| DRD2 | 1 |
| DRD4 | 1 |
| DRD5 | 1 |
| DROSHA | 1 |
| DSE | 1 |
| DSEL | 1 |
| DST | 1 |
| DSTN | 1 |
| DSTYK | 1 |
| DTWD1 | 1 |
| DTX3 | 1 |
| DUS1L | 1 |
| DUS3L | 1 |
| DUSP12 | 1 |
| DUSP14 | 1 |
| DUSP16 | 1 |
| DUSP3 | 1 |
| DUSP5 | 1 |
| DYNC1H1 | 1 |
| DYNLL1P1 | 1 |
| DYNLT3 | 1 |
| DYRK2 | 1 |
| E2F1 | 1 |
| E2F2 | 1 |
| E2F4 | 1 |
| E2F8 | 1 |
| E4F1 | 1 |
| EBAG9 | 1 |
| EBI3 | 1 |
| EBP | 1 |
| ECD | 1 |
| ECE1 | 1 |
| ECEL1 | 1 |
| ECHDC2 | 1 |
| ECHDC3 | 1 |
| ECI1 | 1 |
| ECI2 | 1 |
| ECSCR | 1 |
| EDEM1 | 1 |
| EDF1 | 1 |
| EDIL3 | 1 |
| EDN3 | 1 |
| EEAI | 1 |
| EED | 1 |
| EEF1A1P5 | 1 |
| EEF1AL7 | 1 |
| EEF1B2 | 1 |
| EEF1DP2 | 1 |
| EEFID | 1 |
| EEFSEC | 1 |
| EFCAB2 | 1 |
| EFCAB3 | 1 |
| EFHA1 | 1 |
| EFHA2 | 1 |
| EFNB3 | 1 |
| EFS | 1 |
| EGR4 | 1 |
| EHBP1 | 1 |
| EHD1 | 1 |
| EHF | 1 |
| EIF1B | 1 |
| EIF2A | 1 |
| EIF2AK3 | 1 |
| EIF2AK4 | 1 |
| EIF2B5 | 1 |
| EIF2C1 | 1 |
| EIF2S1 | 1 |
| EIF3E | 1 |
| EIF3H | 1 |
| EIF3S1 | 1 |
| EIF4A2 | 1 |
| EIF4BP6 | 1 |
| EIF4E3 | 1 |
| EIF4EBP1 | 1 |
| EIF4ENIF1 | 1 |
| EIF4G1 | 1 |
| EIF4G3 | 1 |
| EIF5A | 1 |
| ELAVL1 | 1 |
| ELAVL2 | 1 |
| ELAVL4 | 1 |
| ELF2 | 1 |
| ELK1 | 1 |
| ELK4 | 1 |
| ELL | 1 |
| ELL2 | 1 |
| ELMO1 | 1 |
| ELMO3 | 1 |
| ELMOD1 | 1 |
| ELMOD2 | 1 |
| ELOVL7 | 1 |
| ELP5 | 1 |
| ELTD1 | 1 |
| EMBP1 | 1 |
| EMC3 | 1 |
| EMID1 | 1 |
| EMILIN2 | 1 |
| EMILIN3 | 1 |
| EML3 | 1 |
| EMP1 | 1 |
| EMP3 | 1 |
| EMR2 | 1 |
| EMR3 | 1 |
| EN02 | 1 |
| ENAH | 1 |
| ENC1 | 1 |
| ENDOD1 | 1 |
| ENG | 1 |
| ENHO | 1 |
| ENKD1 | 1 |
| ENO3 | 1 |
| ENOPH1 | 1 |
| ENOSF1 | 1 |
| ENPP2 | 1 |
| ENPP4 | 1 |
| ENPP5 | 1 |
| ENTPD1 | 1 |
| ENTPD2 | 1 |
| ENTPD4 | 1 |
| ENY2 | 1 |
| EOMES | 1 |
| EPAS1 | 1 |
| EPB41L2 | 1 |
| EPB41L5 | 1 |
| EPB49 | 1 |
| EPG5 | 1 |
| EPHA2 | 1 |
| EPHA5 | 1 |
| EPHA6 | 1 |
| EPHB3 | 1 |
| EPN1 | 1 |
| EPN3 | 1 |
| EPS8L2 | 1 |
| EPSTI1 | 1 |
| EPT1 | 1 |
| ERC2 | 1 |
| ERCC3 | 1 |
| ERG | 1 |
| ERGIC1 | 1 |
| ERGIC3 | 1 |
| ERI1 | 1 |
| ERI3 | 1 |
| ERLEC1 | 1 |
| ERN1 | 1 |
| ERP29 | 1 |
| ERP44 | 1 |
| ESAM | 1 |
| ESD | 1 |
| ESR1 | 1 |
| ESRG | 1 |
| ESYT1 | 1 |
| ESYT3 | 1 |
| ETF1P2 | 1 |
| ETFB | 1 |
| ETV3 | 1 |
| ETV4 | 1 |
| ETV6 | 1 |
| ETV7 | 1 |
| EVC | 1 |
| EVI2B | 1 |
| EVL | 1 |
| EWSR1 | 1 |
| EXOSC3 | 1 |
| EXOSC5 | 1 |
| EXOSC9 | 1 |
| EXPH5 | 1 |
| EXTL2 | 1 |
| EYA3 | 1 |
| F11R | 1 |
| F13A1 | 1 |
| F2R | 1 |
| F3 | 1 |
| F5 | 1 |
| F7 | 1 |
| FA2H | 1 |
| FAAP20 | 1 |
| FABP3 | 1 |
| FADS1 | 1 |
| FAH | 1 |
| FAHD1 | 1 |
| FAHD2A | 1 |
| FAM100B | 1 |
| FAM102B | 1 |
| FAM103A1 | 1 |
| FAM107A | 1 |
| FAM110A | 1 |
| FAM111A | 1 |
| FAM114A2 | 1 |
| FAM115C | 1 |
| FAM116A | 1 |
| FAM116B | 1 |
| FAM117A | 1 |
| FAM117B | 1 |
| FAM118A | 1 |
| FAM122A | 1 |
| FAM122B | 1 |
| FAM124B | 1 |
| FAM127A | 1 |
| FAM127C | 1 |
| FAM129B | 1 |
| FAM131B | 1 |
| FAM134A | 1 |
| FAM134B | 1 |
| FAM134C | 1 |
| FAM135A | 1 |
| FAM13B | 1 |
| FAM149A | 1 |
| FAM152A | 1 |
| FAM152B | 1 |
| FAM153B | 1 |
| FAM154A | 1 |
| FAM160B1 | 1 |
| FAM163A | 1 |
| FAM163B | 1 |
| FAM164A | 1 |
| FAM166A | 1 |
| FAM168A | 1 |
| FAM176B | 1 |
| FAM178A | 1 |
| FAM183A | 1 |
| FAM184B | 1 |
| FAM189A1 | 1 |
| FAM18B2 | 1 |
| FAM193B | 1 |
| FAM195A | 1 |
| FAM19A2 | 1 |
| FAM19A5 | 1 |
| FAM200A | 1 |
| FAM205A | 1 |
| FAM211A | 1 |
| FAM211B | 1 |
| FAM212A | 1 |
| FAM214A | 1 |
| FAM21C | 1 |
| FAM32B | 1 |
| FAM38A | 1 |
| FAM40B | 1 |
| FAM43A | 1 |
| FAM45A | 1 |
| FAM46C | 1 |
| FAM48A | 1 |
| FAM48B1 | 1 |
| FAM49B | 1 |
| FAM53B | 1 |
| FAM55A | 1 |
| FAM58A | 1 |
| FAM59A | 1 |
| FAM5B | 1 |
| FAM60A | 1 |
| FAM63A | 1 |
| FAM63B | 1 |
| FAM65A | 1 |
| FAM65B | 1 |
| FAM69B | 1 |
| FAM70A | 1 |
| FAM71C | 1 |
| FAM71E1 | 1 |
| FAM78A | 1 |
| FAM78B | 1 |
| FAM81A | 1 |
| FAM83F | 1 |
| FAM83H | 1 |
| FAM84B | 1 |
| FAM85B | 1 |
| FAM86C2P | 1 |
| FAM86HP | 1 |
| FAM87B | 1 |
| FAM89A | 1 |
| FAM89B | 1 |
| FAM96A | 1 |
| FAM98A | 1 |
| FANCL | 1 |
| FAR1 | 1 |
| FAS | 1 |
| FASN | 1 |
| FASTKD2 | 1 |
| FASTKD5 | 1 |
| FBLIM1 | 1 |
| FBLN2 | 1 |
| FBLN5 | 1 |
| FBLN7 | 1 |
| FBN3 | 1 |
| FBP1 | 1 |
| FBRS | 1 |
| FBXL14 | 1 |
| FBXL15 | 1 |
| FBXL2 | 1 |
| FBXL22 | 1 |
| FBXL5 | 1 |
| FBXO11 | 1 |
| FBXO2 | 1 |
| FBXO22 | 1 |
| FBXO3 | 1 |
| FBXO46 | 1 |
| FBXO5 | 1 |
| FBXO6 | 1 |
| FBXO7 | 1 |
| FBXW4 | 1 |
| FBXW5 | 1 |
| FBXW7 | 1 |
| FBXW9 | 1 |
| FCAR | 1 |
| FCER1G | 1 |
| FCER2 | 1 |
| FCGR1B | 1 |
| FCGR3A | 1 |
| FCGR3B | 1 |
| FCGRT | 1 |
| FCN3 | 1 |
| FCRL2 | 1 |
| FCRL6 | 1 |
| FCRLA | 1 |
| FEM1A | 1 |
| FEM1B | 1 |
| FEZ1 | 1 |
| FEZ2 | 1 |
| FEZF2 | 1 |
| FGD3 | 1 |
| FGD4 | 1 |
| FGF1 | 1 |
| FGF12 | 1 |
| FGF14-AS1 | 1 |
| FGF16 | 1 |
| FGF9 | 1 |
| FGFBP2 | 1 |
| FGFR1 | 1 |
| FGFR3 | 1 |
| FGL2 | 1 |
| FHL2 | 1 |
| FICD | 1 |
| FIGNL1 | 1 |
| FILIP1L | 1 |
| FIS1 | 1 |
| FKBP10 | 1 |
| FKBP11 | 1 |
| FKBP14 | 1 |
| FKBP2 | 1 |
| FKBP4 | 1 |
| FKBP5 | 1 |
| FKBPL | 1 |
| FKRP | 1 |
| FLJ00312 | 1 |
| FLJ12078 | 1 |
| FLJ20021 | 1 |
| FLJ20699 | 1 |
| FLJ21438 | 1 |
| FLJ25363 | 1 |
| FLJ31306 | 1 |
| FLJ31813 | 1 |
| FLJ32063 | 1 |
| FLJ35390 | 1 |
| FLJ40473 | 1 |
| FLJ43390 | 1 |
| FLJ43860 | 1 |
| FLJ43987 | 1 |
| FLJ45337 | 1 |
| FLJ45340 | 1 |
| FLNB | 1 |
| FLOT2 | 1 |
| FLT1 | 1 |
| FLVCR2 | 1 |
| FLYWCH2 | 1 |
| FMNL3 | 1 |
| FMO5 | 1 |
| FMR1 | 1 |
| FN1 | 1 |
| FNBP1L | 1 |
| FNBP4 | 1 |
| FNDC3A | 1 |
| FNDC3B | 1 |
| FNIP1 | 1 |
| FNIP2 | 1 |
| FOLR3 | 1 |
| FOXC1 | 1 |
| FOXF1 | 1 |
| FOXJ2 | 1 |
| FOXJ3 | 1 |
| FOXO1 | 1 |
| FOXO3 | 1 |
| FOXP1 | 1 |
| FPGS | 1 |
| FPR2 | 1 |
| FRAT1 | 1 |
| FRMD4B | 1 |
| FRMD5 | 1 |
| FRMPD4 | 1 |
| FRS2 | 1 |
| FSCN2 | 1 |
| FSD1L | 1 |
| FSD2 | 1 |
| FSTL5 | 1 |
| FTH1P15 | 1 |
| FTH1P3 | 1 |
| FTH1P8 | 1 |
| FTHL11 | 1 |
| FTHL2 | 1 |
| FTHL8 | 1 |
| FTL | 1 |
| FTSJ2 | 1 |
| FUBP1 | 1 |
| FUCA1 | 1 |
| FUNDC2P3 | 1 |
| FURIN | 1 |
| FXR2 | 1 |
| FXYD1 | 1 |
| FXYD5 | 1 |
| FYTTD1 | 1 |
| FZD4 | 1 |
| FZD5 | 1 |
| FZD7 | 1 |
| G3BP1 | 1 |
| G3BP2 | 1 |
| G6PC3 | 1 |
| G6PD | 1 |
| GAB2 | 1 |
| GABARAPL1 | 1 |
| GABARAPL2 | 1 |
| GABPA | 1 |
| GABPAP | 1 |
| GABRA 1 | 1 |
| GABRA3 | 1 |
| GABRG1 | 1 |
| GABRG2 | 1 |
| GABRQ | 1 |
| GAD1 | 1 |
| GADD45GIP1 | 1 |
| GAGE12I | 1 |
| GALC | 1 |
| GALE | 1 |
| GALNT10 | 1 |
| GALNT14 | 1 |
| GALNT9 | 1 |
| GALNTL1 | 1 |
| GALNTL5 | 1 |
| GALNTL6 | 1 |
| GAP43 | 1 |
| GAPDH | 1 |
| GAPDHP16 | 1 |
| GAPDHP42 | 1 |
| GAPDHP46 | 1 |
| GAPDHP51 | 1 |
| GAPDHP71 | 1 |
| GAPDHP72 | 1 |
| GAPT | 1 |
| GAPVD1 | 1 |
| GART | 1 |
| GATAD1 | 1 |
| GBA | 1 |
| GBE1 | 1 |
| GCA | 1 |
| GCDH | 1 |
| GCFC1 | 1 |
| GCN1L1 | 1 |
| GCSH | 1 |
| GDF10 | 1 |
| GDF15 | 1 |
| GDF5 | 1 |
| GDPD3 | 1 |
| GEMIN5 | 1 |
| GEMIN8 | 1 |
| GFAP | 1 |
| GFI1 | 1 |
| GFOD2 | 1 |
| GFPT1 | 1 |
| GGACT | 1 |
| GGPS1 | 1 |
| GHR | 1 |
| GID4 | 1 |
| GID8 | 1 |
| GIMAP1 | 1 |
| GIMAP5 | 1 |
| GIMAP6 | 1 |
| GINS2 | 1 |
| GIPC3 | 1 |
| GIT1 | 1 |
| GIT2 | 1 |
| GJA3 | 1 |
| GJA5 | 1 |
| GJB2 | 1 |
| GK5 | 1 |
| GLA | 1 |
| GLCCI1 | 1 |
| GLCE | 1 |
| GLIPR2 | 1 |
| GLIS2 | 1 |
| GLIS3 | 1 |
| GLRA2 | 1 |
| GLRA3 | 1 |
| GLRX | 1 |
| GLRX2 | 1 |
| GLS | 1 |
| GLT25D1 | 1 |
| GLTPD1 | 1 |
| GLUL | 1 |
| GMEB1 | 1 |
| GMFB | 1 |
| GMIP | 1 |
| GMPR | 1 |
| GMPR2 | 1 |
| GNA11 | 1 |
| GNA13 | 1 |
| GNAL | 1 |
| GNAS | 1 |
| GNB1L | 1 |
| GNB4 | 1 |
| GNE | 1 |
| GNG10 | 1 |
| GNG13 | 1 |
| GNG3 | 1 |
| GNG5 | 1 |
| GNL3 | 1 |
| GNPDA2 | 1 |
| GNPTAB | 1 |
| GNPTG | 1 |
| GNRH1 | 1 |
| GNRHR | 1 |
| GNS | 1 |
| GOLCAI | 1 |
| GOLGA2P5 | 1 |
| GOLGA6L5P | 1 |
| GOLGA6L9 | 1 |
| GOLPH3 | 1 |
| GOLPH3L | 1 |
| GOSR1 | 1 |
| GP1BB | 1 |
| GP9 | 1 |
| GPATCH11 | 1 |
| GPATCH2L | 1 |
| GPBP1 | 1 |
| GPC1 | 1 |
| GPC5 | 1 |
| GPER | 1 |
| GPHA2 | 1 |
| GPHN | 1 |
| GPN3 | 1 |
| GPR1 | 1 |
| GPR12 | 1 |
| GPR124 | 1 |
| GPR132 | 1 |
| GPR137 | 1 |
| GPR137C | 1 |
| GPR160 | 1 |
| GPR175 | 1 |
| GPR176 | 1 |
| GPR179 | 1 |
| GPR18 | 1 |
| GPR21 | 1 |
| GPR34 | 1 |
| GPR35 | 1 |
| GPR37L1 | 1 |
| GPR4 | 1 |
| GPR52 | 1 |
| GPR61 | 1 |
| GPR65 | 1 |
| GPR79 | 1 |
| GPR83 | 1 |
| GPR84 | 1 |
| GPR89A | 1 |
| GPR89B | 1 |
| GPR89C | 1 |
| GPRC5C | 1 |
| GPRIN1 | 1 |
| GPRIN3 | 1 |
| GPS1 | 1 |
| GPS2 | 1 |
| GPSM1 | 1 |
| GPX1 | 1 |
| GRAMD4 | 1 |
| GRHPR | 1 |
| GRIA1 | 1 |
| GRIA2 | 1 |
| GRID1 | 1 |
| GRID2 | 1 |
| GRIK1-AS1 | 1 |
| GRIK2 | 1 |
| GRIK3 | 1 |
| GRIN3B | 1 |
| GRIP1 | 1 |
| GRK6 | 1 |
| GRN | 1 |
| GRPEL1 | 1 |
| GSDMB | 1 |
| GSG2 | 1 |
| GSS | 1 |
| GSTA4 | 1 |
| GSTCD | 1 |
| GSTP1 | 1 |
| GTF2F2 | 1 |
| GTF2H3 | 1 |
| GTF2IRD2B | 1 |
| GTF3C4 | 1 |
| GTF3C6 | 1 |
| GTPBP4 | 1 |
| GUCD1 | 1 |
| GUCY1A3 | 1 |
| GUSBP11 | 1 |
| GXYLT1 | 1 |
| GXYLT2 | 1 |
| GYPC | 1 |
| GZF1 | 1 |
| GZMA | 1 |
| GZMB | 1 |
| GZMM | 1 |
| H1F0 | 1 |
| H1FX | 1 |
| H2AFX | 1 |
| H2AFY | 1 |
| H2AFY2 | 1 |
| HACL1 | 1 |
| HADH | 1 |
| HAGH | 1 |
| HAGHL | 1 |
| HAP1 | 1 |
| HAPLN4 | 1 |
| HAR1A | 1 |
| HARS2 | 1 |
| HAS3 | 1 |
| HAT1 | 1 |
| HAUS7 | 1 |
| HBG2 | 1 |
| HBP1 | 1 |
| HBXIP | 1 |
| HCCA2 | 1 |
| HCFC1 | 1 |
| HCG4P11 | 1 |
| HCRTR1 | 1 |
| HCST | 1 |
| HDAC2 | 1 |
| HDAC3 | 1 |
| HDAC6 | 1 |
| HDAC7 | 1 |
| HDAC7A | 1 |
| HDC | 1 |
| HEATR1 | 1 |
| HEATR2 | 1 |
| HEATR5B | 1 |
| HELZ | 1 |
| HELZ2 | 1 |
| HEPN1 | 1 |
| HERC2 | 1 |
| HERC2P2 | 1 |
| HERC2P3 | 1 |
| HERC5 | 1 |
| HERC6 | 1 |
| HERPUD1 | 1 |
| HES4 | 1 |
| HES6 | 1 |
| HEXB | 1 |
| HEXDC | 1 |
| HEY1 | 1 |
| HEY2 | 1 |
| HEYL | 1 |
| HGF | 1 |
| HGS | 1 |
| HGSNAT | 1 |
| HHIP | 1 |
| HIF3A | 1 |
| HIGD1A | 1 |
| HIGD1B | 1 |
| HIST1H2AD | 1 |
| HIST1H2AG | 1 |
| HIST1H2AH | 1 |
| HIST1H2AI | 1 |
| HIST1H2AJ | 1 |
| HIST1H2AK | 1 |
| HIST1H2AL | 1 |
| HIST1H2AM | 1 |
| HIST1H2BH | 1 |
| HIST1H2BJ | 1 |
| HIST1H2BK | 1 |
| HIST1H3F | 1 |
| HIST1H4C | 1 |
| HIST1H4K | 1 |
| HIST2H3A | 1 |
| HIVEP1 | 1 |
| HLA-A29.1 | 1 |
| HLA-B | 1 |
| HLA-C | 1 |
| HLA-DOA | 1 |
| HLA-DPB1 | 1 |
| HLA-DQA1 | 1 |
| HLA-DRB6 | 1 |
| HLA-E | 1 |
| HLA-H | 1 |
| HLX | 1 |
| HM13-AS1 | 1 |
| HMG20B | 1 |
| HMGB1L1 | 1 |
| HMGB2 | 1 |
| HMGB3P9 | 1 |
| HMGCLL1 | 1 |
| HMGCS1 | 1 |
| HMGCS2 | 1 |
| HMGN2P8 | 1 |
| HMGN4 | 1 |
| HMGXB4 | 1 |
| HMHA1 | 1 |
| HMOX1 | 1 |
| HMX1 | 1 |
| HNMT | 1 |
| HNRNPA1 | 1 |
| HNRNPA1P21 | 1 |
| HNRNPA3 | 1 |
| HNRNPC | 1 |
| HNRNPD | 1 |
| HNRNPDL | 1 |
| HNRNPH1 | 1 |
| HNRNPH2 | 1 |
| HNRNPU | 1 |
| HNRNPU-AS1 | 1 |
| HNRNPUL2 | 1 |
| HNRPDL | 1 |
| HNRPH3 | 1 |
| HOMER1 | 1 |
| HOMER2 | 1 |
| HOPX | 1 |
| HOXB2 | 1 |
| HP1BP3 | 1 |
| HPCAL1 | 1 |
| HPCAL4 | 1 |
| HPRT1 | 1 |
| HPS4 | 1 |
| HPSE | 1 |
| HPSE2 | 1 |
| HRAS | 1 |
| HRASLS | 1 |
| HRCT1 | 1 |
| HS.120742 | 1 |
| HS.123471 | 1 |
| HS.126889 | 1 |
| HS.134166 | 1 |
| HS.134945 | 1 |
| HS.143292 | 1 |
| HS.145784 | 1 |
| HS.149804 | 1 |
| HS.156270 | 1 |
| HS.159070 | 1 |
| HS.197070 | 1 |
| HS.199486 | 1 |
| HS.200644 | 1 |
| HS.214218 | 1 |
| HS.278948 | 1 |
| HS.372683 | 1 |
| HS.376729 | 1 |
| HS.377894 | 1 |
| HS.400774 | 1 |
| HS.406106 | 1 |
| HS.407903 | 1 |
| HS.415336 | 1 |
| HS.417262 | 1 |
| HS.436662 | 1 |
| HS.481369 | 1 |
| HS.490862 | 1 |
| HS.500311 | 1 |
| HS.520349 | 1 |
| HS.523975 | 1 |
| HS.525436 | 1 |
| HS.533278 | 1 |
| HS.538300 | 1 |
| HS.538683 | 1 |
| HS.538893 | 1 |
| HS.538981 | 1 |
| HS.539427 | 1 |
| HS.539982 | 1 |
| HS.540116 | 1 |
| HS.540958 | 1 |
| HS.541221 | 1 |
| HS.541237 | 1 |
| HS.541315 | 1 |
| HS.541596 | 1 |
| HS.541858 | 1 |
| HS.542013 | 1 |
| HS.543219 | 1 |
| HS.543313 | 1 |
| HS.543452 | 1 |
| HS.543489 | 1 |
| HS.543629 | 1 |
| HS.544210 | 1 |
| HS.545068 | 1 |
| HS.551324 | 1 |
| HS.553437 | 1 |
| HS.557431 | 1 |
| HS.559008 | 1 |
| HS.559406 | 1 |
| HS.560343 | 1 |
| HS.560605 | 1 |
| HS.561385 | 1 |
| HS.561625 | 1 |
| HS.561844 | 1 |
| HS.561954 | 1 |
| HS.562543 | 1 |
| HS.562701 | 1 |
| HS.564131 | 1 |
| HS.564153 | 1 |
| HS.564718 | 1 |
| HS.564723 | 1 |
| HS.564906 | 1 |
| HS.565658 | 1 |
| HS.566337 | 1 |
| HS.566487 | 1 |
| HS.566521 | 1 |
| HS.567137 | 1 |
| HS.568710 | 1 |
| HS.568866 | 1 |
| HS.570747 | 1 |
| HS.570950 | 1 |
| HS.571488 | 1 |
| HS.571795 | 1 |
| HS.574871 | 1 |
| HS.575187 | 1 |
| HS.575709 | 1 |
| HS.576801 | 1 |
| HS.577032 | 1 |
| HS.577923 | 1 |
| HS.577948 | 1 |
| HS.578332 | 1 |
| HS.578456 | 1 |
| HS.579434 | 1 |
| HS.580901 | 1 |
| HS.581906 | 1 |
| HS.582245 | 1 |
| HS.582536 | 1 |
| HS.583408 | 1 |
| HS1BP3 | 1 |
| HS3ST4 | 1 |
| HS6ST1 | 1 |
| HS6ST2 | 1 |
| HSBP1 | 1 |
| HSD11B1L | 1 |
| HSD17B1 | 1 |
| HSD3B7 | 1 |
| HSF4 | 1 |
| HSGT1 | 1 |
| HSH2D | 1 |
| HSN2 | 1 |
| HSP90AA4P | 1 |
| HSP90AB2P | 1 |
| HSPA12B | 1 |
| HSPA14 | 1 |
| HSPA1B | 1 |
| HSPA5 | 1 |
| HSPA7 | 1 |
| HSPA9 | 1 |
| HSPB2 | 1 |
| HSPB7 | 1 |
| HSPB9 | 1 |
| HSPBAP1 | 1 |
| HSPBPI | 1 |
| HSPC171 | 1 |
| HSPD1P10 | 1 |
| HSPD1P11 | 1 |
| HTR2A | 1 |
| HTR2C | 1 |
| HTR7 | 1 |
| HUNK | 1 |
| HUS1B | 1 |
| HYAL3 | 1 |
| HYDIN | 1 |
| IBTK | 1 |
| ICA1 | 1 |
| ICA1L | 1 |
| ICAM4 | 1 |
| ICAM5 | 1 |
| ICMT | 1 |
| ICOSLG | 1 |
| ICT1 | 1 |
| ID1 | 1 |
| ID2 | 1 |
| ID4 | 1 |
| IDH1 | 1 |
| IDH2 | 1 |
| IDH3A | 1 |
| IDH3B | 1 |
| IER3 | 1 |
| IER5L | 1 |
| IFFO1 | 1 |
| IFI16 | 1 |
| IFI30 | 1 |
| IFIH1 | 1 |
| IFIT1 | 1 |
| IFIT2 | 1 |
| IFIT5 | 1 |
| IFNA2 | 1 |
| IFNAR2 | 1 |
| IFNG | 1 |
| IFRD1 | 1 |
| IFT122 | 1 |
| IFT74 | 1 |
| IGF1 | 1 |
| IGF2 | 1 |
| IGF2BP2 | 1 |
| IGF2R | 1 |
| IGFBP3 | 1 |
| IGFBP6 | 1 |
| IGIP | 1 |
| IGLL1 | 1 |
| IGSF10 | 1 |
| IGSF11-AS1 | 1 |
| IGSF6 | 1 |
| IGSF9B | 1 |
| IKBKE | 1 |
| IKZF1 | 1 |
| IKZF5 | 1 |
| IL10RB-AS1 | 1 |
| IL11 | 1 |
| IL11RA | 1 |
| IL15 | 1 |
| IL17D | 1 |
| IL18BP | 1 |
| IL1R1 | 1 |
| IL1RAPL1 | 1 |
| IL27 | 1 |
| IL2RB | 1 |
| IL5RA | 1 |
| IL6R | 1 |
| IL8RB | 1 |
| ILF3 | 1 |
| IMAA | 1 |
| IMMP2L | 1 |
| IMP4 | 1 |
| IMPG2 | 1 |
| INF2 | 1 |
| INHBA-AS1 | 1 |
| INO80B | 1 |
| INO80D | 1 |
| INOC1 | 1 |
| INPP5F | 1 |
| INPP5K | 1 |
| INPPL1 | 1 |
| INSIG2 | 1 |
| INTS10 | 1 |
| INTS4 | 1 |
| INTS7 | 1 |
| INTU | 1 |
| IP6K1 | 1 |
| IPO] | 1 |
| IQCA1 | 1 |
| IQCJ | 1 |
| IQCK | 1 |
| IQSEC1 | 1 |
| IRAK1 | 1 |
| IREB2 | 1 |
| IRF2BP2 | 1 |
| IRF2BPL | 1 |
| IRF3 | 1 |
| IRF6 | 1 |
| IRF8 | 1 |
| IRX1 | 1 |
| ISCA2 | 1 |
| ISCU | 1 |
| ISG20 | 1 |
| ISL2 | 1 |
| ISLR | 1 |
| ISLR2 | 1 |
| ISOC1 | 1 |
| ISYNA1 | 1 |
| ITFG1 | 1 |
| ITFGI | 1 |
| ITGA1 | 1 |
| ITGA4 | 1 |
| ITGAE | 1 |
| ITGAV | 1 |
| ITGAX | 1 |
| ITGB4 | 1 |
| ITGB7 | 1 |
| ITGBL1 | 1 |
| ITIH2 | 1 |
| ITK | 1 |
| ITM2B | 1 |
| ITPA | 1 |
| ITPK1 | 1 |
| ITPKB | 1 |
| ITPRIP | 1 |
| IZUMO4 | 1 |
| JADE2 | 1 |
| JAG1 | 1 |
| JAK1 | 1 |
| JAM2 | 1 |
| JDP2 | 1 |
| JKAMP | 1 |
| JMJD4 | 1 |
| JMJD8 | 1 |
| JOSD2 | 1 |
| JPH2 | 1 |
| JPH4 | 1 |
| JRKL | 1 |
| JUN | 1 |
| KAL1 | 1 |
| KANSL3 | 1 |
| KARS | 1 |
| KAT2B | 1 |
| KAT5 | 1 |
| KATNA1 | 1 |
| KATNB1 | 1 |
| KAZALD1 | 1 |
| KBTBD11 | 1 |
| KBTBD2 | 1 |
| KBTBD7 | 1 |
| KCNA1 | 1 |
| KCNAB2 | 1 |
| KCNAB3 | 1 |
| KCNC2 | 1 |
| KCNF1 | 1 |
| KCNH3 | 1 |
| KCNH6 | 1 |
| KCNH7 | 1 |
| KCNIP3 | 1 |
| KCNJ12 | 1 |
| KCNJ14 | 1 |
| KCNJ2 | 1 |
| KCNJ3 | 1 |
| KCNK1 | 1 |
| KCNK12 | 1 |
| KCNK3 | 1 |
| KCNK9 | 1 |
| KCNMB1 | 1 |
| KCNMB4 | 1 |
| KCNN1 | 1 |
| KCNN3 | 1 |
| KCNQ5-AS2 | 1 |
| KCNV1 | 1 |
| KCTD13 | 1 |
| KCTD17 | 1 |
| KCTD4 | 1 |
| KDELR1 | 1 |
| KDELR2 | 1 |
| KDM1B | 1 |
| KDM2B | 1 |
| KDM5A | 1 |
| KDM5B | 1 |
| KDM6A | 1 |
| KHDRBS2 | 1 |
| KHK | 1 |
| KIAA0182 | 1 |
| KIAA0256 | 1 |
| KIAA0261 | 1 |
| KIAA0423 | 1 |
| KIAA0513 | 1 |
| KIAA0564 | 1 |
| KIAA0888 | 1 |
| KIAA0895 | 1 |
| KIAA0907 | 1 |
| KIAA1024 | 1 |
| KIAA1212 | 1 |
| KIAA1219 | 1 |
| KIAA1239 | 1 |
| KIAA1267 | 1 |
| KIAA1324L | 1 |
| KIAA1328 | 1 |
| KIAA1370 | 1 |
| KIAA1377 | 1 |
| KIAA1466 | 1 |
| KIAA1539 | 1 |
| KIAA1598 | 1 |
| KIAA1600 | 1 |
| KIAA1632 | 1 |
| KIAA1683 | 1 |
| KIAA1712 | 1 |
| KIAA1841 | 1 |
| KIAA2018 | 1 |
| KIAA2026 | 1 |
| KIF13B | 1 |
| KIF19 | 1 |
| KIF20B | 1 |
| KIF22 | 1 |
| KIF2A | 1 |
| KIF9 | 1 |
| KIFC3 | 1 |
| KIR2DL3 | 1 |
| KISS1 | 1 |
| KLC1 | 1 |
| KLC2 | 1 |
| KLF12 | 1 |
| KLF13 | 1 |
| KLF15 | 1 |
| KLF2 | 1 |
| KLF4 | 1 |
| KLF5 | 1 |
| KLF7 | 1 |
| KLHDC2 | 1 |
| KLHDC3 | 1 |
| KLHDC8B | 1 |
| KLHDCI | 1 |
| KLHL22 | 1 |
| KLHL24 | 1 |
| KLHL25 | 1 |
| KLHL28 | 1 |
| KLHL34 | 1 |
| KLHL5 | 1 |
| KLHL6 | 1 |
| KLK3 | 1 |
| KLPI | 1 |
| KLRF1 | 1 |
| KLRK1 | 1 |
| KNDC1 | 1 |
| KNTC1 | 1 |
| KPNA6 | 1 |
| KPNAI | 1 |
| KPNB1 | 1 |
| KRBA1 | 1 |
| KRCC1 | 1 |
| KREMEN2 | 1 |
| KRIT1 | 1 |
| KRT18P10 | 1 |
| KRT18P12 | 1 |
| KRT222 | 1 |
| KRT23 | 1 |
| KRT40 | 1 |
| KRT6E | 1 |
| KRT83 | 1 |
| KRT8P46 | 1 |
| KRTCAP2 | 1 |
| KSR1 | 1 |
| L3HYPDH | 1 |
| LACC1 | 1 |
| LAGE3 | 1 |
| LAMA5 | 1 |
| LAMB2 | 1 |
| LAMP2 | 1 |
| LAMTOR1 | 1 |
| LANCL1 | 1 |
| LAP3 | 1 |
| LAPTM4B | 1 |
| LARP4 | 1 |
| LARP7 | 1 |
| LASS6 | 1 |
| LAT | 1 |
| LBA1 | 1 |
| LBH | 1 |
| LCA5L | 1 |
| LCAT | 1 |
| LCK | 1 |
| LCMT2 | 1 |
| LCN1 | 1 |
| LCN10 | 1 |
| LCNL1 | 1 |
| LCOR | 1 |
| LDB1 | 1 |
| LDHA | 1 |
| LDLR | 1 |
| LDLRAP1 | 1 |
| LEMD2 | 1 |
| LENG8 | 1 |
| LEPREL2 | 1 |
| LEPROTL1 | 1 |
| LFNG | 1 |
| LGALS1 | 1 |
| LGALS14 | 1 |
| LGALS2 | 1 |
| LGALS3 | 1 |
| LGI3 | 1 |
| LGI4 | 1 |
| LGICZI | 1 |
| LHPP | 1 |
| LHX4-AS1 | 1 |
| LHX8 | 1 |
| LILRA2 | 1 |
| LILRA3 | 1 |
| LILRB4 | 1 |
| LIMA1 | 1 |
| LIMCH1 | 1 |
| LIMD1 | 1 |
| LIMD1-AS1 | 1 |
| LIME1 | 1 |
| LIMK2 | 1 |
| LIMS2 | 1 |
| LIN7B | 1 |
| LIN7C | 1 |
| LINC00094 | 1 |
| LINC00158 | 1 |
| LINC00242 | 1 |
| LINC00263 | 1 |
| LINC00299 | 1 |
| LINC00309 | 1 |
| LINC00324 | 1 |
| LINC00612 | 1 |
| LINC00641 | 1 |
| LINC00645 | 1 |
| LINC00926 | 1 |
| LINC01107 | 1 |
| LINC01301 | 1 |
| LINC01388 | 1 |
| LINGO1 | 1 |
| LINGO2 | 1 |
| LIPH | 1 |
| LLPH | 1 |
| LMAN2L | 1 |
| LMBRDI | 1 |
| LMF1 | 1 |
| LMO7 | 1 |
| LMO7-AS1 | 1 |
| LMOD1 | 1 |
| LNP1 | 1 |
| LNX1 | 1 |
| LOC100128071 | 1 |
| LOC100129633 | 1 |
| LOC100130872 | 1 |
| LOC100133161 | 1 |
| LOC100216546 | 1 |
| LOC100329109 | 1 |
| LOC100505495 | 1 |
| LOC100505576 | 1 |
| LOC100506599 | 1 |
| LOC100507043 | 1 |
| LOC100507588 | 1 |
| LOC113230 | 1 |
| LOC126170 | 1 |
| LOC130074 | 1 |
| LOC131055 | 1 |
| LOC134997 | 1 |
| LOC136143 | 1 |
| LOC146517 | 1 |
| LOC151534 | 1 |
| LOC157627 | 1 |
| LOC158301 | 1 |
| LOC158376 | 1 |
| LOC162632 | 1 |
| LOC163131 | 1 |
| LOC219854 | 1 |
| LOC23117 | 1 |
| LOC253039 | 1 |
| LOC283174 | 1 |
| LOC283888 | 1 |
| LOC283901 | 1 |
| LOC284454 | 1 |
| LOC285074 | 1 |
| LOC285588 | 1 |
| LOC2B3523 | 1 |
| LOC338799 | 1 |
| LOC338817 | 1 |
| LOC340508 | 1 |
| LOC341315 | 1 |
| LOC346950 | 1 |
| LOC347376 | 1 |
| LOC34741 1 | 1 |
| LOC387790 | 1 |
| LOC387870 | 1 |
| LOC388122 | 1 |
| LOC388275 | 1 |
| LOC388344 | 1 |
| LOC388474 | 1 |
| LOC389179 | 1 |
| LOC389332 | 1 |
| LOC389672 | 1 |
| LOC390874 | 1 |
| LOC390933 | 1 |
| LOC391429 | 1 |
| LOC391739 | 1 |
| LOC391845 | 1 |
| LOC392364 | 1 |
| LOC400652 | 1 |
| LOC400986 | 1 |
| LOC401052 | 1 |
| LOC401321 | 1 |
| LOC401410 | 1 |
| LOC401898 | 1 |
| LOC402221 | 1 |
| LOC402382 | 1 |
| LOC402644 | 1 |
| LOC440030 | 1 |
| LOC440248 | 1 |
| LOC440345 | 1 |
| LOC440348 | 1 |
| LOC440525 | 1 |
| LOC440896 | 1 |
| LOC440926 | 1 |
| LOC441108 | 1 |
| LOC441155 | 1 |
| LOC441408 | 1 |
| LOC441891 | 1 |
| LOC442157 | 1 |
| LOC442181 | 1 |
| LOC51035 | 1 |
| LOC541471 | 1 |
| LOC613037 | 1 |
| LOC641806 | 1 |
| LOC642129 | 1 |
| LOC642209 | 1 |
| LOC642316 | 1 |
| LOC642321 | 1 |
| LOC642334 | 1 |
| LOC642516 | 1 |
| LOC642678 | 1 |
| LOC643043 | 1 |
| LOC643206 | 1 |
| LOC643276 | 1 |
| LOC643959 | 1 |
| LOC643997 | 1 |
| LOC644150 | 1 |
| LOC644241 | 1 |
| LOC644242 | 1 |
| LOC644246 | 1 |
| LOC644297 | 1 |
| LOC644310 | 1 |
| LOC644330 | 1 |
| LOC644388 | 1 |
| LOC644391 | 1 |
| LOC644482 | 1 |
| LOC644734 | 1 |
| LOC644869 | 1 |
| LOC645317 | 1 |
| LOC645492 | 1 |
| LOC645620 | 1 |
| LOC645730 | 1 |
| LOC646033 | 1 |
| LOC646236 | 1 |
| LOC646309 | 1 |
| LOC646357 | 1 |
| LOC646374 | 1 |
| LOC646533 | 1 |
| LOC646917 | 1 |
| LOC647081 | 1 |
| LOC647347 | 1 |
| LOC647407 | 1 |
| LOC647521 | 1 |
| LOC647649 | 1 |
| LOC647969 | 1 |
| LOC648405 | 1 |
| LOC648434 | 1 |
| LOC648604 | 1 |
| LOC648638 | 1 |
| LOC648733 | 1 |
| LOC648830 | 1 |
| LOC648907 | 1 |
| LOC648966 | 1 |
| LOC649320 | 1 |
| LOC649841 | 1 |
| LOC650263 | 1 |
| LOC650369 | 1 |
| LOC650965 | 1 |
| LOC651008 | 1 |
| LOC651309 | 1 |
| LOC651353 | 1 |
| LOC651436 | 1 |
| LOC651575 | 1 |
| LOC651751 | 1 |
| LOC651760 | 1 |
| LOC652468 | 1 |
| LOC652489 | 1 |
| LOC652490 | 1 |
| LOC652541 | 1 |
| LOC652614 | 1 |
| LOC652726 | 1 |
| LOC652771 | 1 |
| LOC652798 | 1 |
| LOC653103 | 1 |
| LOC653381 | 1 |
| LOC653496 | 1 |
| LOC653505 | 1 |
| LOC653600 | 1 |
| LOC653610 | 1 |
| LOC653874 | 1 |
| LOC654069 | 1 |
| LOC654074 | 1 |
| LOC654433 | 1 |
| LOC727761 | 1 |
| LOC727762 | 1 |
| LOC727848 | 1 |
| LOC727858 | 1 |
| LOC728006 | 1 |
| LOC728014 | 1 |
| LOC728222 | 1 |
| LOC728358 | 1 |
| LOC728505 | 1 |
| LOC728554 | 1 |
| LOC728764 | 1 |
| LOC728791 | 1 |
| LOC729148 | 1 |
| LOC729513 | 1 |
| LOC729623 | 1 |
| LOC729776 | 1 |
| LOC729937 | 1 |
| LOC729985 | 1 |
| Loc72B315 | 1 |
| LOC730024 | 1 |
| LOC730051 | 1 |
| LOC730092 | 1 |
| LOC730316 | 1 |
| LOC730549 | 1 |
| LOC730740 | 1 |
| LOC730833 | 1 |
| LOC731083 | 1 |
| LOC731170 | 1 |
| LOC731250 | 1 |
| LOC731295 | 1 |
| LOC731986 | 1 |
| LOC731999 | 1 |
| LOC732165 | 1 |
| LOC732360 | 1 |
| LOC791120 | 1 |
| LOC9039 | 1 |
| LOC90834 | 1 |
| LOC91948 | 1 |
| LOC92017 | 1 |
| LOC92345 | 1 |
| LOCI 29522 | 1 |
| LOCI 57489 | 1 |
| LOH12CR2 | 1 |
| LONP1 | 1 |
| LONRF3 | 1 |
| LOX | 1 |
| LPAR2 | 1 |
| LPAR5 | 1 |
| LPCAT3 | 1 |
| LPGAT1 | 1 |
| LPL | 1 |
| LRCH2 | 1 |
| LRCH4 | 1 |
| LRFN2 | 1 |
| LRFN4 | 1 |
| LRG1 | 1 |
| LRP5L | 1 |
| LRRC14 | 1 |
| LRRC17 | 1 |
| LRRC19 | 1 |
| LRRC23 | 1 |
| LRRC29 | 1 |
| LRRC32 | 1 |
| LRRC33 | 1 |
| LRRC37A15P | 1 |
| LRRC37A6P | 1 |
| LRRC41 | 1 |
| LRRC48 | 1 |
| LRRC4B | 1 |
| LRRC4C | 1 |
| LRRC53 | 1 |
| LRRC55 | 1 |
| LRRC57 | 1 |
| LRRC59 | 1 |
| LRRC6 | 1 |
| LRRC61 | 1 |
| LRRC71 | 1 |
| LRRC8A | 1 |
| LRRIQ1 | 1 |
| LRRTM2 | 1 |
| LRRTM3 | 1 |
| LRRTM4 | 1 |
| LRSAM1 | 1 |
| LRTM1 | 1 |
| LSAMP-AS3 | 1 |
| LSM1 | 1 |
| LSM14A | 1 |
| LSM5 | 1 |
| LSMD1 | 1 |
| LSP1 | 1 |
| LST1 | 1 |
| LTA | 1 |
| LTB4R | 1 |
| LTBR | 1 |
| LTK | 1 |
| LUM | 1 |
| LUZP1 | 1 |
| LY6G5B | 1 |
| LY6G5C | 1 |
| LY75 | 1 |
| LY9 | 1 |
| LY96 | 1 |
| LYAR | 1 |
| LYG1 | 1 |
| LYG2 | 1 |
| LYL1 | 1 |
| LYNX1 | 1 |
| LYPD1 | 1 |
| LYPD3 | 1 |
| LYPLAL1 | 1 |
| LYRM4 | 1 |
| LYRM5 | 1 |
| LYSMD2 | 1 |
| LYST | 1 |
| LZTFL1 | 1 |
| LZTR1 | 1 |
| M6PRBP1 | 1 |
| MAATS1 | 1 |
| MAB21L1 | 1 |
| MACF1 | 1 |
| MADD | 1 |
| MAF | 1 |
| MAFB | 1 |
| MAFG | 1 |
| MAG | 1 |
| MAGEC2 | 1 |
| MAGED1 | 1 |
| MAGEE1 | 1 |
| MAGEL2 | 1 |
| MAGI2 | 1 |
| MAGOH | 1 |
| MAGT1 | 1 |
| MALAT1 | 1 |
| MALL | 1 |
| MALT1 | 1 |
| MAML2 | 1 |
| MAN1B1 | 1 |
| MAN2B1 | 1 |
| MANBAL | 1 |
| MANF | 1 |
| MANI A2 | 1 |
| MAP1B | 1 |
| MAP1LC3B | 1 |
| MAP1S | 1 |
| MAP2K1IP1 | 1 |
| MAP3K10 | 1 |
| MAP3K11 | 1 |
| MAP3K2 | 1 |
| MAP3K4 | 1 |
| MAP3K6 | 1 |
| MAP4 | 1 |
| MAP4K2 | 1 |
| MAP7 | 1 |
| MAP7D1 | 1 |
| MAPI B | 1 |
| MAPK15 | 1 |
| MAPK3 | 1 |
| MAPK6 | 1 |
| MAPK8 | 1 |
| MAPK8IP1 | 1 |
| MAPKAPK2 | 1 |
| MAPRE2 | 1 |
| MAR3 | 1 |
| MAR4 | 1 |
| MARCH8 | 1 |
| MARCKS | 1 |
| MARCO | 1 |
| MARK3 | 1 |
| MAST3 | 1 |
| MAST4 | 1 |
| MATN2 | 1 |
| MAVS | 1 |
| MAZ | 1 |
| MB21D1 | 1 |
| MBD1 | 1 |
| MBD6 | 1 |
| MBLAC2 | 1 |
| MBNL3 | 1 |
| MBP | 1 |
| MC1R | 1 |
| MC3R | 1 |
| MC4R | 1 |
| MCAT | 1 |
| MCEE | 1 |
| MCFD2 | 1 |
| MCL1 | 1 |
| MCM3AP | 1 |
| MCM3AP-AS1 | 1 |
| MCM5 | 1 |
| MCRS1 | 1 |
| MCTP1 | 1 |
| MCTS1 | 1 |
| MDGA2 | 1 |
| MDK | 1 |
| MDN1 | 1 |
| MDP1 | 1 |
| ME1 | 1 |
| MED1 | 1 |
| MED13 | 1 |
| MED13L | 1 |
| MED14 | 1 |
| MED15 | 1 |
| MED25 | 1 |
| MED26 | 1 |
| MED8 | 1 |
| MEF2C-AS1 | 1 |
| MEGF10 | 1 |
| MEI1 | 1 |
| MEIS3 | 1 |
| MEMO1 | 1 |
| MET | 1 |
| METAP1D | 1 |
| METAP2 | 1 |
| METT10D | 1 |
| METTIOD | 1 |
| METTL1 | 1 |
| METTL13 | 1 |
| METTL21A | 1 |
| MFA p3 | 1 |
| MFAP1 | 1 |
| MFAP3L | 1 |
| MFGE8 | 1 |
| MFSD2A | 1 |
| MGAT1 | 1 |
| MGAT4B | 1 |
| MGAT5 | 1 |
| MGAT5B | 1 |
| MGC15763 | 1 |
| MGC16169 | 1 |
| MGC27348 | 1 |
| MGC3020 | 1 |
| MGC52282 | 1 |
| MGC87895 | 1 |
| MGLL | 1 |
| MGMT | 1 |
| MGP | 1 |
| MGRN1 | 1 |
| MGST1 | 1 |
| MGST3 | 1 |
| MIA | 1 |
| MICA | 1 |
| MICAL2 | 1 |
| MICALL2 | 1 |
| MIER1 | 1 |
| MIPOL1 | 1 |
| MIR100HG | 1 |
| MIR1181 | 1 |
| MIR1182 | 1 |
| MIR1184-1 | 1 |
| MIR1282 | 1 |
| MIR1304 | 1 |
| MIR1307 | 1 |
| MIR146A | 1 |
| MIR155HG | 1 |
| MIR222HG | 1 |
| MIR22HG | 1 |
| MIR4461 | 1 |
| MIR497HG | 1 |
| MIR548V | 1 |
| MIR564 | 1 |
| MIR600HG | 1 |
| MIR637 | 1 |
| MIR770 | 1 |
| MIR922 | 1 |
| MIR935 | 1 |
| MITD1 | 1 |
| MLF2 | 1 |
| MLH1 | 1 |
| MLKL | 1 |
| MLL | 1 |
| MLL4 | 1 |
| MLLT11 | 1 |
| MLLT3 | 1 |
| MLLT6 | 1 |
| MLXIP | 1 |
| MLXIPL | 1 |
| MMAA | 1 |
| MMD2 | 1 |
| MMEL1 | 1 |
| MMGT1 | 1 |
| MMP14 | 1 |
| MMP17 | 1 |
| MMP24-AS1 | 1 |
| MMP7 | 1 |
| MMRN2 | 1 |
| MMS22L | 1 |
| MN1 | 1 |
| MNT | 1 |
| MOAP1 | 1 |
| MOB1B | 1 |
| MOB3B | 1 |
| MOBKL2A | 1 |
| MOBP | 1 |
| MON1B | 1 |
| MON2 | 1 |
| MORF4 | 1 |
| MOSPD3 | 1 |
| MOXD1 | 1 |
| MPDU1 | 1 |
| MPG | 1 |
| MPHOSPH10 | 1 |
| MPI | 1 |
| MPND | 1 |
| MPO | 1 |
| MPP5 | 1 |
| MPPED1 | 1 |
| MPRIP-AS1 | 1 |
| MPST | 1 |
| MPZL2 | 1 |
| MRAP | 1 |
| MRAP2 | 1 |
| MRAS | 1 |
| MRFAP1 | 1 |
| MRGBP | 1 |
| MRI1 | 1 |
| MRLC2 | 1 |
| MRPL15 | 1 |
| MRPL18 | 1 |
| MRPL21 | 1 |
| MRPL23 | 1 |
| MRPL24 | 1 |
| MRPL33 | 1 |
| MRPL37P1 | 1 |
| MRPL39 | 1 |
| MRPL41 | 1 |
| MRPL47 | 1 |
| MRPL51 | 1 |
| MRPL52 | 1 |
| MRPL54 | 1 |
| MRPL55 | 1 |
| MRPS16 | 1 |
| MRPS18C | 1 |
| MRPS21 | 1 |
| MRPS22 | 1 |
| MS4A6A | 1 |
| MSC | 1 |
| MSH6 | 1 |
| MSI1 | 1 |
| MSMP | 1 |
| MSRA | 1 |
| MSRB2 | 1 |
| MSRB3 | 1 |
| MST1L | 1 |
| MST4 | 1 |
| MSTN | 1 |
| MSTO1 | 1 |
| MSTP9 | 1 |
| MT-ATP6 | 1 |
| MT-CO1 | 1 |
| MT-CO2 | 1 |
| MT-CYB | 1 |
| MT-ND1 | 1 |
| MT-ND2 | 1 |
| MT-ND3 | 1 |
| MT-ND5 | 1 |
| MT-ND6 | 1 |
| MT1A | 1 |
| MT1E | 1 |
| MT1G | 1 |
| MT1JP | 1 |
| MT1L | 1 |
| MT1M | 1 |
| MT3 | 1 |
| MTA2 | 1 |
| MTCH1 | 1 |
| MTCP1 | 1 |
| MTERFD1 | 1 |
| MTFMT | 1 |
| MTHFD2 | 1 |
| MTIF3 | 1 |
| MTMR2 | 1 |
| MTMR4 | 1 |
| MTMR6 | 1 |
| MTMR7 | 1 |
| MTMR9 | 1 |
| MTND5P16 | 1 |
| MTRFIL | 1 |
| MTRNR2L1 | 1 |
| MTRNR2L10 | 1 |
| MTRNR2L5 | 1 |
| MTRNR2L6 | 1 |
| MTRNR2L7 | 1 |
| MTRNR2L8 | 1 |
| MTSS1 | 1 |
| MTX3 | 1 |
| MUC1 | 1 |
| MUC20 | 1 |
| MUM1 | 1 |
| MX2 | 1 |
| MXD4 | 1 |
| MXRA8 | 1 |
| MYADM | 1 |
| MYADML2 | 1 |
| MYBBP1A | 1 |
| MYBPC2 | 1 |
| MYBPH | 1 |
| MYCBP | 1 |
| MYCT1 | 1 |
| MYD88 | 1 |
| MYEF2 | 1 |
| MYH14 | 1 |
| MYH4 | 1 |
| MYH9 | 1 |
| MYL12A | 1 |
| MYL12B | 1 |
| MYL4 | 1 |
| MYL5 | 1 |
| MYL9 | 1 |
| MYO16 | 1 |
| MYO1C | 1 |
| MYO1F | 1 |
| MYo9A | 1 |
| MYO9B | 1 |
| MYSMI | 1 |
| MYST3 | 1 |
| MZB1 | 1 |
| MZT2B | 1 |
| N4BP2L1 | 1 |
| N6AMT1 | 1 |
| NAA15 | 1 |
| NAA16 | 1 |
| NAA20 | 1 |
| NAAA | 1 |
| NABP1 | 1 |
| NAG18 | 1 |
| NALCN | 1 |
| NAMPT | 1 |
| NANOS3 | 1 |
| NANP | 1 |
| NANS | 1 |
| NAP1L1 | 1 |
| NAP1L3 | 1 |
| NAP1L4 | 1 |
| NAP1L5 | 1 |
| NAPA | 1 |
| NAPIL3 | 1 |
| NARG2 | 1 |
| NASP | 1 |
| NAT10 | 1 |
| NAT5 | 1 |
| NAT8L | 1 |
| NAV2-AS4 | 1 |
| NBEAL1 | 1 |
| NBN | 1 |
| NBPF8 | 1 |
| NCALD | 1 |
| NCAPH2 | 1 |
| NCDN | 1 |
| NCF1 | 1 |
| NCF1C | 1 |
| NCF4 | 1 |
| NCK1 | 1 |
| NCK2 | 1 |
| NCKIPSD | 1 |
| NCL | 1 |
| NCLN | 1 |
| NCOA1 | 1 |
| NCOR2 | 1 |
| NCRNA00169 | 1 |
| NCS1 | 1 |
| NDNF | 1 |
| NDOR1 | 1 |
| NDRG4 | 1 |
| NDST2 | 1 |
| NDUFA11 | 1 |
| NDUFA2 | 1 |
| NDUFA5 | 1 |
| NDUFA6-AS1 | 1 |
| NDUFAB1 | 1 |
| NDUFB11 | 1 |
| NDUFB3 | 1 |
| NDUFB4 | 1 |
| NDUFB9 | 1 |
| NDUFC1 | 1 |
| NDUFS3 | 1 |
| NDUFS7 | 1 |
| NDUFV2 | 1 |
| NEAT1 | 1 |
| NEDD9 | 1 |
| NEFH | 1 |
| NEFL | 1 |
| NEGR1 | 1 |
| NEIL2 | 1 |
| NEK6 | 1 |
| NELF | 1 |
| NELL1 | 1 |
| NELL2 | 1 |
| NEMP1 | 1 |
| NEMP2 | 1 |
| NENF | 1 |
| NES | 1 |
| NETI | 1 |
| NETO1 | 1 |
| NETO2 | 1 |
| NEU4 | 1 |
| NEURL3 | 1 |
| NEUROD1 | 1 |
| NEUROD6 | 1 |
| NEUROG2 | 1 |
| NF1 | 1 |
| NFASC | 1 |
| NFAT5 | 1 |
| NFATC3 | 1 |
| NFATC4 | 1 |
| NFE2L3 | 1 |
| NFIL3 | 1 |
| NFKB1 | 1 |
| NFKBID | 1 |
| NFKBIE | 1 |
| NFXL1 | 1 |
| NFYA | 1 |
| NGFR | 1 |
| NHLH1 | 1 |
| NHP2 | 1 |
| NICN1 | 1 |
| NIF3L1 | 1 |
| NINJ1 | 1 |
| NISCH | 1 |
| NKG7 | 1 |
| NKX2-3 | 1 |
| NLE1 | 1 |
| NLGN1 | 1 |
| NLN | 1 |
| NLRC5 | 1 |
| NLRP1 | 1 |
| NLRX1 | 1 |
| NMB | 1 |
| NME4 | 1 |
| NME7 | 1 |
| NMI | 1 |
| NMNAT2 | 1 |
| NMT1 | 1 |
| NNMT | 1 |
| NOB1 | 1 |
| NOBOX | 1 |
| NOC3L | 1 |
| NOC4L | 1 |
| NOD1 | 1 |
| NOL1 | 1 |
| NOL10 | 1 |
| NOL5A | 1 |
| NOL6 | 1 |
| NOL7 | 1 |
| NOL8 | 1 |
| NOMO1 | 1 |
| NOMO2 | 1 |
| NOP2 | 1 |
| NOP58 | 1 |
| NOS1 | 1 |
| NOSTRIN | 1 |
| NOTCH4 | 1 |
| NOVA1 | 1 |
| NOVA2 | 1 |
| NPAS1 | 1 |
| NPAS4 | 1 |
| NPB | 1 |
| NPBWR2 | 1 |
| NPEPL1 | 1 |
| NPFF | 1 |
| NPHS1 | 1 |
| NPL | 1 |
| NPM2 | 1 |
| NPPB | 1 |
| NPR1 | 1 |
| NPSR1-AS1 | 1 |
| NPTX1 | 1 |
| NPTXR | 1 |
| NPY | 1 |
| NPY6R | 1 |
| NQO1 | 1 |
| NR1D1 | 1 |
| NR1H3 | 1 |
| NR2F1 | 1 |
| NR2F2 | 1 |
| NR4A2 | 1 |
| NRF1 | 1 |
| NRIH4 | 1 |
| NRIP3 | 1 |
| NRM | 1 |
| NRSN1 | 1 |
| NRSN2 | 1 |
| NRXN1 | 1 |
| NSA2 | 1 |
| NSD1 | 1 |
| NSL1 | 1 |
| NSMCE4A | 1 |
| NT5C | 1 |
| NT5DC2 | 1 |
| NT5M | 1 |
| NTHL1 | 1 |
| NTNG2 | 1 |
| NTRK1 | 1 |
| NTRK2 | 1 |
| NTSR1 | 1 |
| NTSR2 | 1 |
| NUAK1 | 1 |
| NUBPL | 1 |
| NUCKS1 | 1 |
| NUDCD1 | 1 |
| NUDCD3 | 1 |
| NUDT10 | 1 |
| NUDT11 | 1 |
| NUDT15 | 1 |
| NUDT17 | 1 |
| NUDT18 | 1 |
| NUDT4P1 | 1 |
| NUDT6 | 1 |
| NUFIP1 | 1 |
| NUGGC | 1 |
| NUMA1 | 1 |
| NUMB | 1 |
| NUP153 | 1 |
| NUP155 | 1 |
| NUP50 | 1 |
| NUP93 | 1 |
| NUPR1 | 1 |
| NUS1 | 1 |
| NVL | 1 |
| NXN | 1 |
| NXPE3 | 1 |
| NXPH1 | 1 |
| NXPH2 | 1 |
| NXPH3 | 1 |
| NXPH4 | 1 |
| NYAP2 | 1 |
| O3FAR1 | 1 |
| OAF | 1 |
| OAZ1 | 1 |
| OBFC2A | 1 |
| ODF3B | 1 |
| ODZ1 | 1 |
| ODZ2 | 1 |
| ODZ3 | 1 |
| OFD1 | 1 |
| OGFR | 1 |
| OGT | 1 |
| OLA1 | 1 |
| OLFM1 | 1 |
| OLFML3 | 1 |
| OLIG1 | 1 |
| OMA1 | 1 |
| OMD | 1 |
| OPHN1 | 1 |
| OPLAH | 1 |
| OPRM1 | 1 |
| OR14C36 | 1 |
| OR2A42 | 1 |
| OR2D2 | 1 |
| OR2L13 | 1 |
| OR2M4 | 1 |
| OR2W3 | 1 |
| OR4N4 | 1 |
| OR51E1 | 1 |
| OR5AR1 | 1 |
| ORAI2 | 1 |
| ORC4 | 1 |
| ORC6L | 1 |
| ORM1 | 1 |
| ORM2 | 1 |
| OSBP | 1 |
| OSBPL7 | 1 |
| OSBPL8 | 1 |
| OSGEP | 1 |
| OSGIN1 | 1 |
| OSGIN2 | 1 |
| OSMR | 1 |
| OSTC | 1 |
| OSTCP1 | 1 |
| OSTCP8 | 1 |
| OTUD5 | 1 |
| OVGP1 | 1 |
| OXCT1 | 1 |
| OXGR1 | 1 |
| OXR1 | 1 |
| OXSR1 | 1 |
| OXT | 1 |
| P117 | 1 |
| P2RX5 | 1 |
| P2RY11 | 1 |
| P2RY13 | 1 |
| P2RY8 | 1 |
| P4HB | 1 |
| P4HTM | 1 |
| P704P | 1 |
| PABPC1 | 1 |
| PABPC4 | 1 |
| PACS1 | 1 |
| PACSIN2 | 1 |
| PACSIN3 | 1 |
| PACSlN2 | 1 |
| PADI2 | 1 |
| PAF1 | 1 |
| PAFAH1B1 | 1 |
| PAG1 | 1 |
| PAIP2 | 1 |
| PAIP2B | 1 |
| PAK2 | 1 |
| PALD1 | 1 |
| PALM | 1 |
| PALM2 | 1 |
| PAM | 1 |
| pAMCl | 1 |
| PAMR1 | 1 |
| PAN2 | 1 |
| PAN3 | 1 |
| PAPD1 | 1 |
| PAPD4 | 1 |
| PAPLN | 1 |
| PAPOLA | 1 |
| PAPOLB | 1 |
| PAPPA2 | 1 |
| PAPSS2 | 1 |
| PAQR5 | 1 |
| PAQR6 | 1 |
| PARD6A | 1 |
| PARK7 | 1 |
| PARL | 1 |
| PARM1 | 1 |
| PARP3 | 1 |
| PARP4 | 1 |
| PARP8 | 1 |
| PATE2 | 1 |
| PATL1 | 1 |
| PBOV1 | 1 |
| PBRM1 | 1 |
| PBX1 | 1 |
| PBX2 | 1 |
| PBX3 | 1 |
| PCA3 | 1 |
| PCBD1 | 1 |
| PCBP1 | 1 |
| PCDH11X | 1 |
| PCDH11Y | 1 |
| PCDH17 | 1 |
| PCDH20 | 1 |
| PCDH8 | 1 |
| PCDHA10 | 1 |
| PCDHA3 | 1 |
| PCDHA4 | 1 |
| PCDHA8 | 1 |
| PCDHB10 | 1 |
| PCDHB9 | 1 |
| PCDHGA2 | 1 |
| PCDHGC3 | 1 |
| PCGF5 | 1 |
| PCIF1 | 1 |
| PCK2 | 1 |
| PCMT1 | 1 |
| PCNP | 1 |
| PCNT | 1 |
| PCNX | 1 |
| PCNX1 | 1 |
| PCOLCE2 | 1 |
| PCP4 | 1 |
| PCSK1 | 1 |
| PCSK1N | 1 |
| PCSK7 | 1 |
| PDCD10 | 1 |
| PDCD1LG2 | 1 |
| PDCD4 | 1 |
| PDCD6 | 1 |
| PDDC1 | 1 |
| PDE12 | 1 |
| PDE1A | 1 |
| PDE2A | 1 |
| PDE3A | 1 |
| PDE3B | 1 |
| PDE4B | 1 |
| PDE4D | 1 |
| PDE6A | 1 |
| PDE6B | 1 |
| PDE7A | 1 |
| PDGFB | 1 |
| PDIA4 | 1 |
| PDIA5 | 1 |
| PDIA6 | 1 |
| PDK1 | 1 |
| PDK2 | 1 |
| PDK3 | 1 |
| PDLIM1 | 1 |
| PDLIM4 | 1 |
| PDP2 | 1 |
| PDPR | 1 |
| PDS5A | 1 |
| PDS5B | 1 |
| PDSS1 | 1 |
| PDYN | 1 |
| PDZD4 | 1 |
| PDZK1IP1 | 1 |
| PDZRN4 | 1 |
| PEAR1 | 1 |
| PEBP1 | 1 |
| PEF1 | 1 |
| PEG10 | 1 |
| PEG3 | 1 |
| PELI3 | 1 |
| PELO | 1 |
| PEMT | 1 |
| PEPD | 1 |
| PER1 | 1 |
| PERP | 1 |
| PES1 | 1 |
| PEX10 | 1 |
| PEX11B | 1 |
| PEX14 | 1 |
| PEX16 | 1 |
| PEX2 | 1 |
| PFAAP5 | 1 |
| PFDN1 | 1 |
| PFDN5 | 1 |
| PFDN6 | 1 |
| PFKFB2 | 1 |
| PFKL | 1 |
| PFKM | 1 |
| PFN1 | 1 |
| PFN1P2 | 1 |
| PFN2 | 1 |
| PGAM1 | 1 |
| PGAP1 | 1 |
| PGC | 1 |
| PGD | 1 |
| PGLYRP1 | 1 |
| PGM2L1 | 1 |
| PGM5 | 1 |
| PGPEP1 | 1 |
| PH KG2 | 1 |
| PHB | 1 |
| PHB2 | 1 |
| PHF14 | 1 |
| PHF15 | 1 |
| pHF19 | 1 |
| PHF19 | 1 |
| PHF2 | 1 |
| PHF20 | 1 |
| PHF20L1 | 1 |
| PHF3 | 1 |
| PHF5A | 1 |
| PHIP | 1 |
| PHKA1 | 1 |
| PHKA2 | 1 |
| PHOSPHO1 | 1 |
| PHOX2A | 1 |
| PHRF1 | 1 |
| PHYH | 1 |
| PHYHD1 | 1 |
| PI15 | 1 |
| PI4K2A | 1 |
| PI4KA | 1 |
| PI4KAP2 | 1 |
| PID1 | 1 |
| PIDD | 1 |
| PIEZO1 | 1 |
| PIGC | 1 |
| PIGF | 1 |
| PIGH | 1 |
| PIGM | 1 |
| PIGN | 1 |
| PIGQ | 1 |
| PIGW | 1 |
| PIGY | 1 |
| PIK3C2A | 1 |
| PIK3CB | 1 |
| PIK4CA | 1 |
| PIKFYVE | 1 |
| PILRA | 1 |
| PIM1 | 1 |
| PIM3 | 1 |
| PINK1-AS | 1 |
| PION | 1 |
| PIP4K2C | 1 |
| PIP5K1C | 1 |
| PIP5K1P1 | 1 |
| PIP5K2A | 1 |
| PIP5K2B | 1 |
| PIP5KL1 | 1 |
| PITPNA | 1 |
| PITPNM2 | 1 |
| PITPNM3 | 1 |
| PIWIL2 | 1 |
| PIWIL4 | 1 |
| PKD2 | 1 |
| PKD2L2 | 1 |
| PKDCC | 1 |
| PKIB | 1 |
| PKIG | 1 |
| PKM2 | 1 |
| PKMYT1 | 1 |
| PKN3 | 1 |
| PKP2 | 1 |
| PLA2G2D | 1 |
| PLA2G4B | 1 |
| PLA2G5 | 1 |
| PLAC8 | 1 |
| PLAC9 | 1 |
| PLAGL1 | 1 |
| PLAGL2 | 1 |
| PLBD1-AS1 | 1 |
| PLCB3 | 1 |
| PLCB4 | 1 |
| PLCD4 | 1 |
| PLCE1 | 1 |
| PLCL2 | 1 |
| PLCXD1 | 1 |
| PLCXD3 | 1 |
| PLD4 | 1 |
| PLDN | 1 |
| PLEK | 1 |
| PLEKHA1 | 1 |
| PLEKHA2 | 1 |
| PLEKHA4 | 1 |
| PLEKHG3 | 1 |
| PLEKHG5 | 1 |
| PLEKHJ1 | 1 |
| PLEKHM2 | 1 |
| PLEKHO1 | 1 |
| PLGLB1 | 1 |
| PLGLB2 | 1 |
| PLIN2 | 1 |
| PLIN3 | 1 |
| PLK5 | 1 |
| PLLP | 1 |
| PLOD1 | 1 |
| PLOD3 | 1 |
| PLP1 | 1 |
| PLP2 | 1 |
| PLPP5 | 1 |
| PLPPR5 | 1 |
| PLXDC1 | 1 |
| PMAIP1 | 1 |
| PMEPA1 | 1 |
| PMF1 | 1 |
| PML | 1 |
| PMM2 | 1 |
| PMPCA | 1 |
| PMS2L4 | 1 |
| PMS2L5 | 1 |
| PMVK | 1 |
| PNISR | 1 |
| PNKD | 1 |
| PNKP | 1 |
| PNMA1 | 1 |
| PNMA5 | 1 |
| PNMAL1 | 1 |
| PNP | 1 |
| PNPLA2 | 1 |
| PNPLA6 | 1 |
| PNPT1 | 1 |
| PNRC1 | 1 |
| PNRC2 | 1 |
| POFUT1 | 1 |
| POLD4 | 1 |
| POLDIP2 | 1 |
| POLDIP3 | 1 |
| POLE3 | 1 |
| POLG | 1 |
| POLH | 1 |
| POLK | 1 |
| POLR1C | 1 |
| POLR1D | 1 |
| POLR2A | 1 |
| POLR2C | 1 |
| POLR2F | 1 |
| POLR2I | 1 |
| POLR2J2 | 1 |
| POLR2J3 | 1 |
| POLR2L | 1 |
| POLR3G | 1 |
| POLR3GL | 1 |
| POLR3H | 1 |
| POM121 | 1 |
| POM121C | 1 |
| POMP | 1 |
| POMT1 | 1 |
| POP1 | 1 |
| POTEJ | 1 |
| POU2AF1 | 1 |
| POU2F1 | 1 |
| POU2F2 | 1 |
| POU4F1 | 1 |
| PPA2 | 1 |
| PPAN | 1 |
| PPAPDC1B | 1 |
| PPBP | 1 |
| PPCDC | 1 |
| PPFIA1 | 1 |
| PPFIA4 | 1 |
| PPFIBP1 | 1 |
| PPFIBP2 | 1 |
| PPIA | 1 |
| PPIB | 1 |
| PPIC | 1 |
| PPID | 1 |
| PPIH | 1 |
| PPIL3 | 1 |
| PPM1A | 1 |
| PPM1F | 1 |
| PPM1N | 1 |
| PPME1 | 1 |
| PPOX | 1 |
| PPP1CC | 1 |
| PPP1R11 | 1 |
| PPP1R12B | 1 |
| PPP1R12C | 1 |
| PPP1R14A | 1 |
| PPP1R14B | 1 |
| PPP1R14C | 1 |
| PPP1R16A | 1 |
| PPP1R16B | 1 |
| PPP1R18 | 1 |
| PPP1R1A | 1 |
| PPP1R3E | 1 |
| PPP1R8P1 | 1 |
| PPP2R2A | 1 |
| PPP2R2B | 1 |
| PPP2R2D | 1 |
| PPP2R3A | 1 |
| PPP2R4 | 1 |
| PPP2R5C | 1 |
| PPP3CA | 1 |
| PPP3CC | 1 |
| PPP3R2 | 1 |
| PPP6R2 | 1 |
| PPRC1 | 1 |
| PPT1 | 1 |
| PPTC7 | 1 |
| PQLC1 | 1 |
| PQLC3 | 1 |
| PRADC1 | 1 |
| PRAF2 | 1 |
| PRAM1 | 1 |
| PRAMEF11 | 1 |
| PRB2 | 1 |
| PRDM1 | 1 |
| PRDX3 | 1 |
| PRDX4 | 1 |
| PRELID1 | 1 |
| PREX1 | 1 |
| PRF1 | 1 |
| PRH1 | 1 |
| PRICKLE1 | 1 |
| PRICKLE2 | 1 |
| PRICKLE4 | 1 |
| PRKAA1 | 1 |
| PRKACA | 1 |
| PRKAG2 | 1 |
| PRKAR1B | 1 |
| PRKCA | 1 |
| PRKCDBP | 1 |
| PRKCG | 1 |
| PRKCH | 1 |
| PRKD2 | 1 |
| PRKG1 | 1 |
| PRKRIR | 1 |
| PRKX | 1 |
| PRKY | 1 |
| PRLR | 1 |
| PRMT1 | 1 |
| PRMT2 | 1 |
| PRMT6 | 1 |
| PRNP | 1 |
| PRO0478 | 1 |
| PROCR | 1 |
| PROSER1 | 1 |
| PROZ | 1 |
| PRPH2 | 1 |
| PRPSAP2 | 1 |
| PRR14 | 1 |
| PRR14L | 1 |
| PRR15 | 1 |
| PRR24 | 1 |
| PRRC1 | 1 |
| PRRG3 | 1 |
| PRRG4 | 1 |
| PRSS1 | 1 |
| PRUNE2 | 1 |
| PRX | 1 |
| psAp | 1 |
| PSCD1 | 1 |
| PSD | 1 |
| PSD4 | 1 |
| PSMA1 | 1 |
| PSMA2 | 1 |
| PSMA3 | 1 |
| PSMA6 | 1 |
| PSMB2 | 1 |
| PSMB3 | 1 |
| PSMB5 | 1 |
| PSMB7 | 1 |
| PSMC1 | 1 |
| PSMC2 | 1 |
| PSMC3 | 1 |
| PSMC3IP | 1 |
| PSMC4 | 1 |
| PSMD1 | 1 |
| PSMD10 | 1 |
| PSMD2 | 1 |
| PSMD4 | 1 |
| PSMD6 | 1 |
| PSMD8 | 1 |
| PSME1 | 1 |
| PSME2 | 1 |
| PSPH | 1 |
| PTBP1 | 1 |
| PTBP2 | 1 |
| PTEN | 1 |
| PTGDS | 1 |
| PTGER2 | 1 |
| PTGES3 | 1 |
| PTGFR | 1 |
| PTGIR | 1 |
| PTH | 1 |
| PTK7 | 1 |
| PTMAP5 | 1 |
| PTOV1 | 1 |
| PTP4A1 | 1 |
| PTPA | 1 |
| PTPLA | 1 |
| PTPLB | 1 |
| PTPN1 | 1 |
| PTPN12 | 1 |
| PTPN2 | 1 |
| PTPN7 | 1 |
| PTPRCAP | 1 |
| PTPRE | 1 |
| PTPRK | 1 |
| PTPRO | 1 |
| PTPRR | 1 |
| PTPRT | 1 |
| PTPRU | 1 |
| PTRH1 | 1 |
| PTRHD1 | 1 |
| PTTG3 | 1 |
| PTX3 | 1 |
| PUM2 | 1 |
| PUS7L | 1 |
| PVRIG | 1 |
| PVRL1 | 1 |
| PVRL2 | 1 |
| PXK | 1 |
| PYCARD | 1 |
| PYCR2 | 1 |
| PYCRL | 1 |
| PYGB | 1 |
| PYGL | 1 |
| PYGM | 1 |
| PYROXD2 | 1 |
| QARS | 1 |
| QPCT | 1 |
| QPRT | 1 |
| QRSL1P3 | 1 |
| QSOX2 | 1 |
| R3HCC1 | 1 |
| RAB10 | 1 |
| RAB11A | 1 |
| RAB11FIP2 | 1 |
| RAB12 | 1 |
| RAB15 | 1 |
| RAB18 | 1 |
| RAB1B | 1 |
| RAB20 | 1 |
| RAB21 | 1 |
| RAB22A | 1 |
| RAB2A | 1 |
| RAB2B | 1 |
| RAB32 | 1 |
| RAB33A | 1 |
| RAB34 | 1 |
| RAB35 | 1 |
| RAB38 | 1 |
| RAB39A | 1 |
| RAB3A | 1 |
| RAB3B | 1 |
| RAB3GAP1 | 1 |
| RAB3GAP2 | 1 |
| RAB5A | 1 |
| RAB5C | 1 |
| RAB8A | 1 |
| RAB9A | 1 |
| RAB9B | 1 |
| RABAC1 | 1 |
| RABGAP1L | 1 |
| RABL2A | 1 |
| RABL2B | 1 |
| RABL5 | 1 |
| RABL6 | 1 |
| RAD1 | 1 |
| RAD51D | 1 |
| RALGAPB | 1 |
| RALGPS1 | 1 |
| RALGPS2 | 1 |
| RALY | 1 |
| RAMP1 | 1 |
| RAMP2 | 1 |
| RAMP3 | 1 |
| RANBP1 | 1 |
| RANBP3 | 1 |
| RANBP5 | 1 |
| RANGAP1 | 1 |
| RAP1BL | 1 |
| RAP1GAP2 | 1 |
| RAP2A | 1 |
| RAP2C | 1 |
| RAPGEF6 | 1 |
| RARRES2 | 1 |
| RARS | 1 |
| RASA3 | 1 |
| RASAL3 | 1 |
| RASD1 | 1 |
| RASD2 | 1 |
| RASGEF1A | 1 |
| RASGEF1B | 1 |
| RASL11B | 1 |
| RASSF I | 1 |
| RASSF4 | 1 |
| RAX2 | 1 |
| RAXL1 | 1 |
| RBBP9 | 1 |
| RBFOX1 | 1 |
| RBFOX2 | 1 |
| RBFOX3 | 1 |
| RBI | 1 |
| RBM | 1 |
| RBM10 | 1 |
| RBM25 | 1 |
| RBM33 | 1 |
| RBM38 | 1 |
| RBM47 | 1 |
| RBM48 | 1 |
| RBM5 | 1 |
| RBM8A | 1 |
| RBMS1 | 1 |
| RBMS2 | 1 |
| RBMY1A1 | 1 |
| RBMY1B | 1 |
| RBMY1D | 1 |
| RBMY1E | 1 |
| RBMY1F | 1 |
| RBMY1H | 1 |
| RBMY1J | 1 |
| RBMY3AP | 1 |
| RBP1 | 1 |
| RC3H1 | 1 |
| RC3H2 | 1 |
| RCAN2 | 1 |
| RCBTB2 | 1 |
| RCSD1 | 1 |
| RCVRN | 1 |
| REC8 | 1 |
| RECQL4 | 1 |
| RELA | 1 |
| RELL1 | 1 |
| RELL2 | 1 |
| RELN | 1 |
| REM2 | 1 |
| REST | 1 |
| RETSAT | 1 |
| REXO2 | 1 |
| RFFL | 1 |
| RFNG | 1 |
| RFPL1-AS1 | 1 |
| RFPL2 | 1 |
| RFTN1 | 1 |
| RFX2 | 1 |
| RFX3 | 1 |
| RFX7 | 1 |
| RFXAP | 1 |
| RFXDC2 | 1 |
| RGL4 | 1 |
| RGPD5 | 1 |
| RGS11 | 1 |
| RGS12 | 1 |
| RGS14 | 1 |
| RGS16 | 1 |
| RGS18 | 1 |
| RGS4 | 1 |
| RGS9 | 1 |
| RHBDD1 | 1 |
| RHBDD3 | 1 |
| RHBDF1 | 1 |
| RHBDF2 | 1 |
| RHBDL1 | 1 |
| RHEB | 1 |
| RHEBL1 | 1 |
| RHO | 1 |
| RHOF | 1 |
| RHOG | 1 |
| RHOT1 | 1 |
| RHOV | 1 |
| RHPN1 | 1 |
| RICTOR | 1 |
| RIFI | 1 |
| RILP | 1 |
| RILPL2 | 1 |
| RIN1 | 1 |
| RIN2 | 1 |
| RINT1 | 1 |
| RIOK2 | 1 |
| RIOK3 | 1 |
| RIPK2 | 1 |
| RIPK3 | 1 |
| RLBP1 | 1 |
| RMND5B | 1 |
| RMRP | 1 |
| RN5S9 | 1 |
| RN7SK | 1 |
| RNASE4 | 1 |
| RNASEH2C | 1 |
| RNASEK-C17ORF49 | 1 |
| RNF103 | 1 |
| RNF113B | 1 |
| RNF121 | 1 |
| RNF122 | 1 |
| RNF126 | 1 |
| RNF13 | 1 |
| RNF130 | 1 |
| RNF133 | 1 |
| RNF144B | 1 |
| RNF150 | 1 |
| RNF165 | 1 |
| RNF166 | 1 |
| RNF181 | 1 |
| RNF208 | 1 |
| RNF213 | 1 |
| RNF215 | 1 |
| RNF216 | 1 |
| RNF219 | 1 |
| RNF25 | 1 |
| RNF31 | 1 |
| RNF4 | 1 |
| RNF40 | 1 |
| RNF7 | 1 |
| RNMT | 1 |
| RNPEPL1 | 1 |
| RNU105A | 1 |
| RNU6ATAC16P | 1 |
| RNU7-1 | 1 |
| ROBO1 | 1 |
| ROBO3 | 1 |
| ROBO4 | 1 |
| ROCK 1 | 1 |
| ROM1 | 1 |
| ROMO1 | 1 |
| ROPN1B | 1 |
| RP1-40E16.11 | 1 |
| RP1-85F18.6 | 1 |
| RP1-92O14.6 | 1 |
| RP11-1079K10.4 | 1 |
| RP11-108P20.3 | 1 |
| RP11-126O1.4 | 1 |
| RP11-143K11.1 | 1 |
| RP11-164N3.3 | 1 |
| RP11-166B2.1 | 1 |
| RP11-166P13.3 | 1 |
| RP11-16E12.1 | 1 |
| RP11-16E12.2 | 1 |
| RP11-19P22.8 | 1 |
| RP11-215G15.5 | 1 |
| RP11-26L20.3 | 1 |
| RP11-277P12.20 | 1 |
| RP11-279O9.4 | 1 |
| RP11-284F21.10 | 1 |
| RP11-284F21.7 | 1 |
| RP11-284F21.9 | 1 |
| RP11-297C4.1 | 1 |
| RP11-297C4.2 | 1 |
| RP11-399O19.9 | 1 |
| RP11-417F21.1 | 1 |
| RP11-421F16.3 | 1 |
| RP11-463J10.3 | 1 |
| RP11-465B22.3 | 1 |
| RP11-466M21.1 | 1 |
| RP11-467L13.5 | 1 |
| RP11-476H24.1 | 1 |
| RP11-490O6.2 | 1 |
| RP11-498C9.2 | 1 |
| RP11-523O18.5 | 1 |
| RP11-523O18.7 | 1 |
| RP11-549B18.1 | 1 |
| RP11-54O7.18 | 1 |
| RP11-568N6.1 | 1 |
| RP11-58K22.5 | 1 |
| RP11-632F7.3 | 1 |
| RP11-670E13.5 | 1 |
| RP11-681H18.2 | 1 |
| RP11-69E11.8 | 1 |
| RP11-69L16.4 | 1 |
| RP11-73E17.2 | 1 |
| RP11-78B10.2 | 1 |
| RP11-79H23.3 | 1 |
| RP11-804A23.4 | 1 |
| RP11-809N8.2 | 1 |
| RP11-867G2.8 | 1 |
| RP11-89H19.1 | 1 |
| RP11-96C23.15 | 1 |
| RP3-416H24.1 | 1 |
| RP3-428L16.2 | 1 |
| RP4-622L5.7 | 1 |
| RP5-1057I20.4 | 1 |
| RP5-1071N3.1 | 1 |
| RP5-1180E21.5 | 1 |
| RP5-837M10.4 | 1 |
| RP5-894A10.2 | 1 |
| RPA3 | 1 |
| RPAP2 | 1 |
| RPE | 1 |
| RPF2 | 1 |
| RPGRIP1L | 1 |
| RPH3A | 1 |
| RPIA | 1 |
| RPL12 | 1 |
| RPL13 | 1 |
| RPL13A | 1 |
| RPL13P6 | 1 |
| RPL14 | 1 |
| RPL17 | 1 |
| RPL21 | 1 |
| RPL22L1 | 1 |
| RPL23 | 1 |
| RPL23AP79 | 1 |
| RPL24 | 1 |
| RPL27A | 1 |
| RPL30 | 1 |
| RPL31 | 1 |
| RPL34 | 1 |
| RPL35A | 1 |
| RPL35P6 | 1 |
| RPL36 | 1 |
| RPL37A | 1 |
| RPL37AP8 | 1 |
| RPL39 | 1 |
| RPL39P5 | 1 |
| RPL4 | 1 |
| RPL5 | 1 |
| RPL6P7 | 1 |
| RPL7 | 1 |
| RPL7L1 | 1 |
| RPL7L1P2 | 1 |
| RPL8 | 1 |
| RPL9 | 1 |
| RPN1 | 1 |
| RPN2 | 1 |
| RPP25L | 1 |
| RPPH1 | 1 |
| RPRC1 | 1 |
| RPRD1A | 1 |
| RPRML | 1 |
| RPS10 | 1 |
| RPS10-NUDT3 | 1 |
| RPS10P7 | 1 |
| RPS11 | 1 |
| RPS13 | 1 |
| RPS15A | 1 |
| RPS16 | 1 |
| RPS18 | 1 |
| RPS19 | 1 |
| RPS24 | 1 |
| RPS26 | 1 |
| RPS26L | 1 |
| RPS26P11 | 1 |
| RPS27 | 1 |
| RPS28 | 1 |
| RPS29 | 1 |
| RPS3A | 1 |
| RPS4XP16 | 1 |
| RPS6KA1 | 1 |
| RPS6KA4 | 1 |
| RPS6KB1 | 1 |
| RPS6KC1 | 1 |
| RPS6KL1 | 1 |
| RPS6P1 | 1 |
| RPS8 | 1 |
| RPSAP58 | 1 |
| RPUSD1 | 1 |
| RQCD1 | 1 |
| RRAGB | 1 |
| RRM1 | 1 |
| RRP9 | 1 |
| RSAD2 | 1 |
| RSBN1 | 1 |
| RSL24D1 | 1 |
| RSPH9 | 1 |
| RSPO2 | 1 |
| RSU1 | 1 |
| RTCB | 1 |
| RTF1 | 1 |
| RTN2 | 1 |
| RTN3 | 1 |
| RTP4 | 1 |
| RUFY3 | 1 |
| RUNDC2C | 1 |
| RUNDC3A | 1 |
| RUNX1T1 | 1 |
| RUNX2 | 1 |
| RUVBL2 | 1 |
| RWDD1 | 1 |
| RWDD2A | 1 |
| RWDD4 | 1 |
| RYK | 1 |
| S100A13 | 1 |
| S100A16 | 1 |
| S100A6 | 1 |
| S100B | 1 |
| S100PBP | 1 |
| SAA4 | 1 |
| SACM1L | 1 |
| SACS | 1 |
| SAFB | 1 |
| SAFB2 | 1 |
| SALL2 | 1 |
| SALL3 | 1 |
| SAMD14 | 1 |
| SAMD3 | 1 |
| SAMD9 | 1 |
| SAP25 | 1 |
| SAP30L | 1 |
| SAPCD2 | 1 |
| SAT1 | 1 |
| SATB2 | 1 |
| SBF1 | 1 |
| SBK1 | 1 |
| SBNO1 | 1 |
| SBSN | 1 |
| SC5DL | 1 |
| SCAMP1 | 1 |
| SCAMP2 | 1 |
| SCAMP5 | 1 |
| SCAP | 1 |
| SCAPER | 1 |
| SCARA5 | 1 |
| SCARB1 | 1 |
| SCARB2 | 1 |
| SCARF1 | 1 |
| SCARF2 | 1 |
| SCARNA18B | 1 |
| SCG2 | 1 |
| SCGB3A2 | 1 |
| SCLY | 1 |
| SCN4B | 1 |
| SCN7A | 1 |
| SCN9A | 1 |
| SCNM1 | 1 |
| SCO2 | 1 |
| SCRN1 | 1 |
| SCRN3 | 1 |
| SCT | 1 |
| SCYL1BP1 | 1 |
| SDAD1 | 1 |
| SDC1 | 1 |
| SDC4 | 1 |
| SDCBP2 | 1 |
| SDCCAG1 | 1 |
| SDHA | 1 |
| SDHAP2 | 1 |
| SDHAP3 | 1 |
| SDK1 | 1 |
| SDK2 | 1 |
| SDPR | 1 |
| SDSL | 1 |
| SEC1 | 1 |
| SEC11C | 1 |
| SEC13 | 1 |
| SEC14L2 | 1 |
| SEC22B | 1 |
| SEC23A | 1 |
| SEC23B | 1 |
| SEC23IP | 1 |
| SEC24A | 1 |
| SEC24D | 1 |
| SEC31A | 1 |
| SEC61G | 1 |
| SEC62 | 1 |
| SEC62-AS1 | 1 |
| SECISBP2 | 1 |
| SELI | 1 |
| SELK | 1 |
| SELL | 1 |
| SELM | 1 |
| SELO | 1 |
| SEMA3D | 1 |
| SEMA3E | 1 |
| SEMA3F | 1 |
| SEMA3G | 1 |
| SEMA4D | 1 |
| SEMA4F | 1 |
| SEMA5A | 1 |
| SEMA6A | 1 |
| SEMA6B | 1 |
| SENP5 | 1 |
| SENP7 | 1 |
| SEP2 | 1 |
| SEP4 | 1 |
| SERF1B | 1 |
| SERINC2 | 1 |
| SERINC3 | 1 |
| SERINC4 | 1 |
| SERPINA1 | 1 |
| SERPINA13 | 1 |
| SERPINA3 | 1 |
| SERPINB1 | 1 |
| SERPINB9 | 1 |
| SERPIND1 | 1 |
| SERPINE1 | 1 |
| SERPINF1 | 1 |
| SERPING1 | 1 |
| SERPINI1 | 1 |
| SERTAD4-AS1 | 1 |
| SESN2 | 1 |
| SESTD1 | 1 |
| SETD1A | 1 |
| SEZ6L2 | 1 |
| SF3A1 | 1 |
| SF3A2 | 1 |
| SF3B14 | 1 |
| SF3B3 | 1 |
| SF3B4 | 1 |
| SFMBT1 | 1 |
| SFMBT2 | 1 |
| SFN | 1 |
| SFRS14 | 1 |
| SFRS2IP | 1 |
| SFRS4 | 1 |
| SFRS6 | 1 |
| SFT2D1 | 1 |
| SFTPD | 1 |
| SFXN4 | 1 |
| SFXN5 | 1 |
| SGIP1 | 1 |
| SGK3 | 1 |
| SGMS2 | 1 |
| SGOL2 | 1 |
| SGPL1 | 1 |
| SGPP2 | 1 |
| SGSH | 1 |
| SH2B2 | 1 |
| SH2D1A | 1 |
| SH2D1B | 1 |
| SH2D7 | 1 |
| SH3BGRL | 1 |
| SH3BGRL3 | 1 |
| SH3GLB2 | 1 |
| SH3PXD2A | 1 |
| SH3TC1 | 1 |
| SH3YL1 | 1 |
| SHANK2 | 1 |
| SHC1 | 1 |
| SHD | 1 |
| SHFM1 | 1 |
| SHISA6 | 1 |
| SHISA9 | 1 |
| SHOC2 | 1 |
| SHOX2 | 1 |
| SIAH1 | 1 |
| SIAH3 | 1 |
| SIDT2 | 1 |
| SIGIRR | 1 |
| SIK3 | 1 |
| SIN3A | 1 |
| SIPA1 | 1 |
| SIPA1L3 | 1 |
| SIRPA | 1 |
| SIRPB1 | 1 |
| SIRPD | 1 |
| SIRT5 | 1 |
| SIRT7 | 1 |
| SIRTI | 1 |
| SIX4 | 1 |
| SIX5 | 1 |
| SKA2 | 1 |
| SKIDA1 | 1 |
| SKP1 | 1 |
| SLAMF1 | 1 |
| SLC10A1 | 1 |
| SLC10A2 | 1 |
| SLC10A4 | 1 |
| SLC11A1 | 1 |
| SLC11A2 | 1 |
| SLC12A7 | 1 |
| SLC13A5 | 1 |
| SLC15A3 | 1 |
| SLC16A10 | 1 |
| SLC16A11 | 1 |
| SLC16A2 | 1 |
| SLC16A9 | 1 |
| SLC17A7 | 1 |
| SLC17A8 | 1 |
| SLC18A3 | 1 |
| SLC1A6 | 1 |
| SLC20A1 | 1 |
| SLC22A10 | 1 |
| SLC22A17 | 1 |
| SLC22A18 | 1 |
| SLC22A18AS | 1 |
| SLC22A25 | 1 |
| SLC23A2 | 1 |
| SLC24A6 | 1 |
| SLC25A10 | 1 |
| SLC25A11 | 1 |
| SLC25A12 | 1 |
| SLC25A17 | 1 |
| SLC25A2 | 1 |
| SLC25A23 | 1 |
| SLC25A28 | 1 |
| SLC25A29 | 1 |
| SLC25A3 | 1 |
| SLC25A30 | 1 |
| SLC25A33 | 1 |
| SLC25A39 | 1 |
| SLC25A40 | 1 |
| SLC25A42 | 1 |
| SLC25A6 | 1 |
| SLC26A11 | 1 |
| SLC27A1 | 1 |
| SLC27A3 | 1 |
| SLC27A4 | 1 |
| SLC29A1 | 1 |
| SLC2A1 | 1 |
| SLC2A13 | 1 |
| SLC2A3 | 1 |
| SLC2A4RG | 1 |
| SLC30A10 | 1 |
| SLC30A9 | 1 |
| SLC32A1 | 1 |
| SLC33A1 | 1 |
| SLC35A1 | 1 |
| SLC35A2 | 1 |
| SLC35A3 | 1 |
| SLC35A4 | 1 |
| SLC35A5 | 1 |
| SLC35B1 | 1 |
| SLC35B2 | 1 |
| SLC35C2 | 1 |
| SLC35E2B | 1 |
| SLC35F2 | 1 |
| SLC35F5 | 1 |
| SLC35G1 | 1 |
| SLC35G2 | 1 |
| SLC37A1 | 1 |
| SLC38A10 | 1 |
| SLC38A2 | 1 |
| SLC38A3 | 1 |
| SLC38A5 | 1 |
| SLC38A8 | 1 |
| SLC38A9 | 1 |
| SLC39A12 | 1 |
| SLC39A13 | 1 |
| SLC39A5 | 1 |
| SLC39A8 | 1 |
| SLC3A2 | 1 |
| SLC40A1 | 1 |
| SLC44A1 | 1 |
| SLC44A2 | 1 |
| SLC45A3 | 1 |
| SLC46A3 | 1 |
| SLC47A1 | 1 |
| SLC4A1 | 1 |
| SLC4A10 | 1 |
| SLC4A1AP | 1 |
| SLC4A2 | 1 |
| SLC4A5 | 1 |
| SLC5A2 | 1 |
| SLC5A6 | 1 |
| SLC5A7 | 1 |
| SLC5A8 | 1 |
| SLC5A9 | 1 |
| SLC6A10P | 1 |
| SLC6A11 | 1 |
| SLC6A13 | 1 |
| SLC6A15 | 1 |
| SLC6A17 | 1 |
| SLC7A10 | 1 |
| SLC7A2 | 1 |
| SLC7A5 | 1 |
| SLC7A7 | 1 |
| SLC8A1-AS1 | 1 |
| SLC9A1 | 1 |
| SLC9A2 | 1 |
| SLC9A3R1 | 1 |
| SLC9B1 | 1 |
| SLCO2A1 | 1 |
| SLCO2B1 | 1 |
| SLCO3A1 | 1 |
| SLCO4A1 | 1 |
| SLCO4C1 | 1 |
| SLCO5A1 | 1 |
| SLFN5 | 1 |
| SLITRK1 | 1 |
| SLITRK4 | 1 |
| SLITRK5 | 1 |
| SLMAP | 1 |
| SLN | 1 |
| SMAD4 | 1 |
| SMAD5-AS1 | 1 |
| SMAD5OS | 1 |
| SMAD9 | 1 |
| SMAP2 | 1 |
| SMARCA4 | 1 |
| SMARCC2 | 1 |
| SMARCD2 | 1 |
| SMC1A | 1 |
| SMC4 | 1 |
| SMCHD1 | 1 |
| SMG7 | 1 |
| SMIM2-AS1 | 1 |
| SMN1 | 1 |
| SMNDC1 | 1 |
| SMO | 1 |
| SMPD1 | 1 |
| SMPD3 | 1 |
| SMR3B | 1 |
| SMS | 1 |
| SMTN | 1 |
| SMU1 | 1 |
| SMUG1 | 1 |
| SMYD5 | 1 |
| SNAP29 | 1 |
| SNAPC1 | 1 |
| SNAPC5 | 1 |
| SNCG | 1 |
| SND1 | 1 |
| SNHG1 | 1 |
| SNHG11 | 1 |
| SNHG14 | 1 |
| SNORA10 | 1 |
| SNORA11D | 1 |
| SNORA11E | 1 |
| SNORA15 | 1 |
| SNORA18 | 1 |
| SNORA25 | 1 |
| SNORA31 | 1 |
| SNORA33 | 1 |
| SNORA40 | 1 |
| SNORA48 | 1 |
| SNORA52 | 1 |
| SNORA53 | 1 |
| SNORA57 | 1 |
| SNORA60 | 1 |
| SNORA64 | 1 |
| SNORA67 | 1 |
| SNORA73A | 1 |
| SNORA76 | 1 |
| SNORA7A | 1 |
| SNORA7B | 1 |
| SNORA8 | 1 |
| SNORD100 | 1 |
| SNORD14D | 1 |
| SNORD14E | 1 |
| SNORD17 | 1 |
| SNORD34 | 1 |
| SNORD83B | 1 |
| SNORD94 | 1 |
| SNPH | 1 |
| SNRNP48 | 1 |
| SNRP70 | 1 |
| SNRPB | 1 |
| SNRPB2 | 1 |
| SNRPD2 | 1 |
| SNRPD3 | 1 |
| SNRPF | 1 |
| SNRPG | 1 |
| SNTA1 | 1 |
| SNURF | 1 |
| SNW1 | 1 |
| SNX11 | 1 |
| SNX14 | 1 |
| SNX15 | 1 |
| SNX21 | 1 |
| SNX32 | 1 |
| SNX8 | 1 |
| SOAT1 | 1 |
| SOCS1 | 1 |
| SOCS2 | 1 |
| SOCS2-AS1 | 1 |
| SOD1 | 1 |
| SOD3 | 1 |
| SOGA3 | 1 |
| SOHLH1 | 1 |
| SON | 1 |
| SORCS3 | 1 |
| SORL1 | 1 |
| SOS1 | 1 |
| SOS1-IT1 | 1 |
| SOSTDC1 | 1 |
| SOX1 | 1 |
| SOX13 | 1 |
| SOX18 | 1 |
| SOX5 | 1 |
| SOX9 | 1 |
| SP2 | 1 |
| SP3 | 1 |
| SPAG1 | 1 |
| SPARC | 1 |
| SPARCL1 | 1 |
| SPATA20 | 1 |
| SPATA2L | 1 |
| SPATC1 | 1 |
| SPCS2 | 1 |
| SPCS3 | 1 |
| SPEF1 | 1 |
| SPHAR | 1 |
| SPICE1 | 1 |
| SPIN1 | 1 |
| SPIN3 | 1 |
| SPINT2 | 1 |
| SPN | 1 |
| SPOCD1 | 1 |
| SPOCK3 | 1 |
| SPON2 | 1 |
| SPP1 | 1 |
| SPPL2A | 1 |
| SPPL2B | 1 |
| SPRY3 | 1 |
| SPRY4 | 1 |
| SPRYD3 | 1 |
| SPSB1 | 1 |
| SPSB2 | 1 |
| SPSB3 | 1 |
| SPTAN1 | 1 |
| SPTBN1 | 1 |
| SPTY2D1 | 1 |
| SQRDL | 1 |
| SQSTM1 | 1 |
| SR140 | 1 |
| SREK1 | 1 |
| SRMS | 1 |
| SRP14 | 1 |
| SRP14P1 | 1 |
| SRP19 | 1 |
| SRP72 | 1 |
| SRPK2 | 1 |
| SRPR | 1 |
| SRPRB | 1 |
| SRRM1 | 1 |
| SRRM3 | 1 |
| SRSF10 | 1 |
| SS18L2 | 1 |
| SSB | 1 |
| SSBP1 | 1 |
| SSBP4 | 1 |
| SSH1 | 1 |
| SSH2 | 1 |
| SSPO | 1 |
| SSR1 | 1 |
| SSR2 | 1 |
| SSR3 | 1 |
| SSTR2 | 1 |
| SSTR3 | 1 |
| ST3GAL2 | 1 |
| ST6GALNAC1 | 1 |
| ST6GALNAC4 | 1 |
| ST6GALNAC6 | 1 |
| ST70T2 | 1 |
| ST8SIA3 | 1 |
| ST8SIA4 | 1 |
| STAG3 | 1 |
| STAG3L2 | 1 |
| STAG3L3 | 1 |
| STAMBPL1 | 1 |
| STAP2 | 1 |
| STARD10 | 1 |
| STARD8 | 1 |
| STAT2 | 1 |
| STAT3 | 1 |
| STAT6 | 1 |
| STK10 | 1 |
| STK24 | 1 |
| STK25 | 1 |
| STK32A | 1 |
| STMN2 | 1 |
| STMN3 | 1 |
| STOML3 | 1 |
| STOX2 | 1 |
| STRADB | 1 |
| STRAP | 1 |
| STRBP | 1 |
| STRN4 | 1 |
| STT3A | 1 |
| STUB1 | 1 |
| STX10 | 1 |
| STX16 | 1 |
| STX16-NPEPL1 | 1 |
| STX2 | 1 |
| STX4 | 1 |
| STX6 | 1 |
| STX7 | 1 |
| STXBP2 | 1 |
| STXIO | 1 |
| STYK1 | 1 |
| SUB1 | 1 |
| SUCLA2 | 1 |
| SUCLG2 | 1 |
| SUCNR1 | 1 |
| SUCO | 1 |
| SUGT1 | 1 |
| SULF1 | 1 |
| SULT1A2 | 1 |
| SUMO1P3 | 1 |
| SUMO3 | 1 |
| SUMO4 | 1 |
| SUOX | 1 |
| SUPT3H | 1 |
| SUPT5H | 1 |
| SURF2 | 1 |
| SURF4 | 1 |
| SURF6 | 1 |
| SUSD2 | 1 |
| SUSD3 | 1 |
| SUZ12 | 1 |
| SV2A | 1 |
| SVOP | 1 |
| SYCE2 | 1 |
| SYCP3 | 1 |
| SYF2 | 1 |
| SYN1 | 1 |
| SYN2 | 1 |
| SYNDIG1 | 1 |
| SYNDIG1L | 1 |
| SYNGR3 | 1 |
| SYNJ1 | 1 |
| SYNM | 1 |
| SYNPO | 1 |
| SYNPR | 1 |
| SYNRG | 1 |
| SYT14 | 1 |
| SYT17 | 1 |
| SYT2 | 1 |
| SYT3 | 1 |
| SYT6 | 1 |
| SYTL2 | 1 |
| SYTL3 | 1 |
| SYTL5 | 1 |
| SYVN1 | 1 |
| TAC1 | 1 |
| TAC3 | 1 |
| TACC1 | 1 |
| TACR3 | 1 |
| TADA3 | 1 |
| TAF10 | 1 |
| TAF1C | 1 |
| TAF7 | 1 |
| TAF9BP1 | 1 |
| TAGLN3 | 1 |
| TAL1 | 1 |
| TANGO2 | 1 |
| TAOK1 | 1 |
| TAPBPL | 1 |
| TAPT1-AS1 | 1 |
| TARBP2 | 1 |
| TARSL2 | 1 |
| TAS2R30 | 1 |
| TAS2R43 | 1 |
| TBC1D10B | 1 |
| TBC1D10C | 1 |
| TBC1D2 | 1 |
| TBC1D26 | 1 |
| TBC1D7 | 1 |
| TBC1D8B | 1 |
| TBCA | 1 |
| TBCB | 1 |
| TBCD | 1 |
| TBCID12 | 1 |
| TBCIDIOB | 1 |
| TBK1 | 1 |
| TBKBP1 | 1 |
| TBR1 | 1 |
| TBX3 | 1 |
| TBXA2R | 1 |
| TC2N | 1 |
| TCAP | 1 |
| TCEA2 | 1 |
| TCEAL2 | 1 |
| TCEAL3 | 1 |
| TCEAL4 | 1 |
| TCEAL5 | 1 |
| TCEAL6 | 1 |
| TCEB1 | 1 |
| TCERG1 | 1 |
| TCF3 | 1 |
| TCPIOL | 1 |
| TCTE3 | 1 |
| TCTEX1D4 | 1 |
| TDG | 1 |
| TDP1 | 1 |
| TDRD7 | 1 |
| TEAD2 | 1 |
| TEAD3 | 1 |
| TEC | 1 |
| TECPR1 | 1 |
| TEKT4P2 | 1 |
| TENM1 | 1 |
| TENM3 | 1 |
| TERF1 | 1 |
| TERF2 | 1 |
| TES | 1 |
| TESPA1 | 1 |
| TET3 | 1 |
| TEX10 | 1 |
| TEX14 | 1 |
| TEX15 | 1 |
| TEX264 | 1 |
| TF | 1 |
| TFAM | 1 |
| TFCP2 | 1 |
| TFEB | 1 |
| TFF3 | 1 |
| TFR2 | 1 |
| TFRC | 1 |
| TGFB1 | 1 |
| TGFB3 | 1 |
| TGFBRAP1 | 1 |
| TGIF1 | 1 |
| TGIF2 | 1 |
| TGOLN2 | 1 |
| THAP10 | 1 |
| THAP11 | 1 |
| THAP2 | 1 |
| THAP4 | 1 |
| THAP7-AS1 | 1 |
| THBD | 1 |
| THBS1 | 1 |
| THBS4 | 1 |
| THEM2 | 1 |
| THEMIS | 1 |
| THNSL1 | 1 |
| THOC4 | 1 |
| THRB | 1 |
| THSD4 | 1 |
| THTPA | 1 |
| THUMPD1 | 1 |
| THY1 | 1 |
| TIA1 | 1 |
| TIAL1 | 1 |
| TIAM1 | 1 |
| TIE1 | 1 |
| TIMM13 | 1 |
| TIMM29 | 1 |
| TIMM50 | 1 |
| TIMP2 | 1 |
| TIMP4 | 1 |
| TINF2 | 1 |
| TINP1 | 1 |
| TJP3 | 1 |
| TLK1 | 1 |
| TLL1 | 1 |
| TLN1 | 1 |
| TLR2 | 1 |
| TLR4 | 1 |
| TLR9 | 1 |
| TM4SF1 | 1 |
| TM4SF18 | 1 |
| TM4SF4 | 1 |
| TM7SF4 | 1 |
| TMBIM1 | 1 |
| TMBIM6 | 1 |
| TMC4 | 1 |
| TMC6 | 1 |
| TMCC1 | 1 |
| TMCC2 | 1 |
| TMCO1 | 1 |
| TMED10P | 1 |
| TMEFF2 | 1 |
| TMEM106A | 1 |
| TMEM106C | 1 |
| TMEM108 | 1 |
| TMEM126B | 1 |
| TMEM127 | 1 |
| TMEM129 | 1 |
| TMEM132D | 1 |
| TMEM134 | 1 |
| TMEM135 | 1 |
| TMEM137 | 1 |
| TMEM138 | 1 |
| TMEM140 | 1 |
| TMEM14D | 1 |
| TMEM14E | 1 |
| TMEM150C | 1 |
| TMEM160 | 1 |
| TMEM167B | 1 |
| TMEM175 | 1 |
| TMEM177 | 1 |
| TMEM178 | 1 |
| TMEM18 | 1 |
| TMEM180 | 1 |
| TMEM182 | 1 |
| TMEM184A | 1 |
| TMEM188 | 1 |
| TMEM19 | 1 |
| TMEM190 | 1 |
| TMEM191A | 1 |
| TMEM191C | 1 |
| TMEM194 | 1 |
| TMEM2 | 1 |
| TMEM203 | 1 |
| TMEM214 | 1 |
| TMEM215 | 1 |
| TMEM219 | 1 |
| TMEM26 | 1 |
| TMEM263 | 1 |
| TMEM35 | 1 |
| TMEM37 | 1 |
| TMEM39A | 1 |
| TMEM39B | 1 |
| TMEM4 | 1 |
| TMEM41A | 1 |
| TMEM41B | 1 |
| TMEM42 | 1 |
| TMEM50B | 1 |
| TMEM59L | 1 |
| TMEM62 | 1 |
| TMEM64 | 1 |
| TMEM70 | 1 |
| TMEM81 | 1 |
| TMEM87A | 1 |
| TMEM88 | 1 |
| TMEM93 | 1 |
| TMF1 | 1 |
| TMIGDI | 1 |
| TMOD2 | 1 |
| TMPRSS11D | 1 |
| TMSB10 | 1 |
| TMSB4XP2 | 1 |
| TMSL3 | 1 |
| TMTC3 | 1 |
| TMUB2 | 1 |
| TMX1 | 1 |
| TNFAIP3 | 1 |
| TNFAIP8 | 1 |
| TNFRSF11A | 1 |
| TNFRSF12A | 1 |
| TNFRSF13C | 1 |
| TNFRSF14 | 1 |
| TNFRSF17 | 1 |
| TNFRSF18 | 1 |
| TNFRSF1A | 1 |
| TNFRSF1B | 1 |
| TNFRSF25 | 1 |
| TNFRSF6B | 1 |
| TNFRSF9 | 1 |
| TNFSF13 | 1 |
| TNFSF15 | 1 |
| TNFSF9 | 1 |
| TNIP1 | 1 |
| TNIP2 | 1 |
| TNKS | 1 |
| TNPO3 | 1 |
| TNRC18 | 1 |
| TNS1 | 1 |
| TNS3 | 1 |
| TOM1 | 1 |
| TOM1L1 | 1 |
| TOM1L2 | 1 |
| TOMM20 | 1 |
| TOMM20L | 1 |
| TOMM6 | 1 |
| TOMM7 | 1 |
| TONSL | 1 |
| TOP3A | 1 |
| TOP3B | 1 |
| TOPORS | 1 |
| TOX3 | 1 |
| TP53AIP1 | 1 |
| TP53BP2 | 1 |
| TP53I3 | 1 |
| TP53INP1 | 1 |
| TPCN1 | 1 |
| TPD52 | 1 |
| TPK1 | 1 |
| TPM4 | 1 |
| TPP1 | 1 |
| TPSG1 | 1 |
| TPX2 | 1 |
| TRAF1 | 1 |
| TRAF2 | 1 |
| TRAF3IP3 | 1 |
| TRAF5 | 1 |
| TRAF6 | 1 |
| TRAFD1 | 1 |
| TRAK1 | 1 |
| TRAM2 | 1 |
| TRAPPC2L | 1 |
| TRBC1 | 1 |
| TRBC2 | 1 |
| TRBV19 | 1 |
| TRBV7-4 | 1 |
| TRBV7-6 | 1 |
| TRBV7-7 | 1 |
| TRBV7-8 | 1 |
| TREML2 | 1 |
| TREX1 | 1 |
| TRIB1 | 1 |
| TRIM16 | 1 |
| TRIM21 | 1 |
| TRIM24 | 1 |
| TRIM25 | 1 |
| TRIM28 | 1 |
| TRIM36-IT1 | 1 |
| TRIM38 | 1 |
| TRIM39 | 1 |
| TRIM47 | 1 |
| TRIM52 | 1 |
| TRIM58 | 1 |
| TRIM71 | 1 |
| TRIM72 | 1 |
| TRIM8 | 1 |
| TRIP12 | 1 |
| TRIPI 1 | 1 |
| TRMT112 | 1 |
| TRMT2A | 1 |
| TRMT5 | 1 |
| TROVE2 | 1 |
| TRPC4 | 1 |
| TRPC4AP | 1 |
| TRPC5 | 1 |
| TRPT1 | 1 |
| TRRAP | 1 |
| TSC22D4 | 1 |
| TSEN54 | 1 |
| TSHR | 1 |
| TSNARE1 | 1 |
| TSNAXIP1 | 1 |
| TSPAN10 | 1 |
| TSPAN14 | 1 |
| TSPAN16 | 1 |
| TSPAN18 | 1 |
| TSPAN2 | 1 |
| TSPAN33 | 1 |
| TSPAN5 | 1 |
| TSPO | 1 |
| TSPYL3 | 1 |
| TSPYL4 | 1 |
| TSR3 | 1 |
| TSSK1B | 1 |
| TSSK6 | 1 |
| TST | 1 |
| TTBK2 | 1 |
| TTBKI | 1 |
| TTC21A | 1 |
| TTC3 | 1 |
| TTC38 | 1 |
| TTC6 | 1 |
| TTC8 | 1 |
| TTC9B | 1 |
| TTLL1 | 1 |
| TTLL11 | 1 |
| TTLL3 | 1 |
| TTLL6 | 1 |
| TTN | 1 |
| TTN-AS1 | 1 |
| TTTY14 | 1 |
| TTYH3 | 1 |
| TUBA4A | 1 |
| TUBB1 | 1 |
| TUBB2A | 1 |
| TUBB2B | 1 |
| TUBB3 | 1 |
| TUBB6 | 1 |
| TUBBP5 | 1 |
| TUBGCP5 | 1 |
| TUBGCP6 | 1 |
| TUG1 | 1 |
| TUSC3 | 1 |
| TXLNG | 1 |
| TXNDC5 | 1 |
| TXNIP | 1 |
| TXNL1 | 1 |
| TXNRD2 | 1 |
| TYK2 | 1 |
| TYMP | 1 |
| TYW1 | 1 |
| TYW3 | 1 |
| U2AF1 | 1 |
| U2AF1L2 | 1 |
| U2AF1L4 | 1 |
| U82695.10 | 1 |
| UAP1L1 | 1 |
| UBA1 | 1 |
| UBA3 | 1 |
| UBA52P6 | 1 |
| UBAP2L | 1 |
| UBASH3B | 1 |
| UBB | 1 |
| UBC | 1 |
| UBD | 1 |
| UBE1 | 1 |
| UBE2C | 1 |
| UBE2D1 | 1 |
| UBE2E1 | 1 |
| UBE2H | 1 |
| UBE2I | 1 |
| UBE2J2 | 1 |
| UBE2L3 | 1 |
| UBE2L6 | 1 |
| UBE2N | 1 |
| UBE2Q1 | 1 |
| UBE2Q2 | 1 |
| UBE2Q2P2 | 1 |
| UBE2Q2P6 | 1 |
| UBE2QL1 | 1 |
| UBE2W | 1 |
| UBE3A | 1 |
| UBE4B | 1 |
| UBL3 | 1 |
| UBL4A | 1 |
| UBL5 | 1 |
| UBLCP1 | 1 |
| UBN1 | 1 |
| UBQLN2 | 1 |
| UBTD1 | 1 |
| UBTF | 1 |
| UBXD2 | 1 |
| UBXN11 | 1 |
| UBXN2A | 1 |
| UBXN7 | 1 |
| UCHL3 | 1 |
| UCK1 | 1 |
| UCK2 | 1 |
| UCKL1 | 1 |
| UCP3 | 1 |
| UFD1L | 1 |
| UFL1 | 1 |
| UFSP1 | 1 |
| UGCGL1 | 1 |
| UGGT1 | 1 |
| UGP2 | 1 |
| UGT8 | 1 |
| UHMK1 | 1 |
| UIMC1 | 1 |
| ULK1 | 1 |
| ULK3 | 1 |
| UNC119 | 1 |
| UNC5C | 1 |
| UNC5D | 1 |
| UNC84A | 1 |
| UPB1 | 1 |
| UPK3BL | 1 |
| UPLP | 1 |
| UQCRC1 | 1 |
| UQCRHL | 1 |
| URB2 | 1 |
| URG4 | 1 |
| URM1 | 1 |
| UROS | 1 |
| USHBP1 | 1 |
| USMG5 | 1 |
| USO1 | 1 |
| USP15 | 1 |
| USP18 | 1 |
| USP21 | 1 |
| USP31 | 1 |
| USP37 | 1 |
| USP4 | 1 |
| USP46 | 1 |
| USP47 | 1 |
| USP48 | 1 |
| USP49 | 1 |
| USP5 | 1 |
| USP8 | 1 |
| UTP20 | 1 |
| UTS2 | 1 |
| UTX | 1 |
| VAC14 | 1 |
| VAMP1 | 1 |
| VAMP2 | 1 |
| VAMP4 | 1 |
| VAMP7 | 1 |
| VARS2 | 1 |
| VASH1 | 1 |
| VAT1L | 1 |
| VAV1 | 1 |
| VBP1 | 1 |
| VCL | 1 |
| VCP | 1 |
| VCPIP1 | 1 |
| VDAC1 | 1 |
| VDP | 1 |
| VDR | 1 |
| VGF | 1 |
| VKORC1 | 1 |
| VLDLR | 1 |
| VPS11 | 1 |
| VPS13B | 1 |
| VPS13D | 1 |
| VPS26A | 1 |
| VPS26B | 1 |
| VPS29 | 1 |
| VPS36 | 1 |
| VPS39 | 1 |
| VPS41 | 1 |
| VPS45 | 1 |
| VPS51 | 1 |
| VRK1 | 1 |
| VRK2 | 1 |
| VRK3 | 1 |
| VSIG2 | 1 |
| VSIG4 | 1 |
| VSTM2A | 1 |
| VSTM2B | 1 |
| VWA3A | 1 |
| VWA7 | 1 |
| VWF | 1 |
| WARS | 1 |
| WASH2P | 1 |
| WASH3P | 1 |
| WASH7P | 1 |
| WASL | 1 |
| WBP1L | 1 |
| WBP4 | 1 |
| WBSCRI | 1 |
| WDFY1 | 1 |
| WDFY4 | 1 |
| WDR12 | 1 |
| WDR16 | 1 |
| WDR18 | 1 |
| WDR19 | 1 |
| WDR20 | 1 |
| WDR22 | 1 |
| WDR23 | 1 |
| WDR33 | 1 |
| WDR34 | 1 |
| WDR37 | 1 |
| WDR38 | 1 |
| WDR47 | 1 |
| WDR55 | 1 |
| WDR6 | 1 |
| WDR61 | 1 |
| WDR66 | 1 |
| WDR67 | 1 |
| WDR85 | 1 |
| WDR86 | 1 |
| WDR96 | 1 |
| WFDC1 | 1 |
| WFDC8 | 1 |
| WHDC1 | 1 |
| WHSC1 | 1 |
| WIPF3 | 1 |
| WIPI1 | 1 |
| WISP2 | 1 |
| WISP3 | 1 |
| WIZ | 1 |
| WNK1 | 1 |
| WNT11 | 1 |
| WNT2B | 1 |
| WNT3 | 1 |
| WSB2 | 1 |
| WWC2 | 1 |
| WWP1 | 1 |
| WWPI | 1 |
| WWTR1 | 1 |
| XAB2 | 1 |
| XCL1, XCL2 | 1 |
| XKR4 | 1 |
| XKR8 | 1 |
| XLOC_000643 | 1 |
| XLOC_000683 | 1 |
| XLOC_000782 | 1 |
| XLOC_000852 | 1 |
| XLOC_000992 | 1 |
| XLOC_001035 | 1 |
| XLOC_001592 | 1 |
| XLOC_002996 | 1 |
| XLOC_003228 | 1 |
| XLOC_003343 | 1 |
| XLOC_004577 | 1 |
| XLOC_004700 | 1 |
| XLOC_004908 | 1 |
| XLOC_005131 | 1 |
| XLOC_005784 | 1 |
| XLOC_005935 | 1 |
| XLOC_005958 | 1 |
| XLOC_006291 | 1 |
| XLOC_006505 | 1 |
| XLOC_007308 | 1 |
| XLOC_007538 | 1 |
| XLOC_007714 | 1 |
| XLOC_008079 | 1 |
| XLOC_008630 | 1 |
| XLOC_009017 | 1 |
| XLOC_009021 | 1 |
| XLOC_009146 | 1 |
| XLOC_010556 | 1 |
| XLOC_010860 | 1 |
| XLOC_011183 | 1 |
| XLOC_011928 | 1 |
| XLOC_012046 | 1 |
| XLOC_012496 | 1 |
| XLOC_012534 | 1 |
| XLOC_012568 | 1 |
| XLOC_013300 | 1 |
| XLOC_013480 | 1 |
| XLOC_013534 | 1 |
| XLOC_013704 | 1 |
| XLOC_014105 | 1 |
| XLOC_014121 | 1 |
| XLOC_014162 | 1 |
| XLOC_014351 | 1 |
| XPC | 1 |
| XPNPEP3 | 1 |
| XPO1 | 1 |
| XPO4 | 1 |
| XPO7 | 1 |
| XRCC6 | 1 |
| XRRAI | 1 |
| XXYLT1 | 1 |
| YARS2 | 1 |
| YBX3 | 1 |
| YIF1B | 1 |
| YIPF1 | 1 |
| YIPF5 | 1 |
| YME1L1 | 1 |
| YPEL1 | 1 |
| YRDC | 1 |
| YTHDC1 | 1 |
| YWHAE | 1 |
| YWHAH | 1 |
| ZACN | 1 |
| ZAK | 1 |
| ZAP70 | 1 |
| ZBED1 | 1 |
| ZBED6 | 1 |
| ZBP1 | 1 |
| ZBTB12B | 1 |
| ZBTB16 | 1 |
| ZBTB20 | 1 |
| ZBTB22 | 1 |
| ZBTB24 | 1 |
| ZBTB26 | 1 |
| ZBTB33 | 1 |
| ZBTB42 | 1 |
| ZBTB44 | 1 |
| ZBTB7B | 1 |
| ZBTB8B | 1 |
| ZBTB8OSP1 | 1 |
| ZC3H11A | 1 |
| ZC3H12A | 1 |
| ZC3H12D | 1 |
| ZCCHC11 | 1 |
| ZCCHC12 | 1 |
| ZCCHC2 | 1 |
| ZCCHC6 | 1 |
| ZCCHC7 | 1 |
| ZCCHC8 | 1 |
| ZCRBI | 1 |
| ZDHHC1 | 1 |
| ZDHHC11 | 1 |
| ZDHHC18 | 1 |
| ZDHHC2 | 1 |
| ZDHHC24 | 1 |
| ZDHHC3 | 1 |
| ZDHHC8 | 1 |
| ZEB1 | 1 |
| ZFAND2A | 1 |
| ZFAND2B | 1 |
| ZFAND5 | 1 |
| ZFAND6 | 1 |
| ZFC3H1 | 1 |
| ZFHX2 | 1 |
| ZFP36 | 1 |
| ZFP90 | 1 |
| ZFR | 1 |
| ZFYVE26 | 1 |
| ZIC1 | 1 |
| ZIC4 | 1 |
| ZMAT2 | 1 |
| ZMAT3 | 1 |
| ZMAT5 | 1 |
| ZMIZ2 | 1 |
| ZMYM6NB | 1 |
| ZMYND10 | 1 |
| ZMYND11 | 1 |
| ZNF134 | 1 |
| ZNF136 | 1 |
| ZNF140 | 1 |
| ZNF146 | 1 |
| ZNF148 | 1 |
| ZNF157 | 1 |
| ZNF16 | 1 |
| ZNF195 | 1 |
| ZNF197 | 1 |
| ZNF204P | 1 |
| ZNF209P | 1 |
| ZNF212 | 1 |
| ZNF215 | 1 |
| ZNF217 | 1 |
| ZNF219 | 1 |
| ZNF22 | 1 |
| ZNF223 | 1 |
| ZNF224 | 1 |
| ZNF233 | 1 |
| ZNF252P | 1 |
| ZNF26 | 1 |
| ZNF267 | 1 |
| ZNF280B | 1 |
| ZNF292 | 1 |
| ZNF294 | 1 |
| ZNF295 | 1 |
| ZNF296 | 1 |
| ZNF318 | 1 |
| ZNF32 | 1 |
| ZNF326 | 1 |
| ZNF33B | 1 |
| ZNF364 | 1 |
| ZNF37A | 1 |
| ZNF397 | 1 |
| ZNF408 | 1 |
| ZNF438 | 1 |
| ZNF444 | 1 |
| ZNF446 | 1 |
| ZNF451 | 1 |
| ZNF460 | 1 |
| ZNF469 | 1 |
| ZNF471 | 1 |
| ZNF483 | 1 |
| ZNF497 | 1 |
| ZNF500 | 1 |
| ZNF507 | 1 |
| ZNF516 | 1 |
| ZNF517 | 1 |
| ZNF518A | 1 |
| ZNF518B | 1 |
| ZNF519 | 1 |
| ZNF549 | 1 |
| ZNF557 | 1 |
| ZNF571 | 1 |
| ZNF577 | 1 |
| ZNF580 | 1 |
| ZNF581 | 1 |
| ZNF584 | 1 |
| ZNF585A | 1 |
| ZNF586 | 1 |
| ZNF596 | 1 |
| ZNF638 | 1 |
| ZNF649 | 1 |
| ZNF654 | 1 |
| ZNF658 | 1 |
| ZNF660 | 1 |
| ZNF668 | 1 |
| ZNF669 | 1 |
| ZNF688 | 1 |
| ZNF692 | 1 |
| ZNF697 | 1 |
| ZNF700 | 1 |
| ZNF701 | 1 |
| ZNF703 | 1 |
| ZNF706 | 1 |
| ZNF707 | 1 |
| ZNF710 | 1 |
| ZNF729 | 1 |
| ZNF737 | 1 |
| ZNF74 | 1 |
| ZNF747 | 1 |
| ZNF763 | 1 |
| ZNF768 | 1 |
| ZNF770 | 1 |
| ZNF784 | 1 |
| ZNF786 | 1 |
| ZNF787 | 1 |
| ZNF808 | 1 |
| ZNF814 | 1 |
| ZNF815P | 1 |
| ZNF828 | 1 |
| ZNF83 | 1 |
| ZNF831 | 1 |
| ZNF835 | 1 |
| ZNF839 | 1 |
| ZNF90P1 | 1 |
| ZNF98 | 1 |
| ZNHIT3 | 1 |
| ZNHIT6 | 1 |
| ZRANB1 | 1 |
| ZRSR1 | 1 |
| ZSCAN1 | 1 |
| ZSCAN22 | 1 |
| ZSCAN23 | 1 |
| ZSCAN30 | 1 |
| ZSWIM6 | 1 |
| ZYG11B | 1 |
